# Supplementary material for: Iron can be microbially extracted from Lunar and Martian regolith simulants and 3D printed into tough structural materials
Source: PLoS One. 2021 Apr 28;16(4):e0249962. doi: 10.1371/journal.pone.0249962 (PMC8081250; doi:10.1371/journal.pone.0249962)
Supplement: S1 Data — (ZIP) [file pone.0249962.s001.zip › Data_updated/XRF,XRD/XRD_EAC1_JSC2A_untreated_and_treated_23may19.pdf]

X-RAY FACILITIES GROUP

Dr. Amarante Böttger A.J.Bottger@tudelft.nl
phone +31(0)1527·82243
Dhr. Ruud Hendrikx R.W.A.Hendrikx@tudelft.nl
Drs. Richard Huizenga R.M.Huizenga@tudelft.nl

Delft University of Technology, Faculty of 3mE
Department of Materials Science and Engineering
Mekelweg 2, NL-2628 CD Delft, the Netherlands, phone +31(0)1527·82255/89459

XRD measurements of lunar regolith simulants

Author	:	Ruud Hendrikx
Date	:	23 may 19
Researcher	:	Benjamin Lehner. L&R
Research question	:	Phase identification

"For sustainable space exploration it is necessary to extract resource directly from the planet/moon we are going to. This process is called "in situ resource utilization (ISRU)". We developed a methodology to extract iron from different lunar regolith simulants using microbiology and magnetism".

Samples
The samples are iron oxide-containing powders.

Specimen
A small amount of powder was deposited as a thin layer on a Si510 wafer from a powder-isopropanol suspension.

Experimental
Instrument: Bruker D8 Advance diffractometer Bragg-Brentano geometry and Lynxeye position sensitive detector. Cu Kα radiation. Divergence slit V12, scatter screen height 5 mm, 45 kV 40 mA. Sample spinning. Detector settings LL 0.19 W 0.06. Sample holder L510.

Measurements
Coupled θ -2θ scan 8° - 110°, step size 0.021 ° 2θ, counting time per step 1 s.

Data evaluation
Bruker software DiffracSuite.EVA vs 5.0.

Results
Figures 1 - 6 show the measured XRD patterns in black, after background subtraction and a small displacement correction. The colored sticks give the peak positions and intensities of the possibly present phases, using the ICDD pdf4 database, see table 1.

sample	*compound*	
1 0hr-JSC2-t	Anorthite	(Ca0.78Na0.22)(Al1.78Si0.22)Si2O8
	Magnetite	Fe+2Fe2+3O4
	Forsterite, ferroan	(Mg1.44Fe0.56)(SiO4)
2 48hrs-JSC2-t	Anorthite	(Ca0.78Na0.22)(Al1.78Si0.22)Si2O8
	Magnetite, syn	Fe+2Fe2+3O4
	Forsterite, ferroan	(Mg1.44Fe0.56)(SiO4)
3 48hrs-JSC2	Anorthite	(Ca0.78Na0.22)(Al1.78Si0.22)Si2O8
	Forsterite, ferroan	(Mg1.44Fe0.56)(SiO4)
4 0hr-JSC2	Anorthite	(Ca0.78Na0.22)(Al1.78Si0.22)Si2O8
	Forsterite, ferroan	(Mg1.44Fe0.56)(SiO4)
5 48hrs-EAC2-t	Anorthite	(Ca0.78Na0.22)(Al1.78Si0.22)Si2O8
	Augite	Ca(Mg,Fe)Si2O6
	Manganese Iron Oxide	Mn0.43Fe2.57O4
	Forsterite, ferroan	(Mg1.44Fe0.56)(SiO4)
	Nepheline	K0.695Na3.03Ca0.03Fe0.04Al3.75Si0.21(SiO4)4
6 0hr-EAC1-t	Anorthite	(Ca0.78Na0.22)(Al1.78Si0.22)Si2O8
	Augite	Ca(Mg,Fe)Si2O6
	Manganese Iron Oxide	Mn0.43Fe2.57O4
	Forsterite, ferroan	(Mg1.44Fe0.56)(SiO4)
	Nepheline	K0.695Na3.03Ca0.03Fe0.04Al3.75Si0.21(SiO4)4

Table 1.

If the analysis is a significant part of a publication, a co-authorship is preferred.

In any case, it is useful to involve us in the preparation of any presentation to ensure optimum and correct use of the analysis results!

Whenever used in a publication, an acknowledgement will be appreciated, e.g.: "personX at the Department of Materials Science and Engineering of the Delft University of Technology is acknowledged for the X-ray analysis".

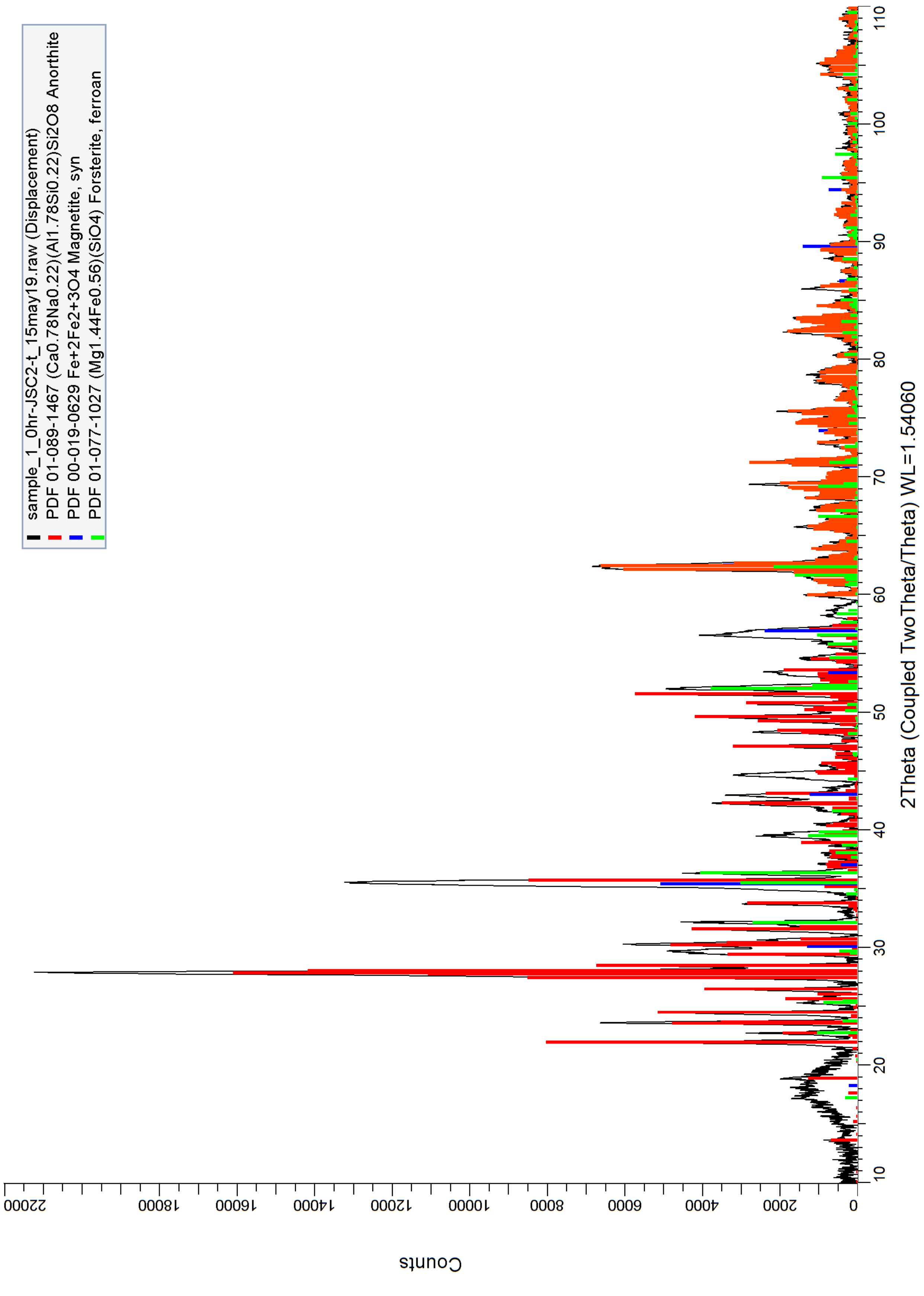

Figure 1 XRD pattern sample "1 0hr-JSC2-t "

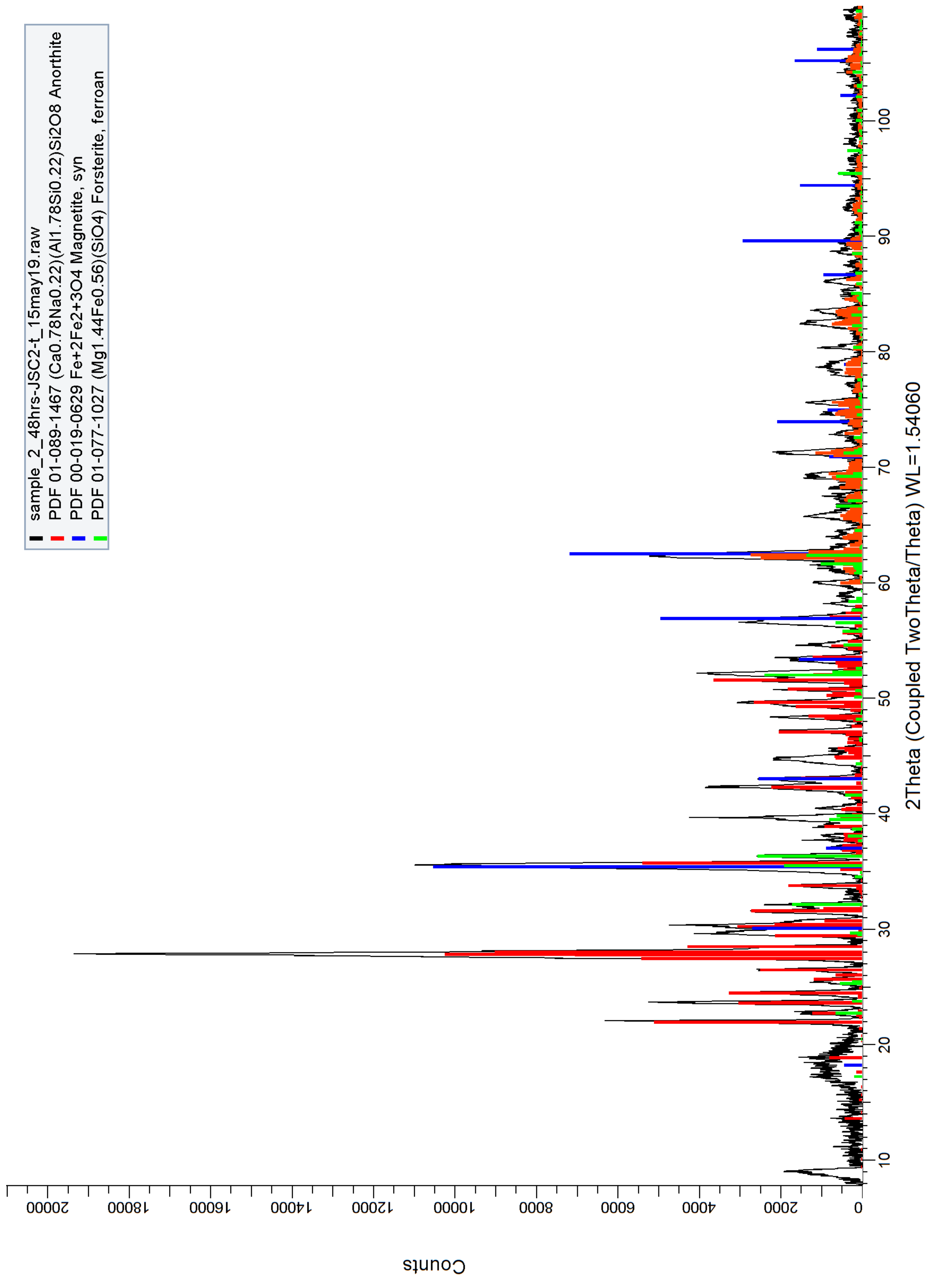

Figure 2 XRD pattern sample "2 48hrs-JSC2-t "

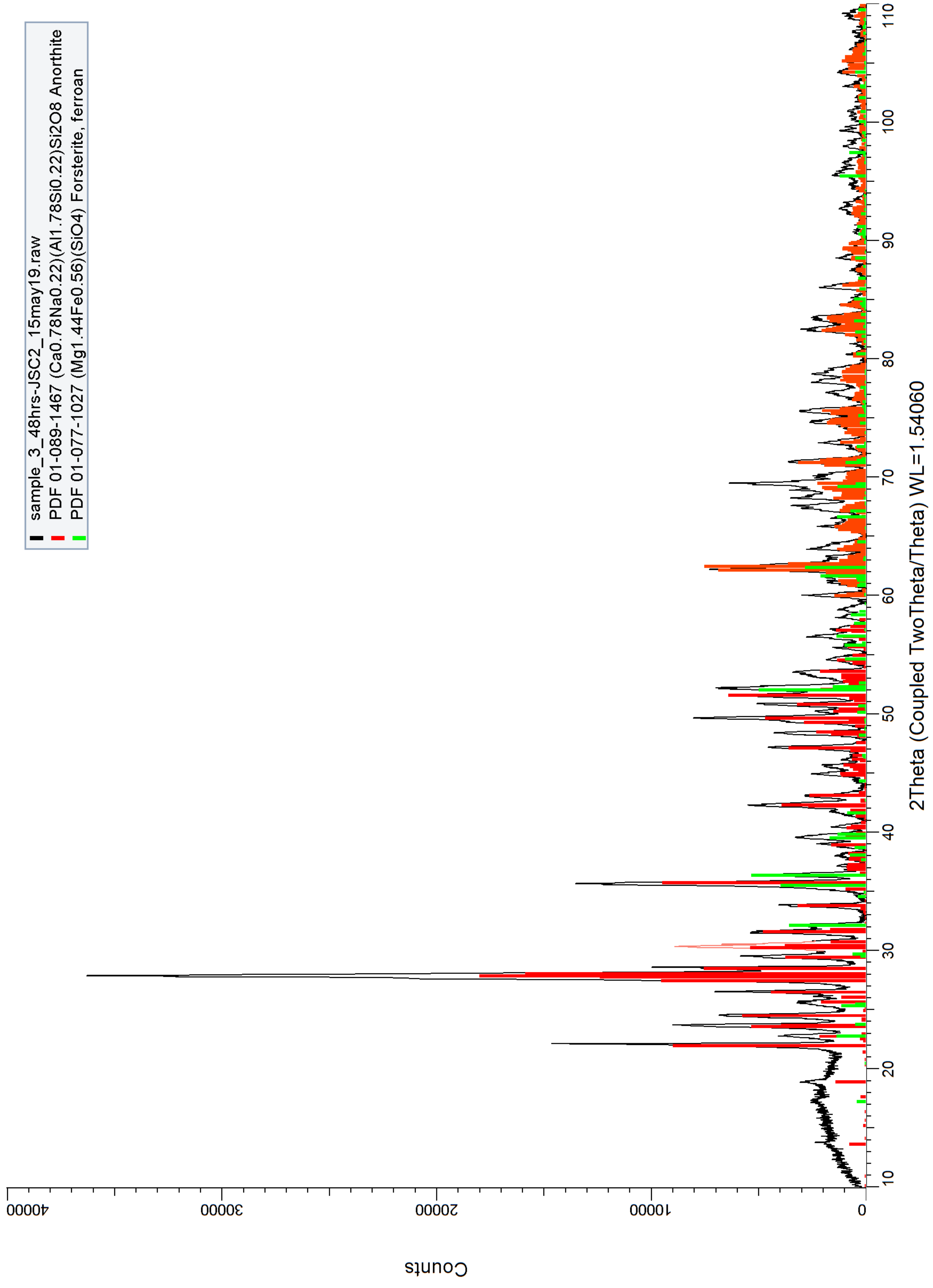

Figure 3 XRD pattern sample "3 48hrs-JSC2 "

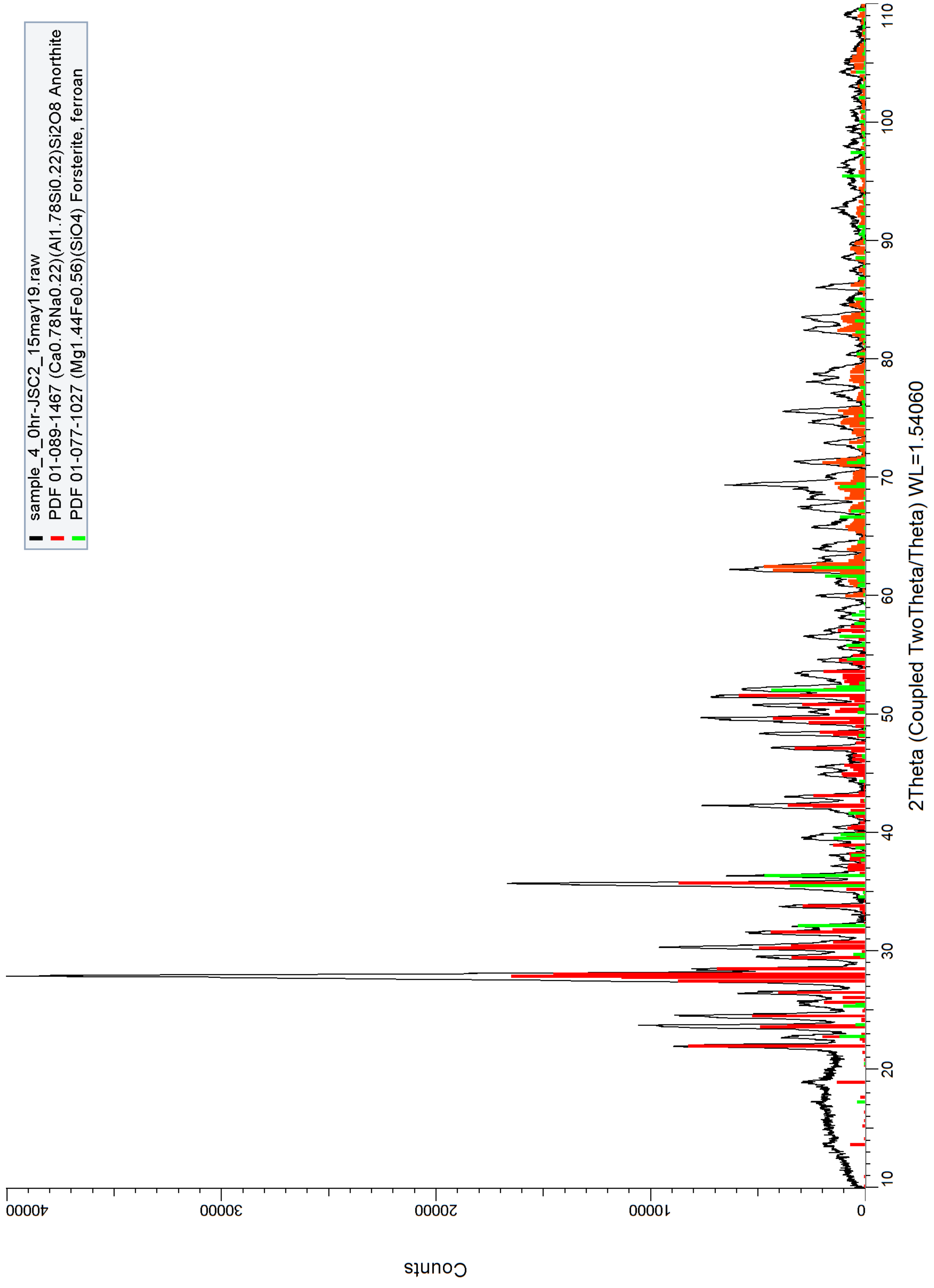

Figure 4 XRD pattern sample "4 0hr-JSC2 "

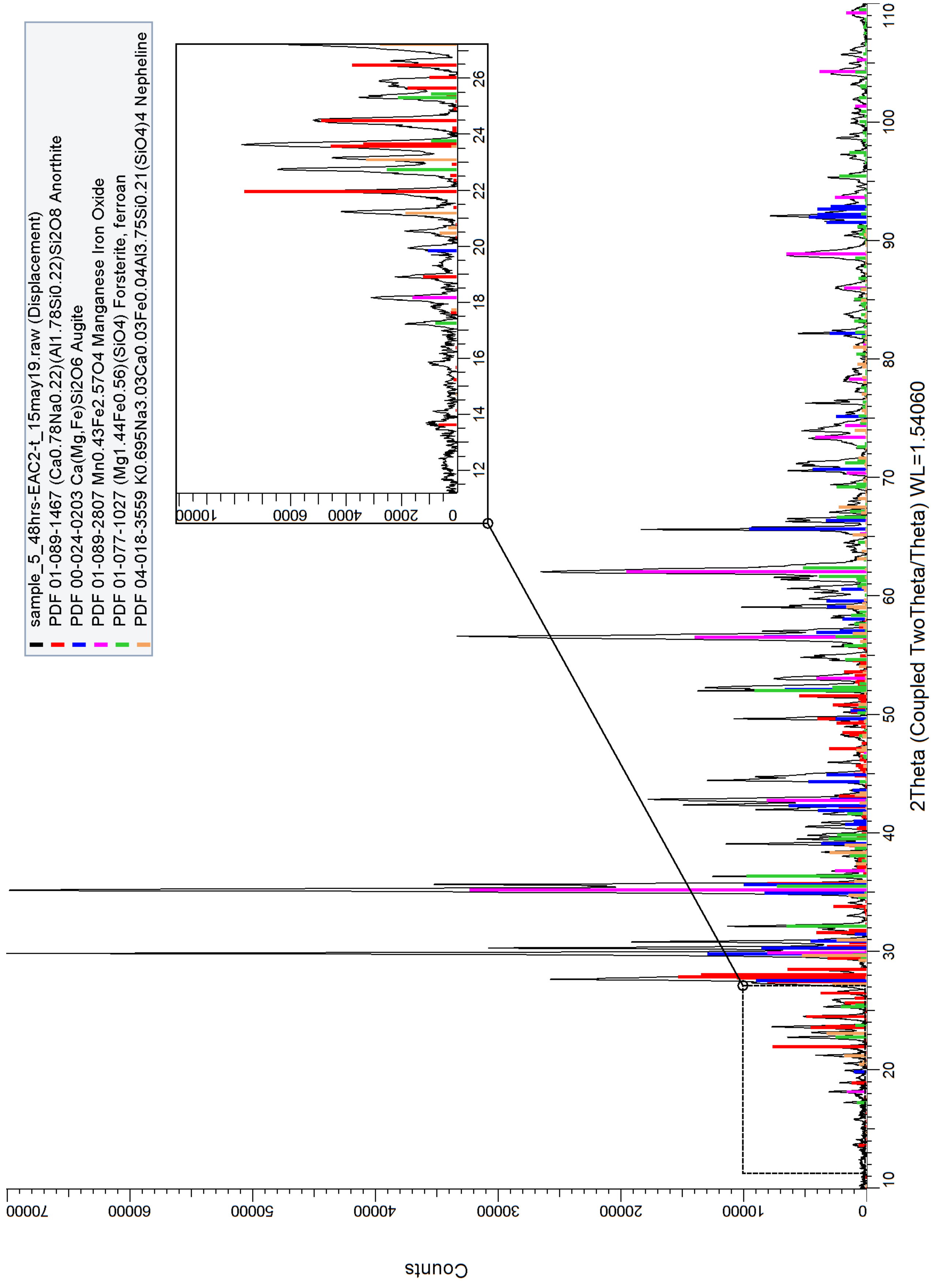

Figure 5 XRD pattern sample "5 48hrs-EAC2-t "

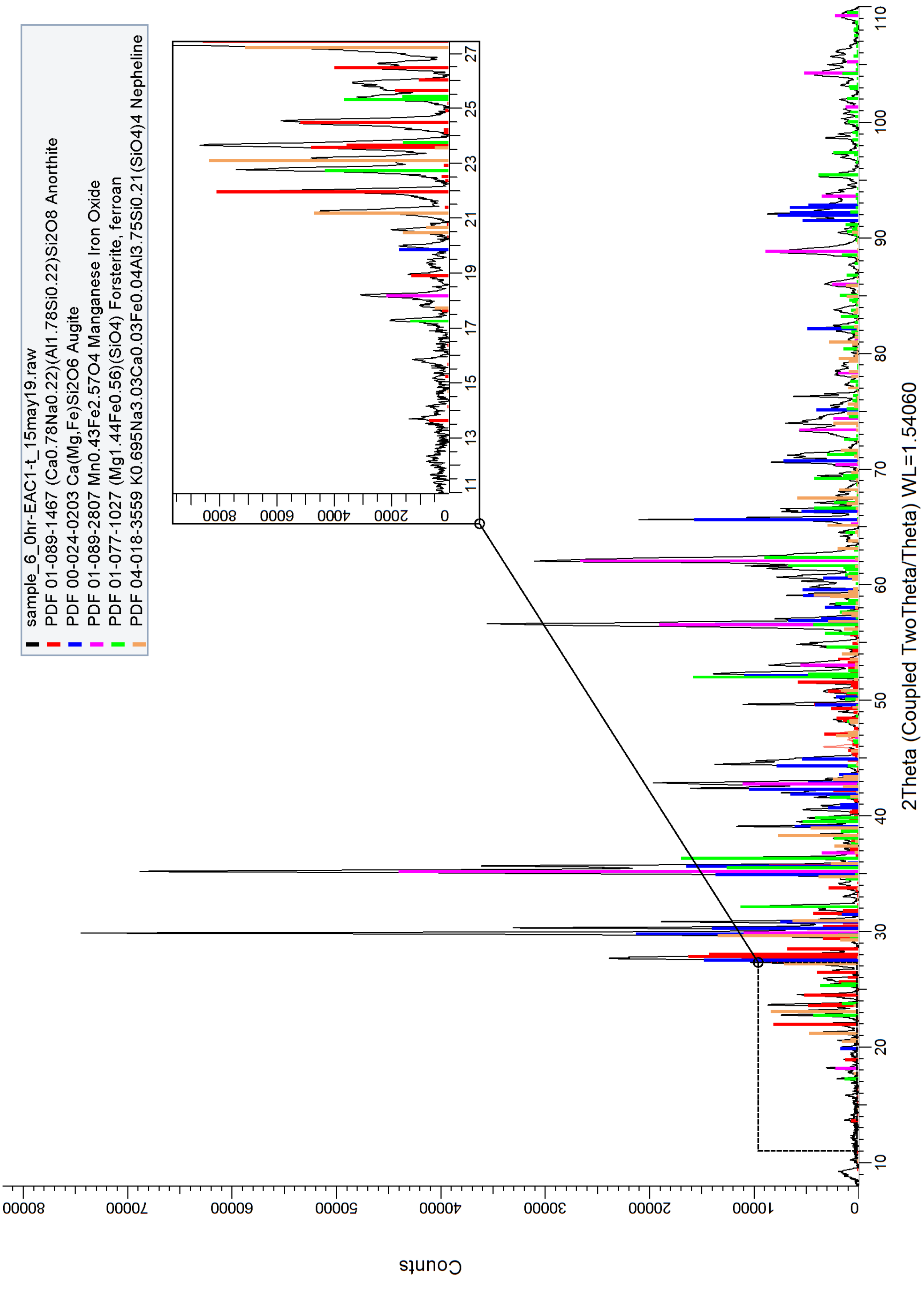

Figure 6 XRD pattern sample " 6 0hr-EAC1-t "
