# Supplementary material for: Iron can be microbially extracted from Lunar and Martian regolith simulants and 3D printed into tough structural materials
Source: PLoS One. 2021 Apr 28;16(4):e0249962. doi: 10.1371/journal.pone.0249962 (PMC8081250; doi:10.1371/journal.pone.0249962)
Supplement: S1 Data — (ZIP) [file pone.0249962.s001.zip › Data_updated/XRF,XRD/XRD_JSC2A_untreated_15apr19.pdf]

X-RAY FACILITIES GROUP

Dr. Amarante Böttger — *A.J.Bottger@tudelft.nl*
phone +31(0)1527·82243

Dhr. Ruud Hendrikx — *R.W.A.Hendrikx@tudelft.nl*
Drs. Richard Huizenga — *R.M.Huizenga@tudelft.nl*

Delft University of Technology, Faculty of 3mE
Department of Materials Science and Engineering
Mekelweg 2, NL-2628 CD Delft, the Netherlands, phone +31(0)1527·82255/89459

XRD measurements of lunar regolith simulants

Author	:	Ruud Hendrikx
Date	:	15 apr 19
Researcher	:	Benjamin Lehner. L&R
Research question	:	Phase identification

"For sustainable space exploration it is necessary to extract resource directly from the planet/moon we are going to. This process is called "in situ resource utilization (ISRU)". We developed a methodology to extract iron from different lunar regolith simulants using microbiology and magnetism".
See also page 6.

Samples
The samples are powders(<63 um) with label: "1_JSC1AF", "2_JC2A "and "3_LHT3M ".

Specimen
A small amount of powder was deposited as a thin layer on a Si510 wafer from a powder-isopropanol suspension.

Experimental
Instrument: Bruker D8 Advance diffractometer Bragg-Brentano geometry and Lynxeye position sensitive detector. Cu Kα radiation. Divergence slit V12, scatter screen height 5 mm, 45 kV 40 mA. Sample spinning. Detector settings LL 0.19 W 0.06. Sample holder L510.

Measurements
Coupled θ -2θ scan 8° - 110°, step size 0.021 ° 2θ, counting time per step 1 s.

Data evaluation
Bruker software DiffracSuite.EVA vs 5.0.

Results
Figures 1 -3 show the measured XRD patterns in black, between 10 and 60° 2θ, after background subtraction and for sample-3 a small displacement correction. The colored sticks give the peak positions and intensities of the possibly present phases, using the ICDD pdf4 database, see table 1.

sample	*compound*	
1_JSC1AF	Anorthite, ordered<br>Forsterite, ferroan	$CaAl_2Si_2O_8$<br>$Mg_{1.8}Fe_{0.2}SiO_4$
2_JC2A	Anorthite, ordered<br>Forsterite, ferroan	$CaAl_2Si_2O_8$<br>$Mg_{1.8}Fe_{0.2}SiO_4$
3_LHT3M	Anorthite, sodian<br>Enstatite, ferroan<br>Augite	$(Ca_{0.86}Na_{0.14})(Al_{1.84}Si_{2.16}O_8)$<br>$(Mg_{1.561}Fe_{0.439})Si_2O_6$<br>$Ca(Mg,Fe)Si_2O_6$

Table 1.

If the analysis is a significant part of a publication, a co-authorship is preferred.

In any case, it is useful to involve us in the preparation of any presentation to ensure optimum and correct use of the analysis results!

Whenever used in a publication, an acknowledgement will be appreciated, e.g.: "personX at the Department of Materials Science and Engineering of the Delft University of Technology is acknowledged for the X-ray analysis".

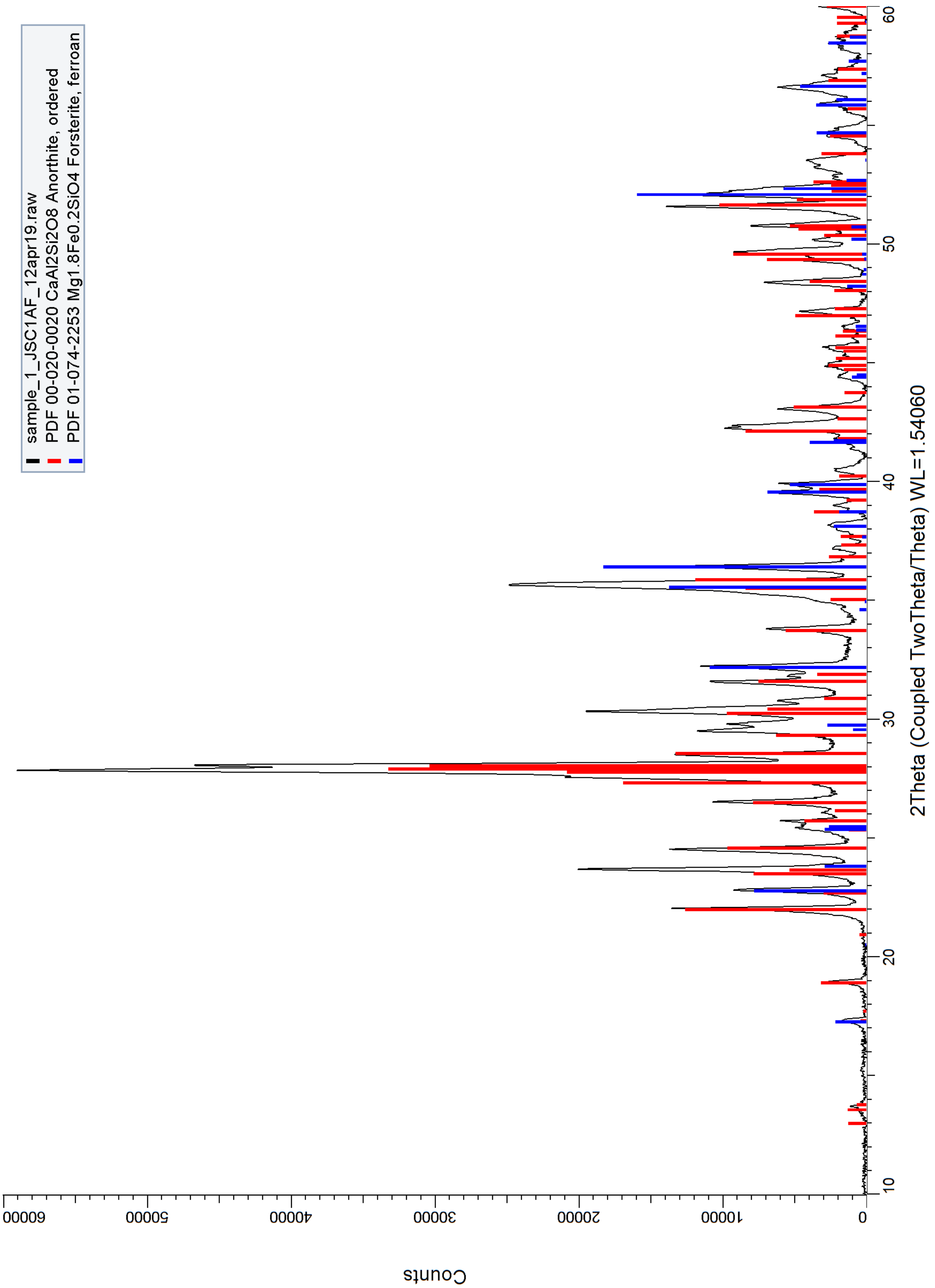

Figure 1 XRD pattern sample "1_JSC1AF "

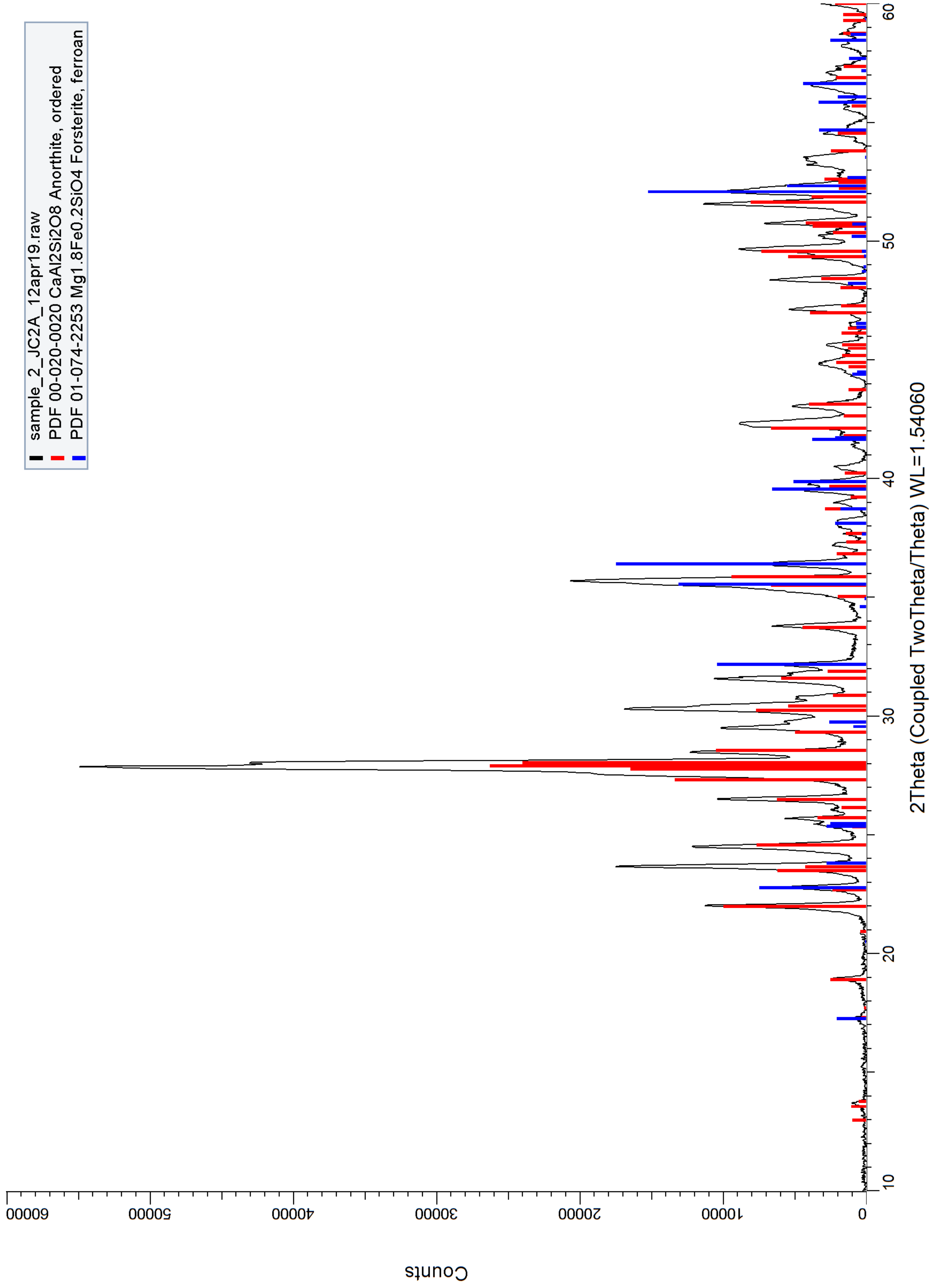

Figure 2 XRD pattern sample "2_JC2A "

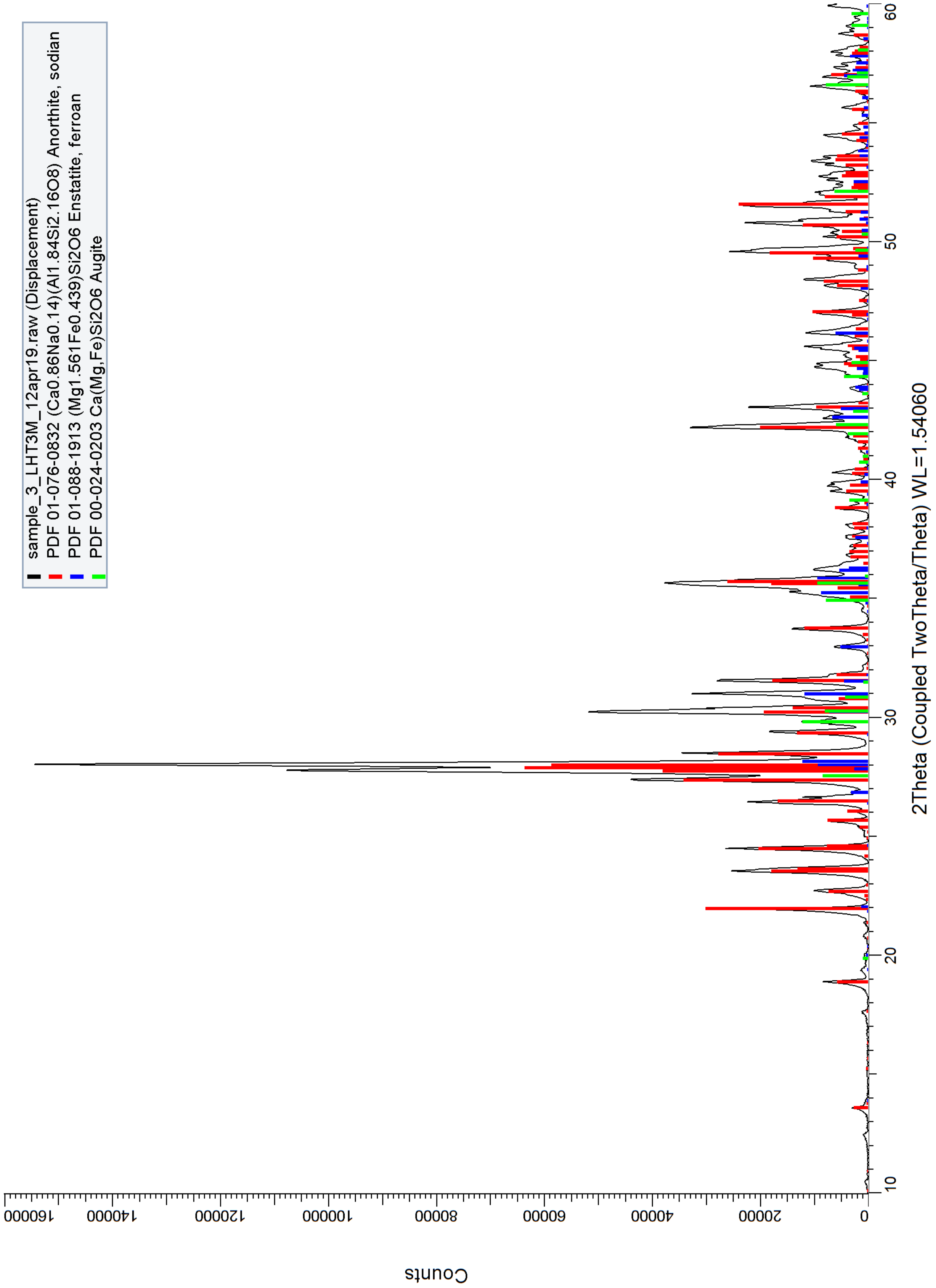

Figure 3 XRD pattern sample "3_LHT3M "

XRD and XRF measurements of microbial treated lunar regolith simulants

Short description:
For sustainable space exploration it is necessary to extract resource directly from the planet/moon we are going to. This process is called "*in situ* resource utilization (ISRU)". We developed a methodology to extract iron from different lunar regolith simulants using microbiology and magnetism (Fig.1). The extracted material is directly utilized via 3D printing approached done by our collaborators at ESA and NASA (+ some external companies). The 3D printed material is characterized regarding its conductivity, tensile strength and Young Module. We hope that the 3D printed material can be used for rocket replacement parts and a potential lunar habitat.
One critical factor is to understand the processes is to characterize the material before and after treatment (as well as the left-over material). We made a pilot study with people from Edinburgh University where we saw the expected increase in iron concentration for the magnetically active material, but further quantification is needed. This quantification of different elements could be achieved via XRF and XRD measurements and we would hope to collaborate with you on that (happy to give a co-authorship). And submit the work by July in the journal: "Astrobiology".

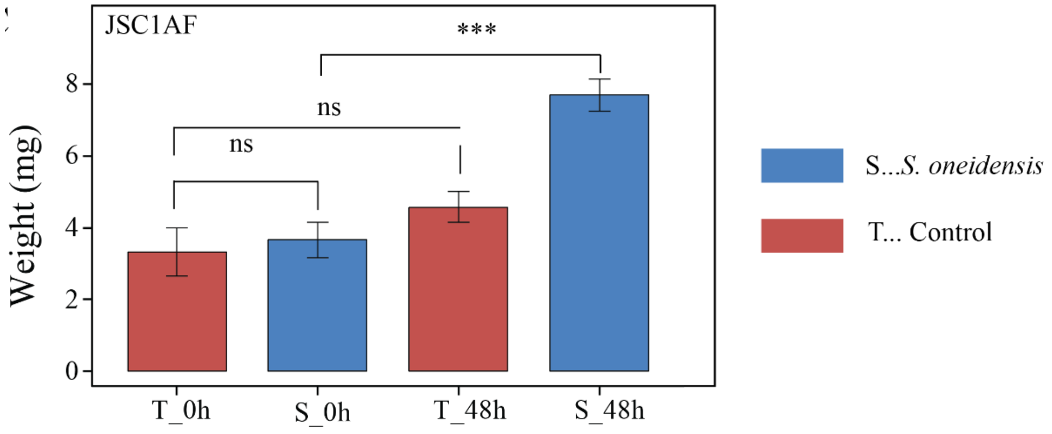

Fig.1 Improved methodology for magnetically-extracted material from lunar regolith simulant (JSC-1AF) using S. oneidensis. A significant difference was found between S. oneidensis at 0h (S_0h) and S. oneidensis at 48h (S_48h) ($p = 3.9e-5$) and between S. oneidensis at 48h (S_48h) and the TSB control at 48h (T_48h) ($p = 0.00058$). However, there were no differences between T_0h and T_48h ($p = 0.65$) or between S_0h and T_0h ($p = 0.99$).

Publication outline:
Submission beginning of July
- Fig 1 Toxicity of different lunar regolith simulants for the bacteria (done)
- Fig 2 Magnetic extraction from different lunar regolith simulants - weight (mainly done)
- Fig 3 XRD and XRF measurements of elemental compositions (Collaboration necessary)
- Fig 4 Utilization with 3D printing methods (ongoing)
