# Supplementary material for: Iron can be microbially extracted from Lunar and Martian regolith simulants and 3D printed into tough structural materials
Source: PLoS One. 2021 Apr 28;16(4):e0249962. doi: 10.1371/journal.pone.0249962 (PMC8081250; doi:10.1371/journal.pone.0249962)
Supplement: S1 Data — (ZIP) [file pone.0249962.s001.zip › Data_updated/XRF,XRD/XRF_EAC1_JSC2A_JSCMars1_untreated_and_treated_20may19.pdf]

Materials Science and Engineering
TUDelft, Faculty of 3mE
Mekelweg 2
2628CD Delft, The Netherlands
Tel. 015 27 82244/89459
Email: R.W.A. Hendrikx@tudelft.nl
X- ray diffraction facilities

<u>Experimental conditions:</u>

For XRF analysis the measurements were performed with a Panalytical Axios Max WD-XRF spectrometer and data evaluation was done with SuperQ5.0i/Omnian software. 18/12/2015 09:37:03

20/05/2019 13:07:08

PANalytical

Quantification of sample B. Lehner, sample "EAC1 untreated", 13may19

Sum before normalization: 43.7 wt%

Normalised to: 100.0 wt%

Sample type: Pressed powder

Initial sample weight (g): 2.000

Weight after pressing (g): 2.500

Oxygen validation factor: 0.00

Correction applied for medium: No

Correction applied for film: No

Used Compound list: Oxides

Results database: omnian 4kw 27mm

Results database in: c:\panalytical\superq\userdata

	Compound Name	Conc. (wt%)	Absolute Error (wt%)
1	MnO	78.6	1
2	SiO2	7.211	0.08
3	Fe2O3	4.325	0.06
4	CaO	4.144	0.06
5	Al2O3	1.835	0.04
6	TiO2	1.46	0.04
7	CuO	1.022	0.04
8	SrO	0.47	0.02
9	K2O	0.162	0.01
10	MgO	0.135	0.01
11	NiO	0.128	0.01
12	BaO	0.124	0.01
13	ZnO	0.119	0.01
14	ZrO2	0.107	0.01
15	Cr2O3	0.091	0.009
16	Nb2O5	0.043	0.006
17	Rb2O	0.024	0.005

20/05/2019 13:11:39

PANalytical

Quantification of sample B. Lehner, sample "0hrs EAC1 treated", 13may19

Sum before normalization: 102.2 wt%

Normalised to: 100.0 wt%

Sample type: Pressed powder

Initial sample weight (g): 2.000

Weight after pressing (g): 2.500

Used Compound list: Oxides

Results database: omnian 4kw 27mm

Results database in: c:\panalytical\superq\userdata

	Compound Name	Conc. (wt%)	Absolute Error (wt%)
1	SiO2	35.491	0.1
2	Fe2O3	22.984	0.1
3	Al2O3	13.523	0.1
4	MgO	9.806	0.09
5	CaO	7.622	0.08
6	TiO2	6.124	0.07
7	Na2O	1.208	0.03
8	K2O	1.057	0.03
9	P2O5	0.855	0.03
10	Cr2O3	0.469	0.02
11	CuO	0.266	0.02
12	NiO	0.174	0.01
13	V2O5	0.081	0.009
14	Cl	0.079	0.008
15	SO3	0.059	0.007
16	SrO	0.054	0.007
17	CeO2	0.052	0.007
18	ZnO	0.032	0.005
19	Nb2O5	0.016	0.004
20	ZrO2	0.015	0.004
21	BaO	0.013	0.003
22	MoO3	0.006	0.002
23	Rb2O	0.003	0.002
24	Y2O3	0.002	0.001

20/05/2019 13:13:26

PANalytical

Quantification of sample B. Lehner, sample "48hrs EAC1 treated", 13may19

Sum before normalization: 94.9 wt%

Normalised to: 100.0 wt%

Sample type: Pressed powder

Initial sample weight (g): 2.000

Weight after pressing (g): 2.500

Used Compound list: Oxides

Results database: omnian 4kw 27mm

Results database in: c:\panalytical\superq\userdata

	Compound Name	Conc. (wt%)	Absolute Error (wt%)
1	SiO2	39.492	0.1
2	Fe2O3	23.769	0.1
3	Al2O3	14.692	0.1
4	CaO	8.89	0.09
5	TiO2	6.07	0.07
6	MgO	2.06	0.04
7	K2O	1.147	0.03
8	Co3O4	0.908	0.05
9	P2O5	0.905	0.03
10	Na2O	0.766	0.03
11	Cr2O3	0.402	0.02
12	CuO	0.21	0.01
13	NiO	0.208	0.01
14	Cl	0.096	0.009
15	V2O5	0.085	0.009
16	SrO	0.067	0.008
17	SO3	0.064	0.008
18	CeO2	0.052	0.007
19	ZrO2	0.036	0.006
20	ZnO	0.032	0.005
21	BaO	0.019	0.004
22	Nb2O5	0.015	0.004
23	MoO3	0.004	0.002
24	Rb2O	0.003	0.002

20/05/2019 13:28:25

PANalytical

Quantification of sample B. Lehner, sample "0hrs SSC2 um", 13may19

Sum before normalization: 85.2 wt%

Normalised to: 100.0 wt%

Sample type: Pressed powder

Initial sample weight (g): 2.000

Weight after pressing (g): 2.500

Used Compound list: Oxides

Results database: omnian 4kw 27mm

Results database in: c:\panalytical\superq\userdata

	Compound Name	Conc. (wt%)	Absolute Error (wt%)
1	SiO2	46.552	0.1
2	Al2O3	20.922	0.1
3	Fe2O3	14.156	0.1
4	CaO	11.736	0.1
5	TiO2	2.57	0.05
6	P2O5	1.071	0.03
7	K2O	0.903	0.03
8	MgO	0.707	0.03
9	CuO	0.356	0.02
10	Na2O	0.291	0.02
11	MnO	0.193	0.01
12	SrO	0.153	0.01
13	Cl	0.09	0.009
14	CeO2	0.071	0.008
15	SO3	0.044	0.006
16	BaO	0.043	0.006
17	V2O5	0.043	0.006
18	ZnO	0.026	0.005
19	ZrO2	0.022	0.004
20	NiO	0.021	0.004
21	Cr2O3	0.02	0.004
22	Nb2O5	0.008	0.003
23	Y2O3	0.003	0.002

20/05/2019 13:29:55

PANalytical

Quantification of sample B. Lehner, sample "48hrs SSC2 um", 13may19

Sum before normalization: 80.8 wt%

Normalised to: 100.0 wt%

Sample type: Pressed powder

Initial sample weight (g): 2.000

Weight after pressing (g): 2.500

Used Compound list: Oxides

Results database: omnian 4kw 27mm

Results database in: c:\panalytical\superq\userdata

	Compound Name	Conc. (wt%)	Absolute Error (wt%)
1	SiO2	45.52	0.1
2	Al2O3	20.518	0.1
3	Fe2O3	14.757	0.1
4	CaO	11.941	0.1
5	TiO2	2.536	0.05
6	P2O5	1.309	0.03
7	MgO	1.017	0.03
8	K2O	0.939	0.03
9	CuO	0.355	0.02
10	Na2O	0.259	0.02
11	MnO	0.208	0.01
12	Cl	0.188	0.01
13	SrO	0.162	0.01
14	V2O5	0.06	0.007
15	ZrO2	0.052	0.007
16	BaO	0.052	0.007
17	SO3	0.041	0.006
18	Cr2O3	0.033	0.005
19	ZnO	0.023	0.005
20	NiO	0.018	0.004
21	Nb2O5	0.008	0.003
22	Y2O3	0.004	0.002

20/05/2019 13:31:20

PANalytical

Quantification of sample B. Lehner, sample "Mars JSC1 untreated", 13may19

Sum before normalization: 86.2 wt%

Normalised to: 100.0 wt%

Sample type: Pressed powder

Initial sample weight (g): 2.000

Weight after pressing (g): 2.500

Used Compound list: Oxides

Results database: omnian 4kw 27mm

Results database in: c:\panalytical\superq\userdata

	Compound Name	Conc. (wt%)	Absolute Error (wt%)
1	SiO2	37.474	0.1
2	Al2O3	28.579	0.1
3	Fe2O3	20.448	0.1
4	CaO	5.51	0.07
5	TiO2	4.827	0.06
6	P2O5	1.294	0.03
7	MnO	0.354	0.02
8	K2O	0.333	0.02
9	CuO	0.258	0.02
10	SO3	0.236	0.01
11	ZnO	0.234	0.01
12	SrO	0.125	0.01
13	ZrO2	0.09	0.009
14	MgO	0.083	0.009
15	CeO2	0.063	0.007
16	Cl	0.027	0.005
17	BaO	0.02	0.004
18	NiO	0.016	0.004
19	Cr2O3	0.013	0.003
20	Nb2O5	0.01	0.003
21	Y2O3	0.007	0.003

20/05/2019 13:33:20

PANalytical

Quantification of sample B. Lehner, sample "48hrs JSC2 treated", 13may19

Sum before normalization: 72.4 wt%

Normalised to: 100.0 wt%

Sample type: Pressed powder

Initial sample weight (g): 2.000

Weight after pressing (g): 2.500

Used Compound list: Oxides

Results database: omnian 4kw 27mm

Results database in: c:\panalytical\superq\userdata

	Compound Name	Conc. (wt%)	Absolute Error (wt%)
1	SiO2	43.227	0.1
2	Fe2O3	21.085	0.1
3	Al2O3	17.499	0.1
4	CaO	11.243	0.09
5	TiO2	3.295	0.05
6	K2O	1.003	0.03
7	P2O5	0.94	0.03
8	CuO	0.373	0.02
9	MnO	0.291	0.02
10	ZnO	0.238	0.01
11	SrO	0.152	0.01
12	Cr2O3	0.135	0.01
13	CeO2	0.109	0.01
14	NiO	0.069	0.008
15	BaO	0.061	0.007
16	V2O5	0.059	0.007
17	SO3	0.057	0.007
18	MgO	0.051	0.007
19	Cl	0.049	0.007
20	ZrO2	0.03	0.005
21	MoO3	0.017	0.004
22	Nb2O5	0.013	0.003
23	Y2O3	0.006	0.002

Use of our XRD or XRF analysis:
In a publication: 'PersonX at the Department of Materials Science and Engineering of the Delft University of Technology is acknowledged for the X-ray analysis. If it is an important part of the publication: a co-authorship is preferred. It is useful to involve us in the preparation of any presentation!
