# Supplementary material for: Iron can be microbially extracted from Lunar and Martian regolith simulants and 3D printed into tough structural materials
Source: PLoS One. 2021 Apr 28;16(4):e0249962. doi: 10.1371/journal.pone.0249962 (PMC8081250; doi:10.1371/journal.pone.0249962)
Supplement: S1 Data — (ZIP) [file pone.0249962.s001.zip › Data_updated/XRF,XRD/XRF_JSC2A_untreated_12apr19.pdf]

Materials Science and Engineering
TUDelft, Faculty of 3mE
Mekelweg 2
2628CD Delft, The Netherlands
Tel. 015 27 82244/89459
Email: R.W.A. Hendrikx@tudelft.nl
X- ray diffraction facilities

Experimental conditions:

For XRF analysis the measurements were performed with a Panalytical Axios Max WD-XRF spectrometer and data evaluation was done with SuperQ5.0i/Omnian software. 18/12/2015 09:37:03

12/04/2019 16:23:05

PANalytical

Quantification of sample B. Lehner, sample 1 "JSC-1AF", 12apr19

Sum before normalization: 98.6 wt%

Normalised to: 100.0 wt%

Sample type: Pressed powder

Initial sample weight (g): 2.000

Weight after pressing (g): 2.500

Used Compound list: Oxides

Results database: omnian 4kw 27mm

Results database in: c:\panalytical\superq\userdata

	Compound Name	Conc. (wt%)	Absolute Error (wt%)
1	SiO_2	44.177	0.1
2	Al_2O_3	19.058	0.1
3	Fe_2O_3	12.754	0.1
4	CaO	10.208	0.09
5	MgO	6.165	0.07
6	Na_2O	3.548	0.06
7	TiO_2	2.195	0.04
8	P_2O_5	0.677	0.02
9	K_2O	0.657	0.02
10	MnO	0.177	0.01
11	SrO	0.103	0.01
12	BaO	0.053	0.007
13	V_2O_5	0.052	0.007
14	Cl	0.04	0.006
15	ZrO_2	0.036	0.006
16	SO_3	0.03	0.005
17	Cr_2O_3	0.02	0.004
18	ZnO	0.015	0.004
19	CuO	0.012	0.003
20	NiO	0.012	0.003
21	Nb_2O_5	0.005	0.002
22	Y_2O_3	0.003	0.002

12/04/2019 16:25:02

PANalytical

Quantification of sample B. Lehner, sample 2 "JC2A", 12apr19

Sum before normalization: 97.8 wt%

Normalised to: 100.0 wt%

Sample type: Pressed powder

Initial sample weight (g): 2.000

Weight after pressing (g): 2.500

Used Compound list: Oxides

Results database: omnian 4kw 27mm

Results database in: c:\panalytical\superq\userdata

	Compound Name	Conc. (wt%)	Absolute Error (wt%)
1	SiO2	44.894	0.1
2	Al2O3	19.616	0.1
3	Fe2O3	12.779	0.1
4	CaO	10.1	0.09
5	MgO	4.721	0.06
6	Na2O	3.676	0.06
7	TiO2	2.23	0.04
8	P2O5	0.682	0.02
9	K2O	0.671	0.02
10	MnO	0.164	0.01
11	SrO	0.109	0.01
12	CuO	0.109	0.01
13	Cr2O3	0.048	0.007
14	Cl	0.04	0.006
15	BaO	0.037	0.006
16	SO3	0.037	0.006
17	V2O5	0.027	0.005
18	NiO	0.021	0.004
19	ZnO	0.017	0.004
20	ZrO2	0.014	0.004
21	Nb2O5	0.005	0.002
22	MoO3	0.003	0.002
23	Y2O3	0.003	0.002

12/04/2019 16:26:21

PANalytical

Quantification of sample B. Lehner, sample 3 "LHT3M", 12apr19

Sum before normalization: 84.6 wt%

Normalised to: 100.0 wt%

Sample type: Pressed powder

Initial sample weight (g): 2.000

Weight after pressing (g): 2.500

Used Compound list: Oxides

Results database: omnian 4kw 27mm

Results database in: c:\panalytical\superq\userdata

	Compound Name	Conc. (wt%)	Absolute Error (wt%)
1	SiO2	46.237	0.1
2	Al2O3	25.409	0.1
3	CaO	12.568	0.1
4	MgO	8.312	0.08
5	Fe2O3	5.7	0.07
6	Na2O	1.121	0.03
7	Cr2O3	0.136	0.01
8	TiO2	0.131	0.01
9	K2O	0.087	0.009
10	MnO	0.082	0.009
11	NiO	0.049	0.007
12	Cl	0.029	0.005
13	ZrO2	0.028	0.005
14	CeO2	0.026	0.005
15	P2O5	0.026	0.005
16	SO3	0.019	0.004
17	SrO	0.013	0.003
18	CuO	0.012	0.003
19	ZnO	0.011	0.003
20	MoO3	0.004	0.002

Use of our XRD or XRF analysis:
In a publication: 'PersonX at the Department of Materials Science and Engineering of the Delft University of Technology is acknowledged for the X-ray analysis. If it is an important part of the publication: a co-authorship is preferred. It is useful to involve us in the preparation of any presentation!
