# Supplementary material for: Iron can be microbially extracted from Lunar and Martian regolith simulants and 3D printed into tough structural materials
Source: PLoS One. 2021 Apr 28;16(4):e0249962. doi: 10.1371/journal.pone.0249962 (PMC8081250; doi:10.1371/journal.pone.0249962)
Supplement: S1 Data — (ZIP) [file pone.0249962.s001.zip › Data_updated/Strenght test/Report RS0004277_v4_Final.pdf]

estec

European Space Research
and Technology Centre
Keplerlaan 1
2201 AZ Noordwijk
The Netherlands
T +31 (0)71 565 6565
F +31 (0)71 565 6040
www.esa.int

DOCUMENT

TEST REPORT

RS0004277 - Lithoz – Compression Testing of Lunar Regolith Simulant JSC-2A specimens

RS0004277

Please complete the customer satisfaction survey at:
https://tecsurveys.esa.int/tecq

This document may not be reproduced, except in full, without the written permission of the European Space Agency.
Uncontrolled when printed.

**Prepared by**	**Maude Maréchal**
**Reference**	**ESA-TECMSP-TR-021220**
**Laboratory**	**ESA Materials and Electrical Components Laboratory**
**Area**	**TEC-MSP**
**Issue**	**1**
**Revision**	**1**
**Date of Issue**	**03/11/2020**
**Document Type**	**TR**

European Space Agency
Agence spatiale européenne

APPROVAL

RS0004277 - Lithoz – Compression Testing of Lunar Regolith Simulant JSC-2A specimens	
**Issue 1**	**Revision 1**
**Author Maude Maréchal (TEC-MSP)**	**Date: 03-Nov-2020**
**Reviewed by**	**Date**
**Advenit Makaya(TEC-MSP)**	**03-Nov-2020** Advenit Makaya Digitally signed by Advenit Makaya Date: 2021.02.28 19:55:32 +01'00'
**Approved by**	**Date**
**Thomas Rohr, Line Manager**	

Please refer to document database for electronic approval evidence.

CHANGE LOG

Reason for change	Issue	Revision	Date
Initial Release	1	1	28/02/2021

Please refer to document database for electronic version history.

CHANGE RECORD

Issue 1	Revision 1		
**Reason for change**	**Date**	**Pages**	**Paragraph(s)**

DISTRIBUTION LIST

This document may not be reproduced, except in full, without the written permission of the European Space Agency.

Table of contents:

EXECUTIVE SUMMARY 4
1 INTRODUCTION 5
2 REFERENCE DOCUMENTS 5
3 SAMPLE(S) 5
3.1 Description / Basic Information 5
4 EXPERIMENTAL 5
4.1 Test and Test Method 5
5 RESULTS 6
5.1 CT 6
5.2 Compression CT 8
5.3 Compression testing 7
6 ANALYSIS AND DISCUSSION 8
7 CONCLUSIONS AND RECOMMENDATIONS 10
8 BIBLIOGRAPHY 11
9 APPENDICES 11

This document may not be reproduced, except in full, without the written permission of the European Space Agency.

EXECUTIVE SUMMARY

Lithoz GmbH produced cylinders in lunar regolith simulant JSC-2A via Lithography-based Ceramic Manufacturing (additive manufacturing). It was decided that ESA would perform compressive testing and Computer Tomography (CT) scanning, to support a future joint publication between the Technische Universiteit Delft (TU Delft), Lithoz and ESA. CT scans were performed on 2 specimens, and compression tests on 10 specimens. On top of that, some Compression-CT testing (i.e. compression testing during CT scanning) were performed on 2 specimens.

Two types of porosity were observed: standard spherical pores and flat-disc pores – indicating delaminations between the additive manufacturing layers. On both of the specimens which underwent CT scans, some conical-shaped defect zones were found on the extremities, highlighting a probable issue during the additive manufacturing process.

The Ultimate Compressive Strength was measured to be 3.33 ± 0.39 [MPa], and the Elasticity Modulus 373.44 ± 77.60 [MPa]. This is lower than results obtained with the same process but with lunar regolith simulant EAC-1 (Ultimate Compressive Strength of 5.41 ± 0.29 [MPa], and a Modulus of 403.34 ± 45.10 [MPa]).

Compression-CT testing revealed that the defects originally present in the sample had almost no effect on the crack initiation and propagation. On the other hand, the non-planar and non-parallel top and down surfaces, adding up to the cone-shape defects found next to them, could have played a major role in the failure.

This document may not be reproduced, except in full, without the written permission of the European Space Agency.

1 INTRODUCTION

Lithoz GmbH produced cylinders in a lunar regolith simulant JSC-2A via Lithography-based Ceramic Manufacturing (additive manufacturing), as reported in RD01. ESA and Lithoz decided to prepare a joint publication on that study. To support the preparation of the publication, it was decided that ESA would perform the compressive testing following the DIN 51104 standard. Computerized Tomography (CT) was also used to check the porosity of the samples. On top of that, Compression-CT was used to observe the crack formation and propagation.

2 REFERENCE DOCUMENTS

No.	Report Version	Document title
RD01	01-2018-08-22	Feasibility Study – Report FS_20180605
RD02	17-01-2020	Lithoz – Regolith Compression Testing – RS0003855

3 SAMPLE(S)

3.1 Description / Basic Information

Twelve cylindrical samples, sintered at 1050°C, were received at ESTEC. Samples geometry was designed based on the DIN 51104 standard. The height of the specimens was measured to be 8.24 mm ± 0.04 mm. Their diameter was measured to be 4.71mm ± 0.01 mm. This is slightly higher than the standard's tolerances (Diameter of 4.5mm ± 0.1mm / Height of 8.0mm ± 0.2mm). Figure 2 shows as received specimens, in top and side view.

4 EXPERIMENTAL

4.1 Test and Test Method

In order to evaluate the quality of the parts, CT scans were performed on two different specimens (respectively #3 and #4). This was made with a Phoenix V|tome|x m 300 kV from General Electric. Compression testing during CT scanning (later referred to as Compression-CT) was performed on samples #1 and #2. This test was conducted in the Phoenix V|tome|x m 300 kV CT equipment, together with a Deben Microtest Module (CT5000 5kN in-situ tensile/compression stage). A scanning voltage of 80kV and a current of 70µA were used.
Compressive tests were performed with an Instron ElectroPuls E10000 Linear-Torsion, on samples #3 to #12. Room conditions: 22°C, about 50%rel. humidity. The DIN 51104 standard was followed, expect for two deviations:

1. No intermediate plate was used between the machine and the specimens. This was considered not needed, as the parts were known to be quite brittle and unlikely to damage the machine.

This document may not be reproduced, except in full, without the written permission of the European Space Agency.

2. The samples were not fully compliant to the standard, as their end-surfaces were not perfectly parallel. This was considered to affect the initial part of the stress-strain curve, but to have a relatively limited impact on the determination of the Ultimate Compressive Strength and the Elasticity Modulus (provided that the modulus calculation does not account for the initial part of the curve).

A constant deformation rate of 0.5mm/min was used. The Ultimate Compression Strength was calculated by considering the maximum load the sample withstood and its initial diameter. Generally, the Modulus was calculated by taking the slope of a linear regression line, itself determined by considering the data between 1 MPa and 2.5MPa. For samples #6, #7 and #11, the Modulus was calculated between 1MPa and 2MPa, due to the slight difference in their curves.

For the Compression-CT, the microtest compression setup was placed in the CT machine. The deformation rate was also set at 0.5mm/min. The moving compression plate was initially put in contact with the sample by applying a 3N preload (deformation rate of 0.5mm/min, 0.2MPa). The first desired load level was then applied and maintained, and a CT scan was run. Each scan was between 30 minutes and 1h long. The next load level was then applied, and a new scan run. This was continued until full failure of the sample. Due to the presence of the setup around the samples, the top and bottom parts of each sample were not possible to scan.

5 RESULTS

5.1 CT

Typical sections of specimens #1 and #3 CT scans are presented in Figures 3, 4 and 5. The deposited layers, inherent to the additive manufacturing process were clearly visible, particularly in the central part of the specimens, as evidenced in Figure 3. Two different types of porosity were found in the sample. The first type consisted of almost spherical porosities, found homogeneously in the sample. Such pores were relatively small, typically around 65 µm in diameter. The other kind of porosity presented a flat-disc morphology, lying parallel to the build plan (XY plan). Those discs had a relatively high diameter, but very limited height. An example is shown in Figure 6. Its dimensions in the XY plan were 188 µm and 306 µm, whereas it was only 14 µmn high in the Z direction (parallel to build direction). The biggest observed flat disc pore is shown in Figure 7. As indicated, its dimensions in the plan were comprised between 450 µm and 500 µm.
A relatively large area of defects was found at the base of sample #3, as seen in Figures 8 and 9. This was characterised by a large amount of porosity. This defective zone was found to have a conical shape, as shown in Figure 9.
Unfortunately, adequate calibration could not be achieved during the CT scan of sample #4 and, as a result, most of the porosities could not be detected clearly. Nevertheless, the cone-shape defect zone found in specimen #3 was also found in specimen #4, as shown in Figure 10.

This document may not be reproduced, except in full, without the written permission of the European Space Agency.

5.2 Compression testing

Table 1 displays the calculated Elasticity Modulus and the measured Ultimate Compressive Strength for each specimen tested. The average Elasticity Modulus was found to be 373.44 ± 77.60 [MPa], and the average Ultimate Compressive Strength 3.33 ± 0.39 [MPa].
The stress-strain curves for compression testing were globally similar for all the samples, as shown in Figure 1, expect for sample 7. This sample presented a double peak, which probably indicates significant defects in the sample. This sample was thus excluded from the calculations of the average Elasticity Modulus and Ultimate Compressive Strength.

Table 1: Modulus and Ultimate compressive strength overview

Sample	**Elasticity Modulus [MPa]**	**Ultimate Compressive Strength [MPa]**
3	388.30	3.29
4	417.14	3.99
5	459.10	3.41
6	272.15	2.69
7	*(167.94)*	*(2.28)*
8	487.66	3.59
9	388.11	3.15
10	373.58	3.42
11	301.54	2.89
12	273.36	3.53
Mean	**373.44**	**3.33**
STD	**77.60**	**0.39**

This document may not be reproduced, except in full, without the written permission of the European Space Agency.

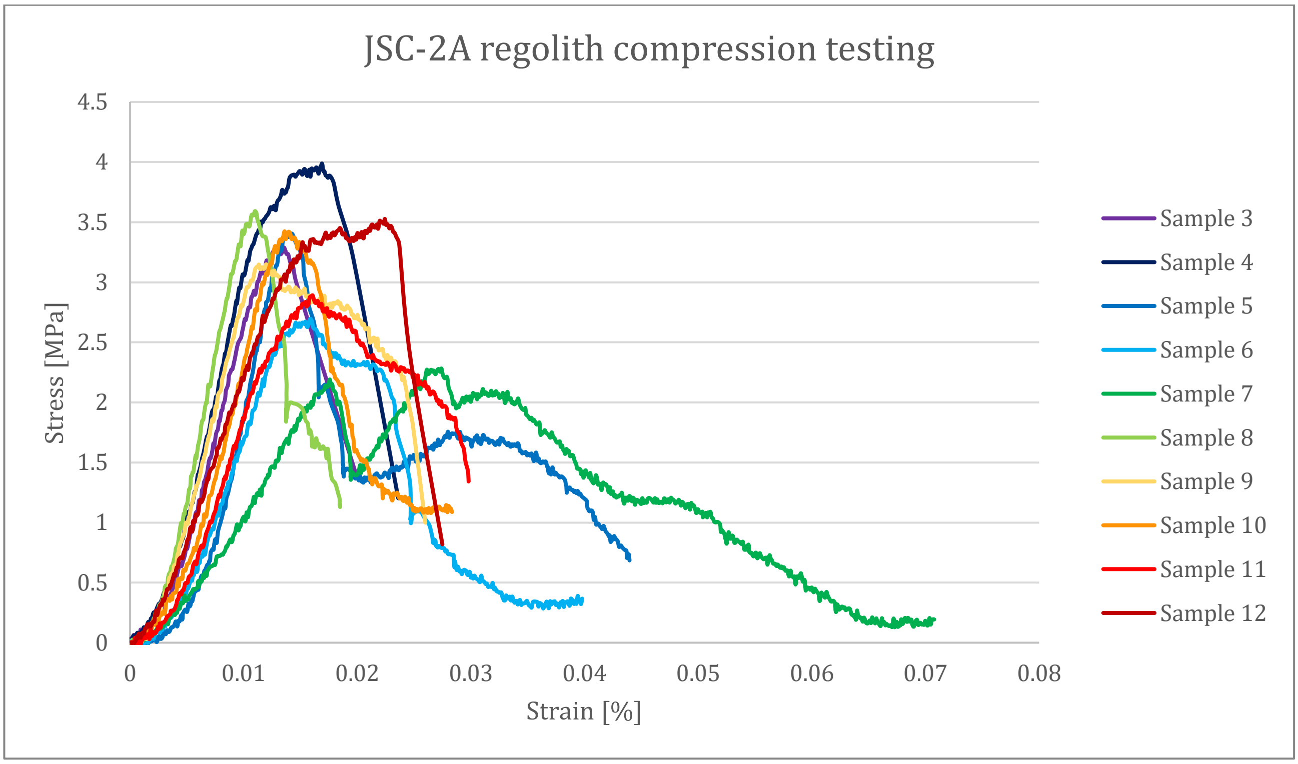

Figure 1: Stress-strain curves of compression tested specimen

5.3 Compression-CT

Considering the standard compression results, it was initially decided to test the samples at 1MPa, 2MPa, 3MPa, and 3.5 MPa, unless they would break before reaching those values.
The initial crack in sample #1 appeared at its bottom, and was seen on the scan done at 1MPa. The specimen then broke when reaching 2MPa. The compression test setup was then stopped in the broken position, in order to do the CT-scan. An evolution of the cracks observed at 1 MPa and 2 MPa is presented in Figures 11, 12, 13 and 14. No significant defect was detected on their propagation path. In contrary, none of the cracks passed through the mains defects previously identified in the specimen, as shown in Figures 15 and 16.
As sample #1 broke quicker than expected, the loads were adjusted for sample #2 to 0.6MPa, 1.2 MPa and 1.8 MPa.
In sample #2, no crack was detected at 0.6 and 1.2 MPa, as depicted in the CT sections in Figures 17 and 18. The sample suddenly broke at 1.8MPa, and presented multiple cracks as shown in Figure 19. The crack initiation sites could not be defined and no particular defect was found on the paths of the cracks. The cracks do not appear to have been influenced by the defects initially identified in the specimen, as shown in Figures 20 and 21.

6 ANALYSIS AND DISCUSSION

The dimensions of the specimen were higher than the standard values (DIN 51104). This may indicate that sintering was not fully complete.

CT scans indicated a relatively high but homogeneous distribution of porosity. The flat-disc-shaped pores were observed to lie in between successive layers created during the additive manufacturing process. This indicates that the adhesion between the successive layers was poor. Those delaminations were observed to cover large areas, even if their detection was complicated in the CT scans. The biggest of those interlaminar pores found in sample #3 was ranging between 450µm and 500µm. This represents approximately 10% of the specimen diameter.
The cone-shaped defect zone on one extremity of each of the two scanned specimens is believed to have been caused by an issue during the additive manufacturing process.

The standard deviation of the Ultimate Compressive Strength values, between the tested specimens reaches 11.6%, and the standard deviation of the Elasticity Modulus values is 20.8%. Considering the relatively high porosity and the cone-shaped defect zone found at the extremities of the specimens, the standard deviation could be expected to be higher.
The lunar regolith simulant used in this study (JSC-2A) was found to result in specimens with lower compressive properties than the ones obtained from lunar regolith simulant EAC-1, using the same additive manufacturing process parameters. Samples processed from the EAC-1 simulant[1] were measured to have an Ultimate Compressive Strength of 5.41 ± 0.29 [MPa], and an Elasticity Modulus of 403.34 ± 45.10 [MPa] (as compared with the values of 3.33 ± 0.39 [MPa] and 373.44 ± 77.60 [MPa] respectively, reported here for the JSC-2A specimens). This difference is most likely due to the difference in mineral composition between both simulants, but could also be partially linked to the higher defect concentration in the JSC-2A specimens.
Compression strength measured on regolith simulants processed by other techniques were reported to range from 2 to 20 [MPa]. The JSC-2A samples developed by Lithoz had a lower Ultimate Compressive Strength than other techniques involving binders (20.35 MPa [1], 14 to 19 MPa (for optimized parameters) [2]), but a slightly higher strength than solar-sintered regolith (2.31 ± 0.30 MPa [3]).

Concerning the results of compression-CT, it should be kept in mind that the tests were done by applying a certain load level, and maintaining it for half an hour up to one hour, before going to the next load level. Therefore, the results are not directly comparable to compression results, as crack could propagate during the load plateau. Results however show that the samples' initial defects did not cause the crack initiation, nor promoted the crack propagation.
As the samples also presented the cone-shape defect area at their base, and that the main crack observed in sample #1 originated from one extremity of the samples, it can be assumed that the cone-defect area played a role in the crack initiation. Unfortunately, the bottom and top of the samples being hidden by the compression test setup during compression-CT, this could not be verified. The top and bottom surfaces of the samples being non planar and non-parallel, this could also have caused some stress concentration, leading to crack initiation and failure of some corners of the specimens (as typically seen in Figures 15 and 19). A corner failure results in a smaller surface available to withstand the load, leading to a higher stress.

[1] See RD02- Lithoz – Regolith Compression Testing – RS0003855

This document may not be reproduced, except in full, without the written permission of the European Space Agency.

7 CONCLUSIONS AND RECOMMENDATIONS

Considering the relatively high porosity and the cone-shaped defect zone found in the specimens, the recorded standard deviations for the Young Modulus (20.8 %) and the Ultimate Compressive Strength (11.6 %) were found to be within reasonable levels. The Compressive Strength values measured in the additively manufactured specimens were found to be lower than other additively manufactured regolith simulant materials processed with binders, but slightly higher than solar sintered regolith simulant. Compression-CT revealed that the defects originally present in the sample had almost no effect on the crack initiation and propagation. On the other hand, the non-planar and non-parallel top and down surfaces, adding up to the cone-shape defect areas found next to them, could have played a major role in the crack initiation and specimen failure. Improving the process could potentially help to increase the measured material's compressive properties further, typically by decreasing the porosity, and especially the extremity defects, or by manufacturing samples with a more accurate geometry.

This document may not be reproduced, except in full, without the written permission of the European Space Agency.

8 BIBLIOGRAPHY

[1] G. Cesaretti, E. Dini, X. De Kestelier, V. Colla and L. Pambaguian, “Building components for an outpost on the Lunar soil by means of a novel 3D printing technology,” *Acta Astronautica,* vol. 93, pp. 430-450, 2014.

[2] S. L. Taylor, A. E. Jakus, K. D. Koube, A. J. Ibeh, N. R. Geisendorfer, R. N. Shah and D. C. Dunand, “Sintering of micro-trusses created by extrusion-3D-printing of lunar regolith inks,” *Acta Astronautica,* vol. 143, pp. 1-8, 2018.

[3] A. Meurisse, A. Makaya, C. Willsch and M. Sperl, “Solar 3D printing of lunar regolith,” *Acta Astronautica,* vol. 152, pp. 800-10, 2018.

9 APPENDICES

9.1 Incoming Inspection

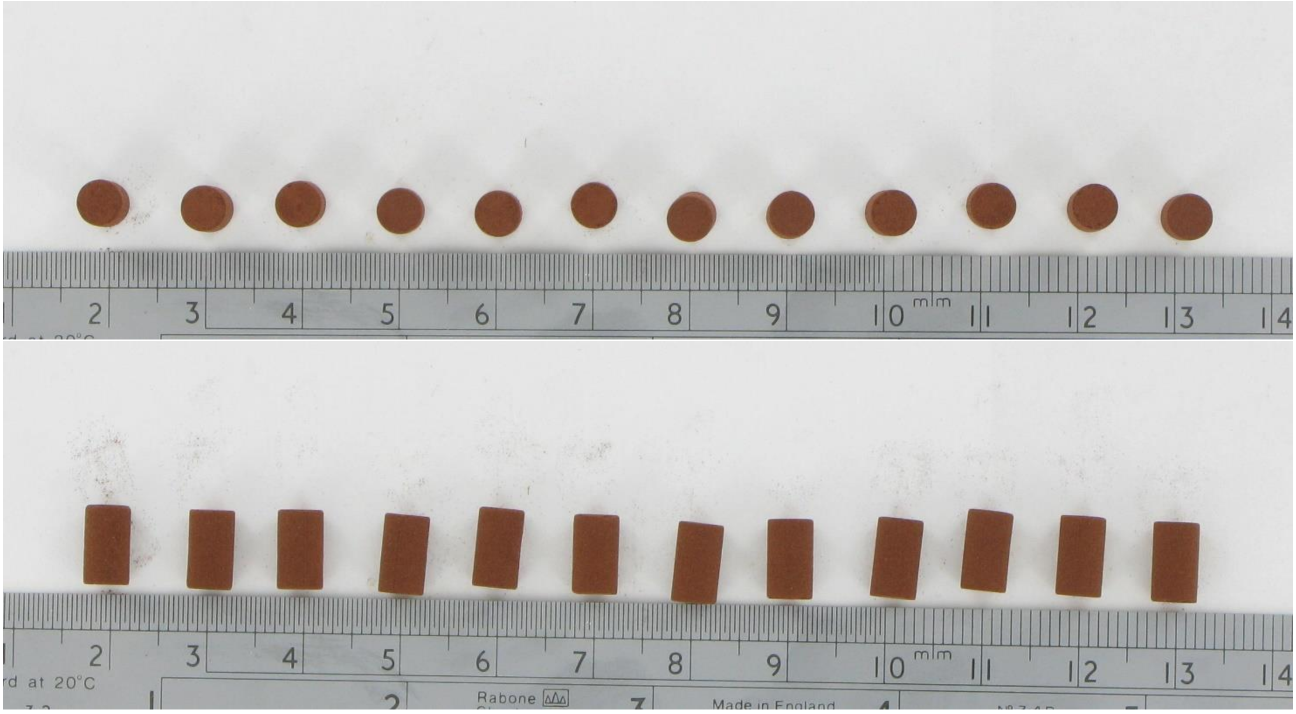

Figure 2: Side view (top) and top view (bottom) of as received specimens

This document may not be reproduced, except in full, without the written permission of the European Space Agency.

9.2 CT

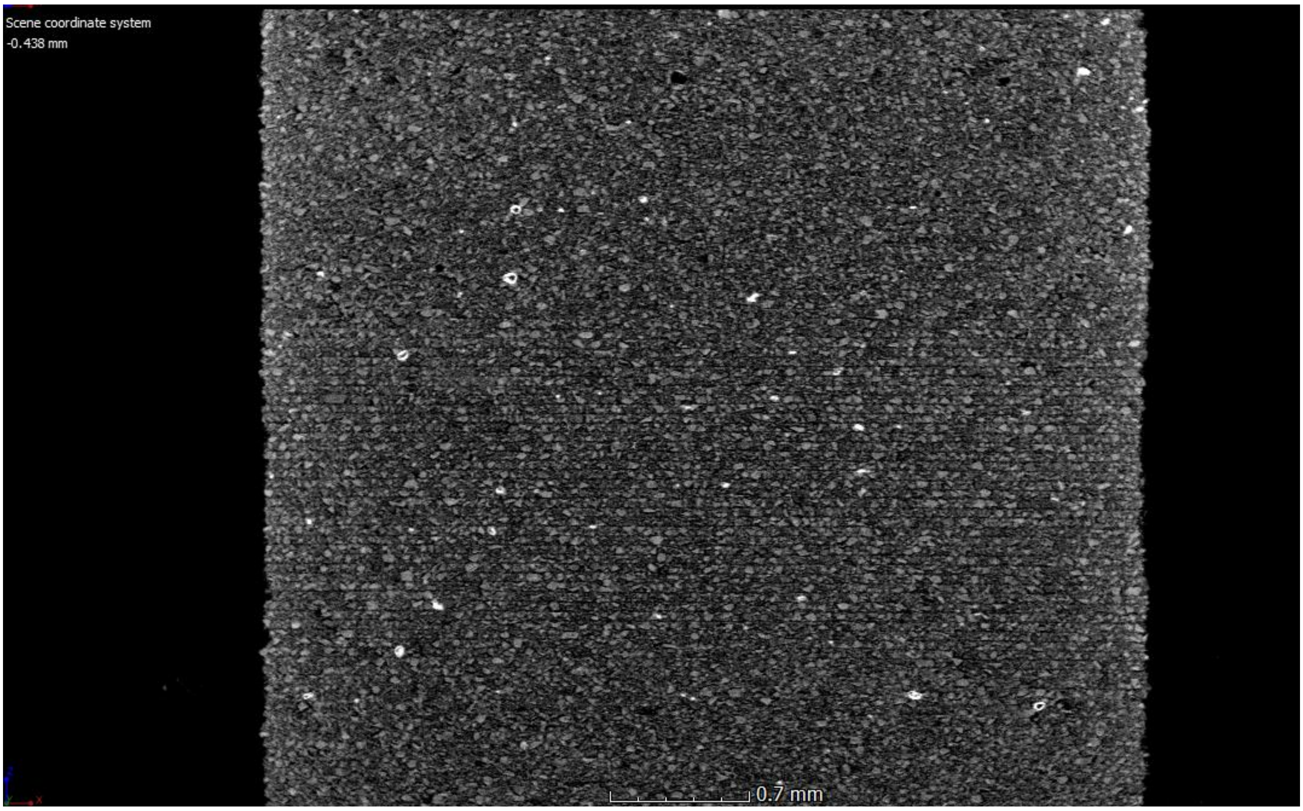

Figure 3: CT section, front view of specimen #1, showing the layered structure, typical to an additive layer manufacturing process

This document may not be reproduced, except in full, without the written permission of the European Space Agency.

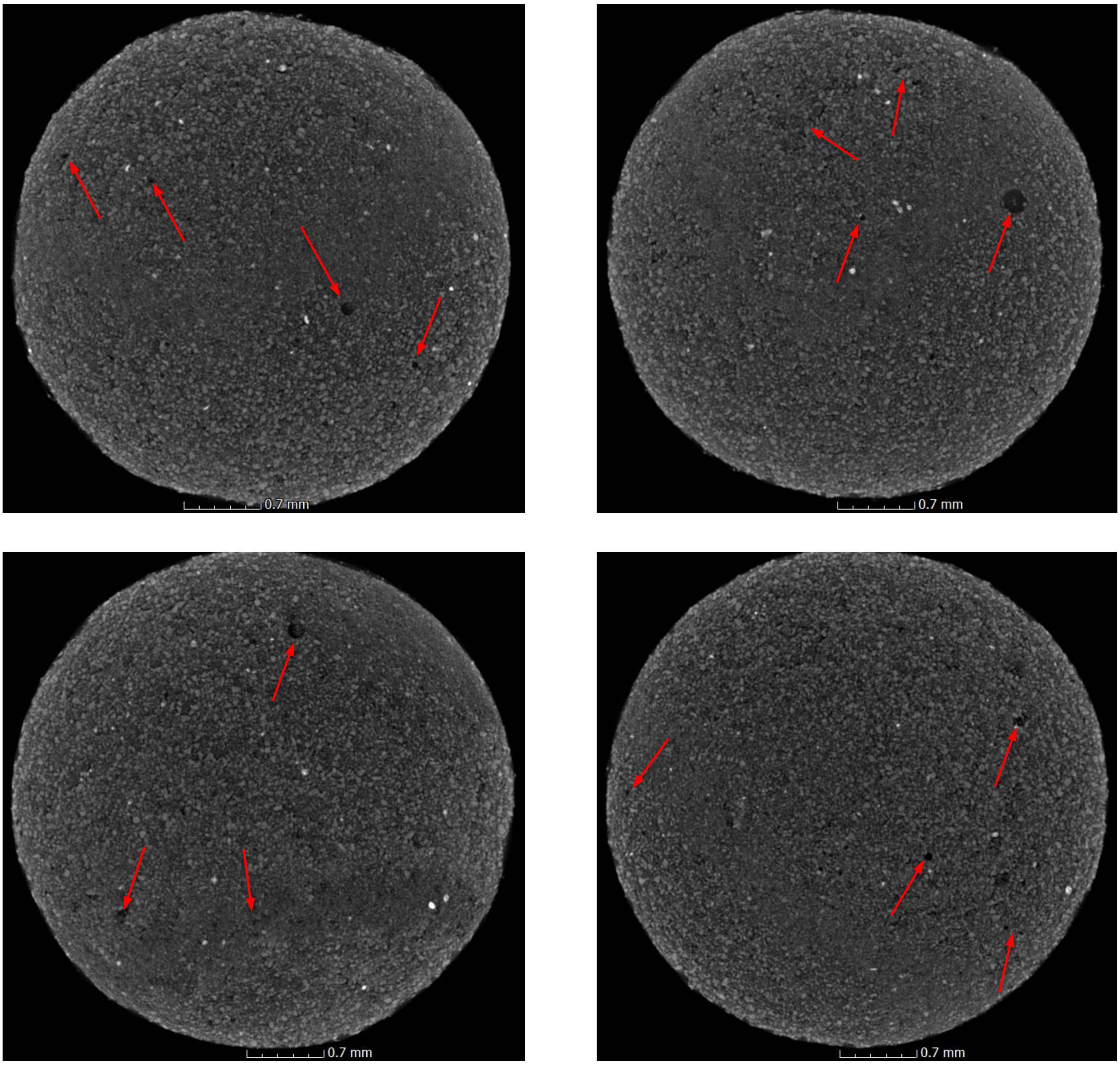

Figure 4: CT sections, top view of sample #3 in four different planes. Red arrows indicate some of the pores

This document may not be reproduced, except in full, without the written permission of the European Space Agency.

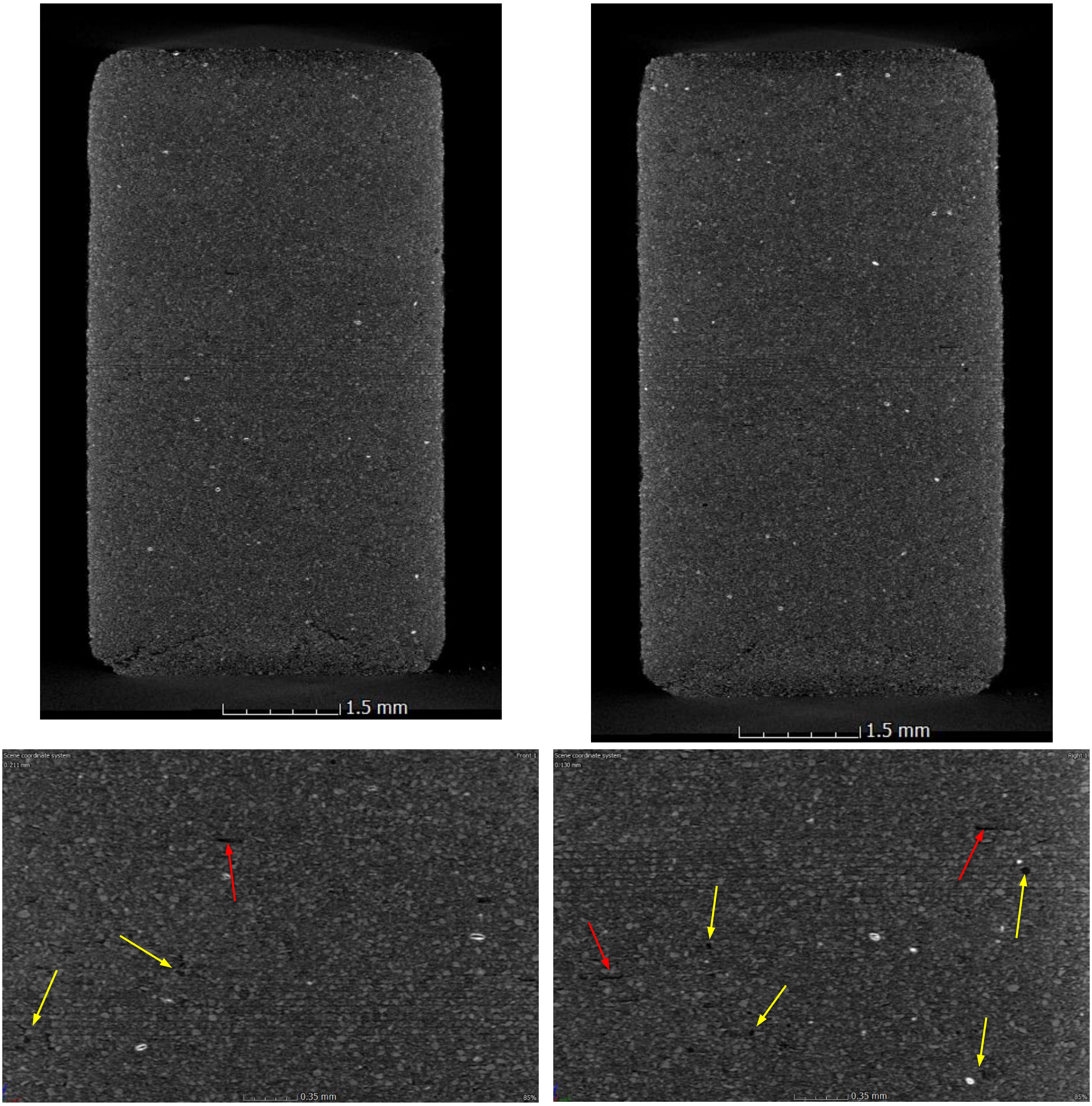

Figure 5: CT sections, side views of sample #3. Top: Overviews, Bottom: zoomed in to see the platelets and spherical porosities. Red arrows indicate the flat-disc pores, and yellow arrows indicate the quasi-spherical pores

This document may not be reproduced, except in full, without the written permission of the European Space Agency.

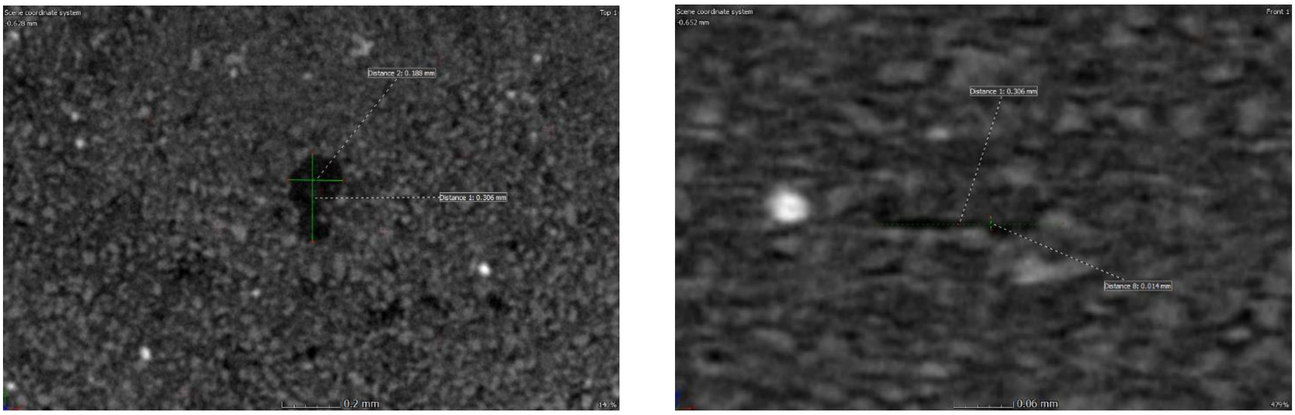

Figure 6: CT section of sample #3, in top and front view, showing a typical defect (flat disc morphology). Left: Top view, Right: Front view

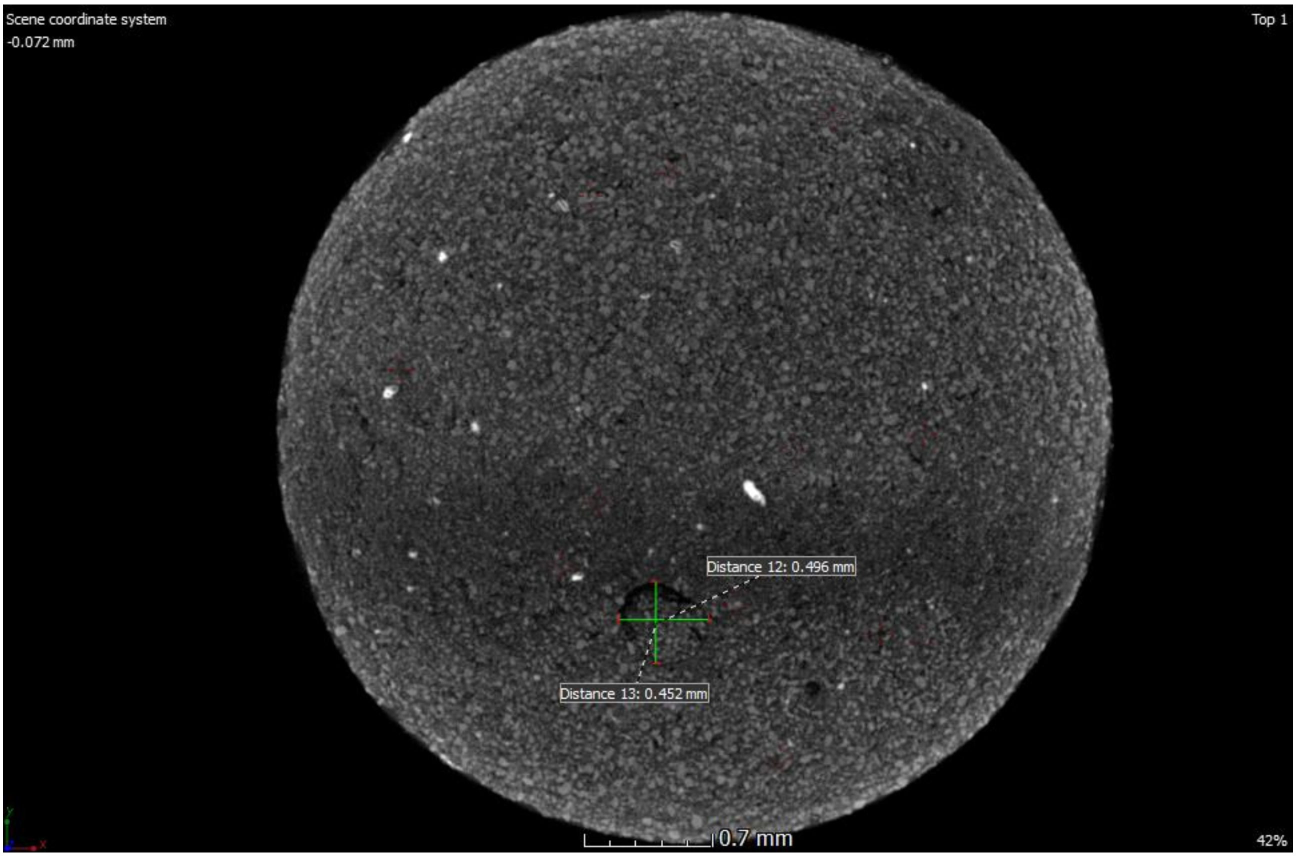

Figure 7: CT section of sample #3, showing the biggest identified flat-disc pore

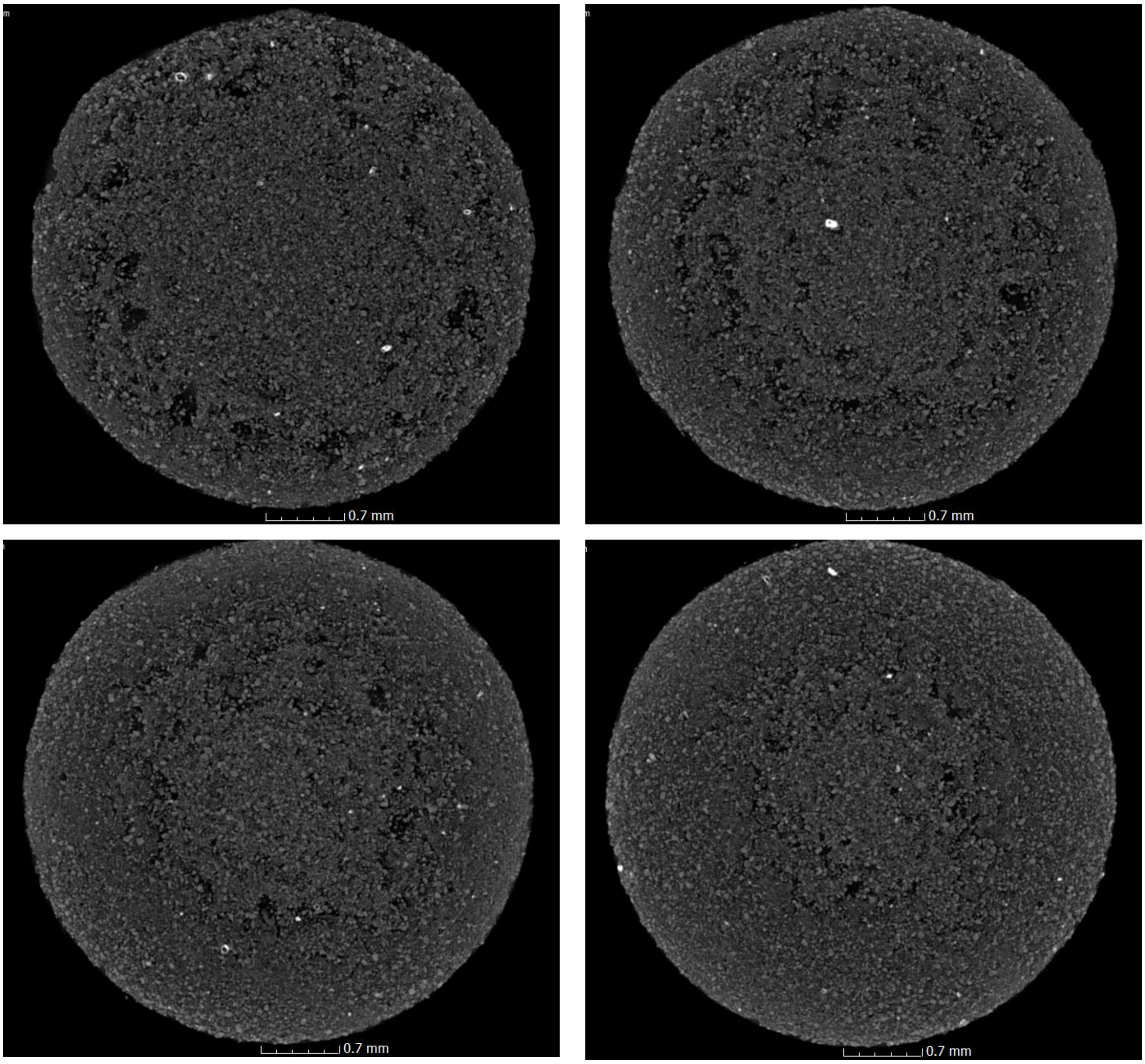

Figure 8: CT sections, top view of sample #3, in four different planes, showing the bottom defect

This document may not be reproduced, except in full, without the written permission of the European Space Agency.

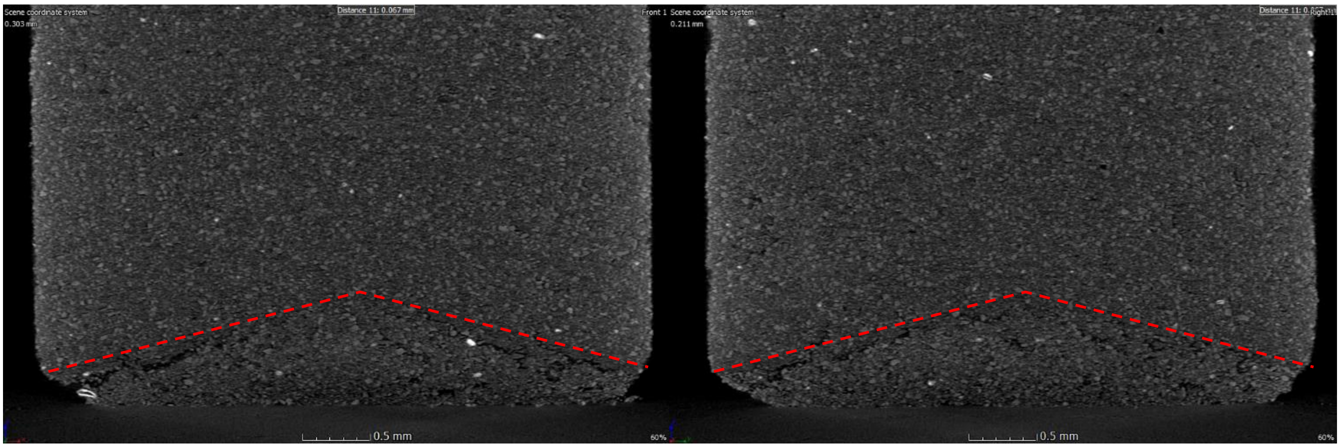

Figure 9: CT sections, front and side view of sample #3, showing the defect at its bottom. Red lines indicate the cone shape of the defective zone.

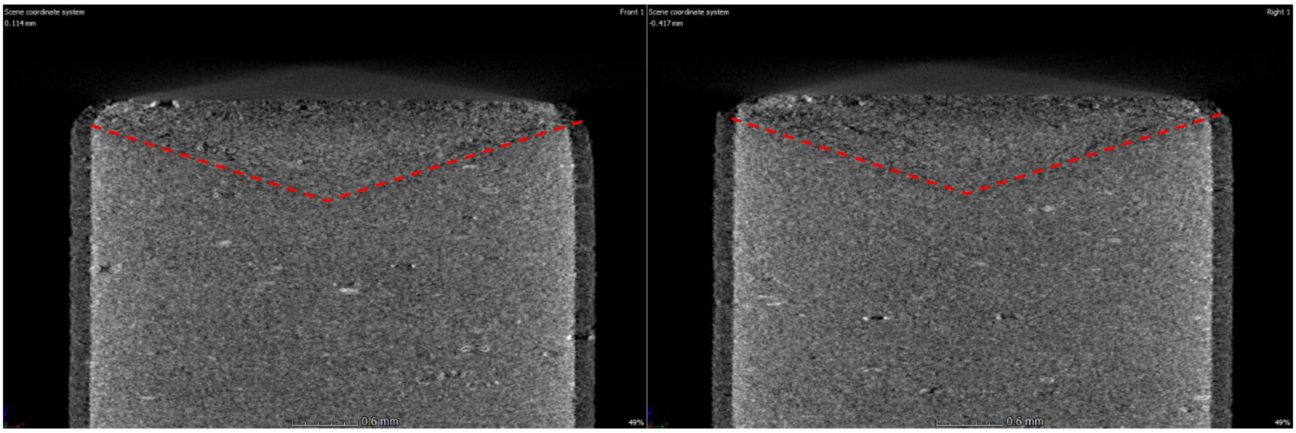

Figure 10: CT sections, front and side view of sample #4, showing the defect at its top. Red lines indicate the cone shape of the defective zone.

This document may not be reproduced, except in full, without the written permission of the European Space Agency.

9.3 Compression CT

9.3.1 Sample #1

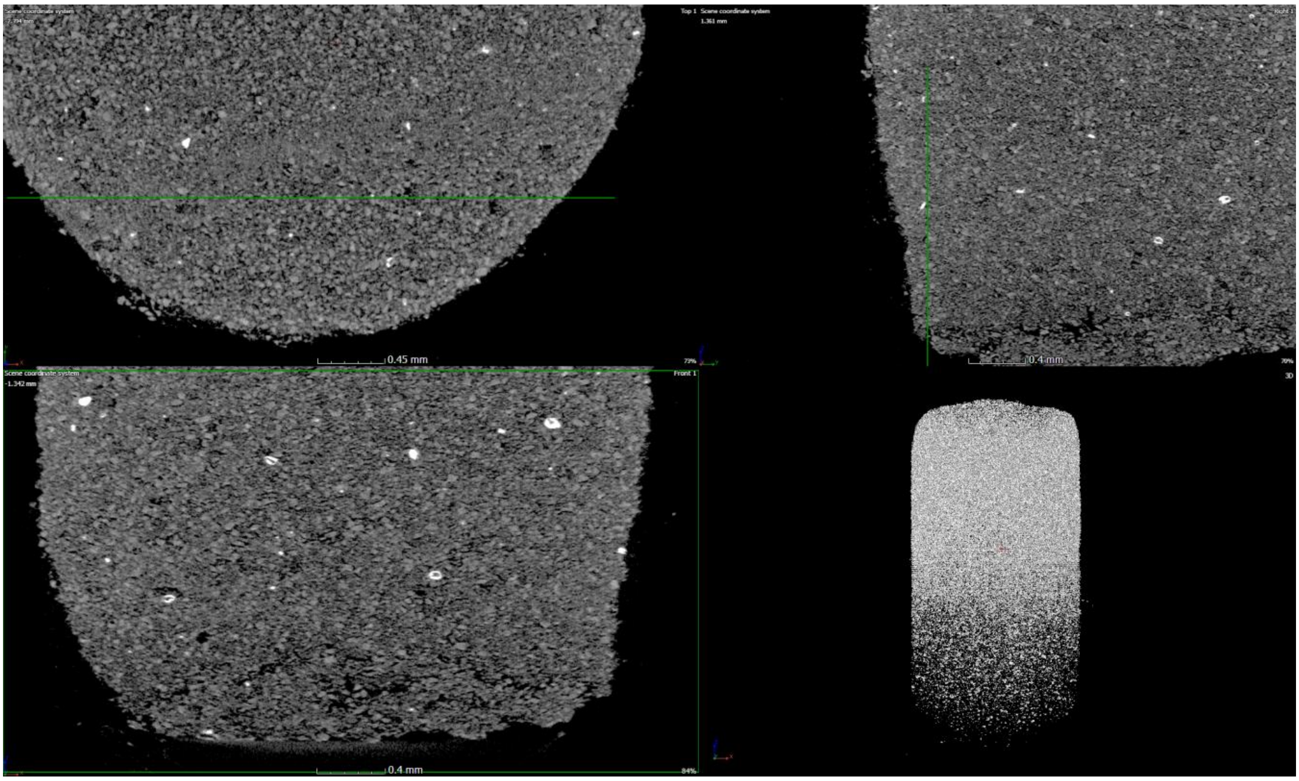

Figure 11: Portion of sample #1 before the initial crack appeared, 0N load (0MPa)

This document may not be reproduced, except in full, without the written permission of the European Space Agency.

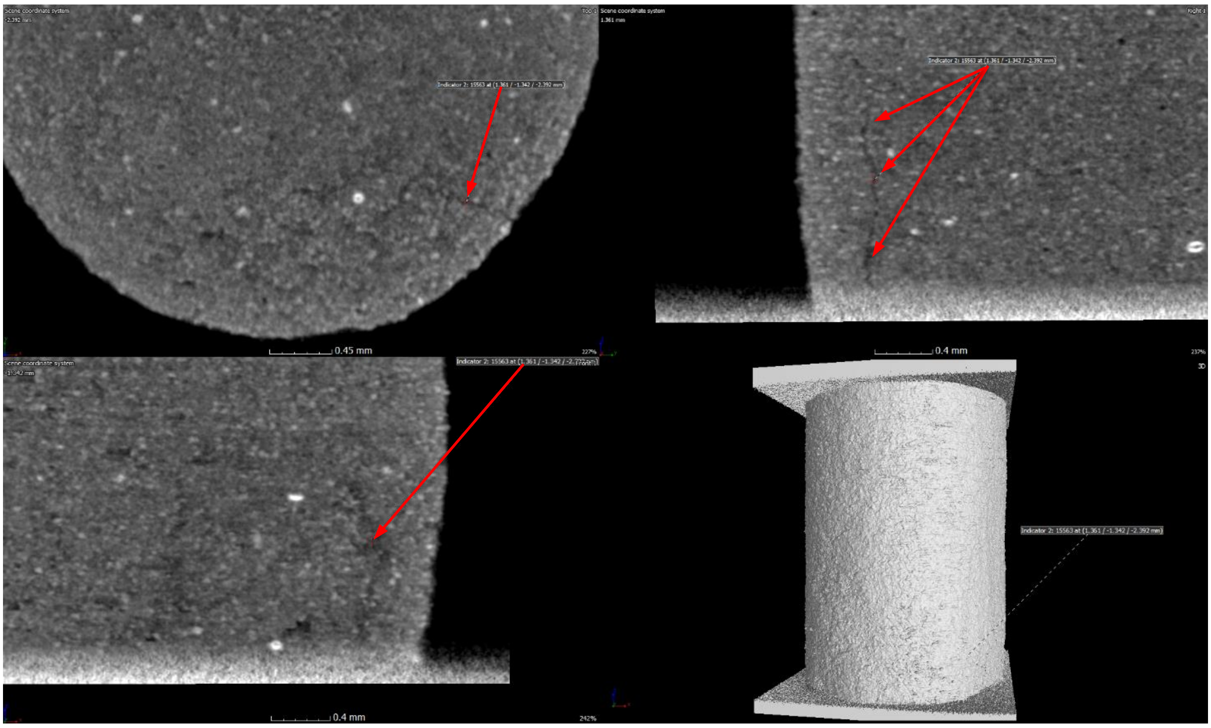

Figure 12: Portion of sample #1, with initial crack indicated by red arrows, 17N load (1MPa)

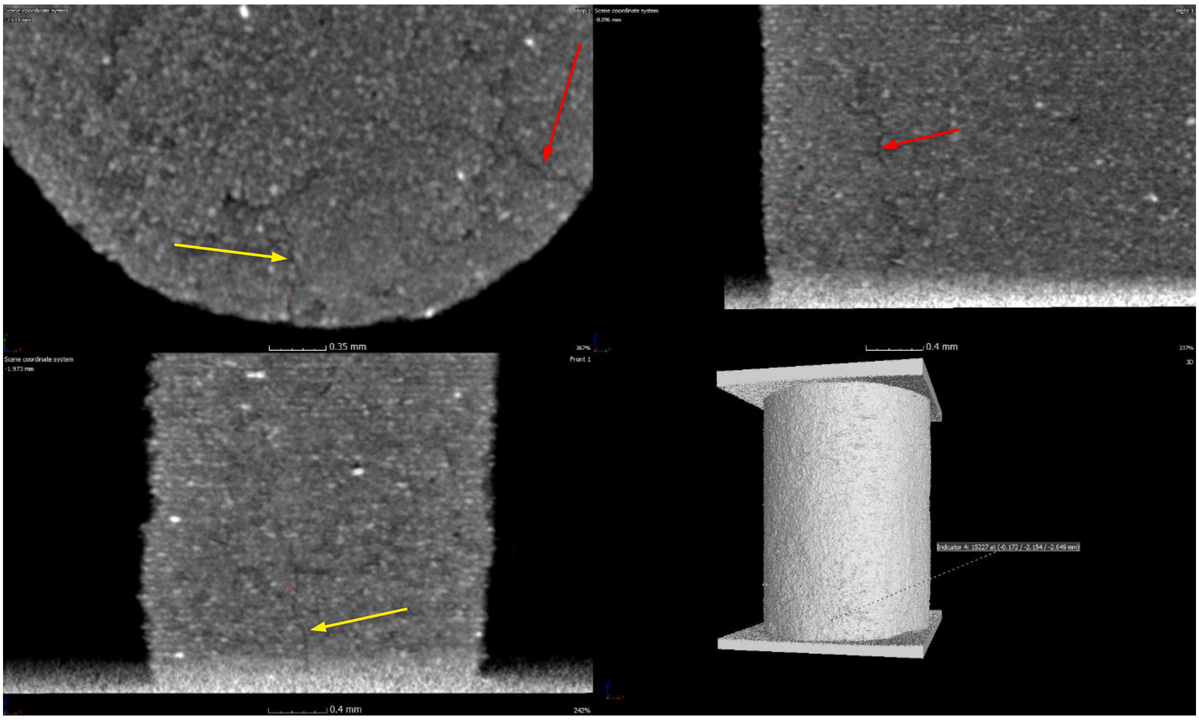

Figure 13: Portion of sample #1, showing a 2nd crack (yellow arrow) adjacent to the initial crack (red arrows), 17N load (1MPa)

This document may not be reproduced, except in full, without the written permission of the European Space Agency.

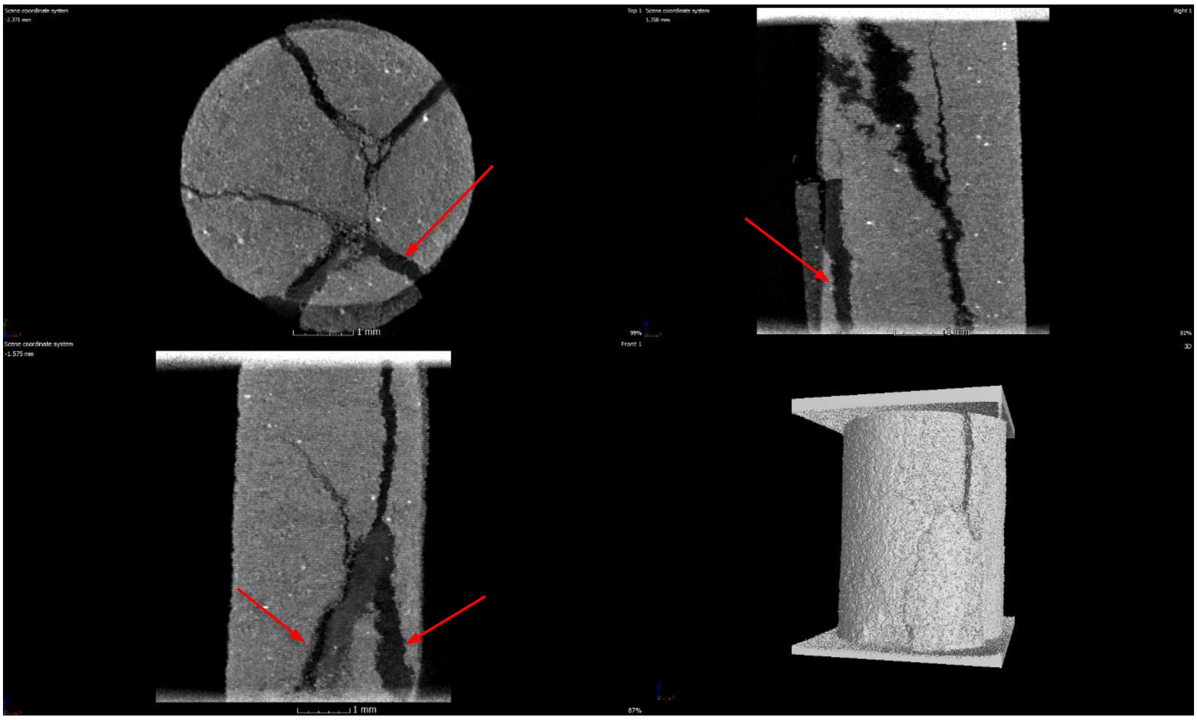

Figure 14: Sample #1 after failure, with initial cracks indicated by red arrows, 36N load (2MPa)

This document may not be reproduced, except in full, without the written permission of the European Space Agency.

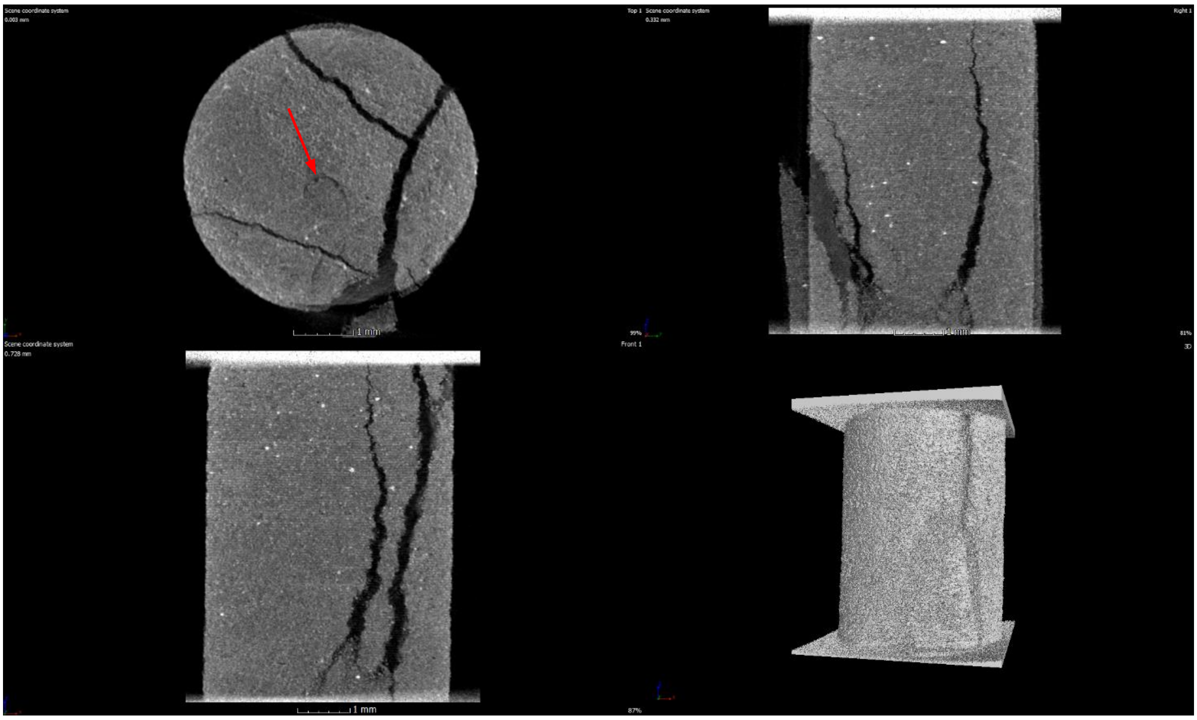

Figure 15: CT scan of sample #1, showing that the biggest flat disc pore (red arrow) observed in the sample did not affect the crack initiation and propagation, 36N load (2MPa)

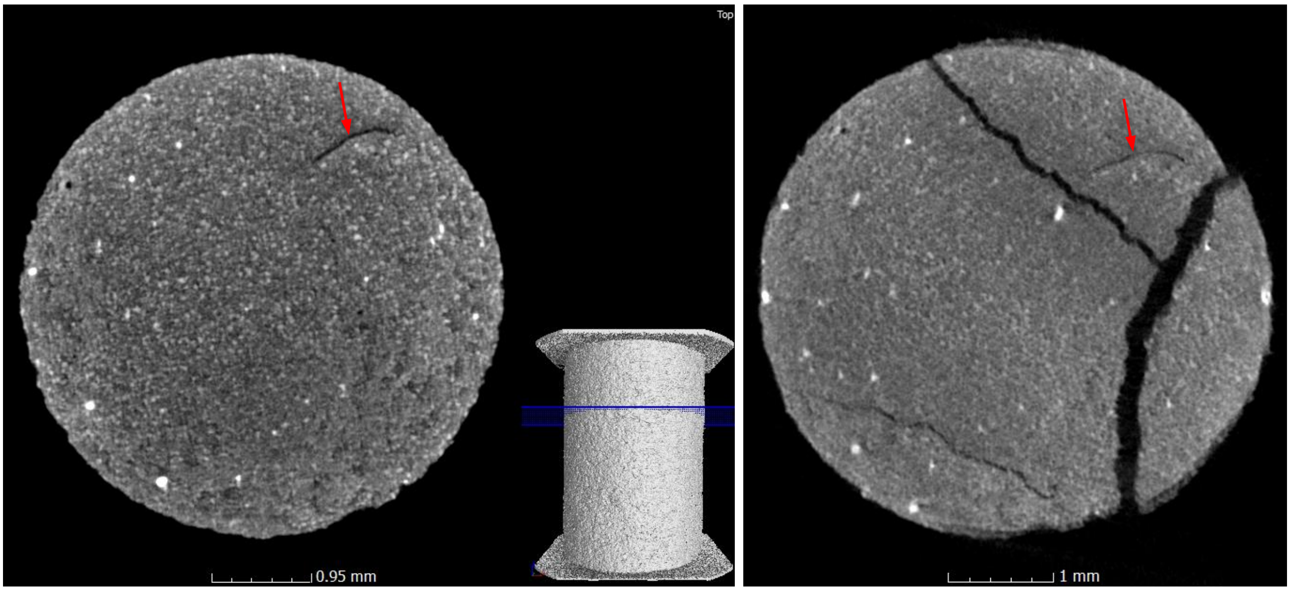

Figure 16: CT scan of sample #1, showing that an initial defect (red arrow) in the sample did not affect the crack initiation and propagation, 36N load (2MPa)

9.3.2 Sample #2

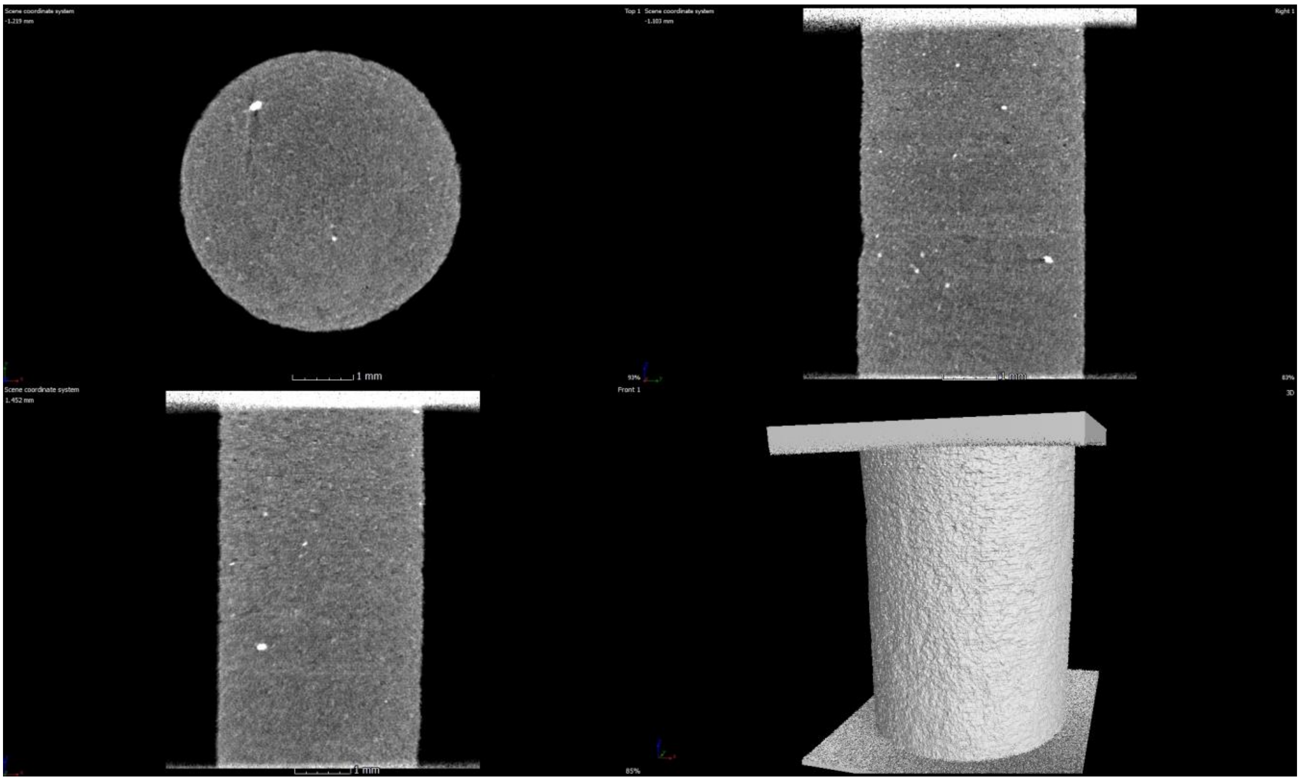

Figure 17: CT sections of sample #2. No major defect, nor crack detected, 10N load (0.6 MPa)

This document may not be reproduced, except in full, without the written permission of the European Space Agency.

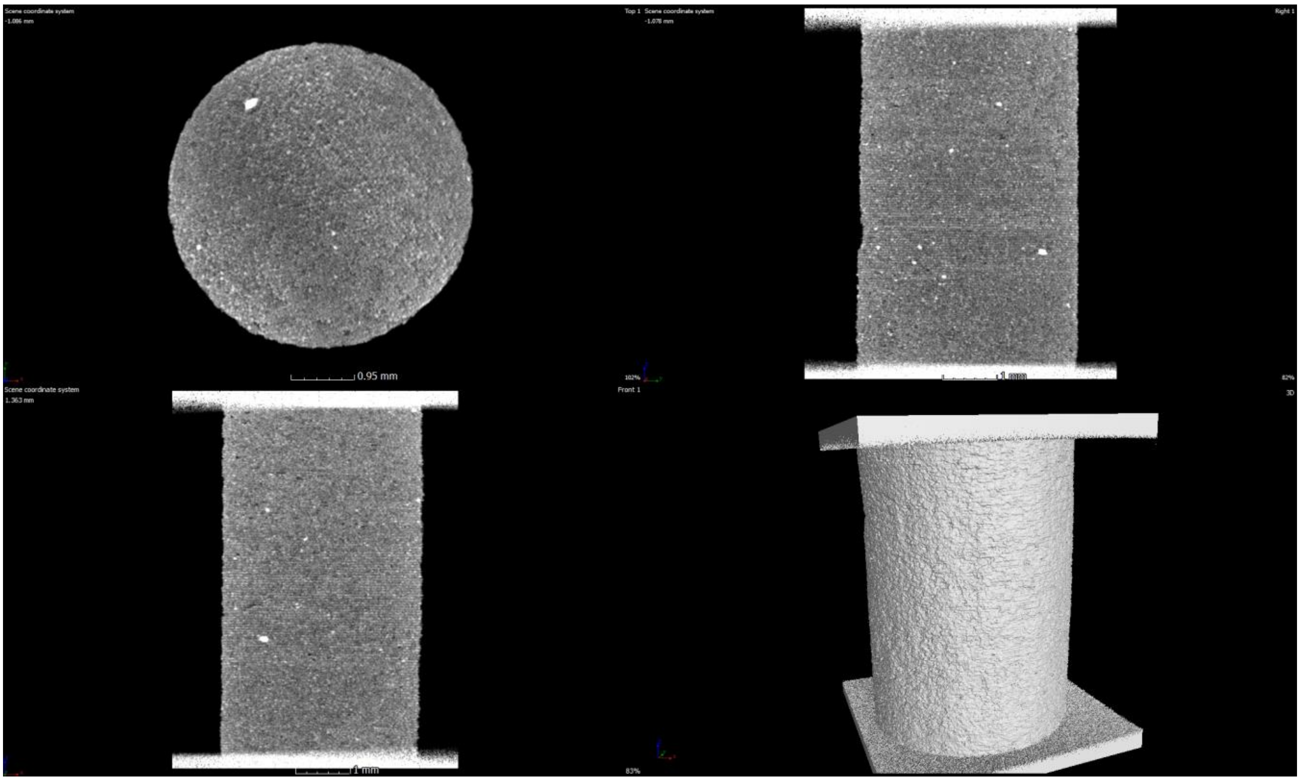

Figure 18: CT sections of sample #2. No major defect, nor crack detected, 20N load (1.2 MPa)

This document may not be reproduced, except in full, without the written permission of the European Space Agency.

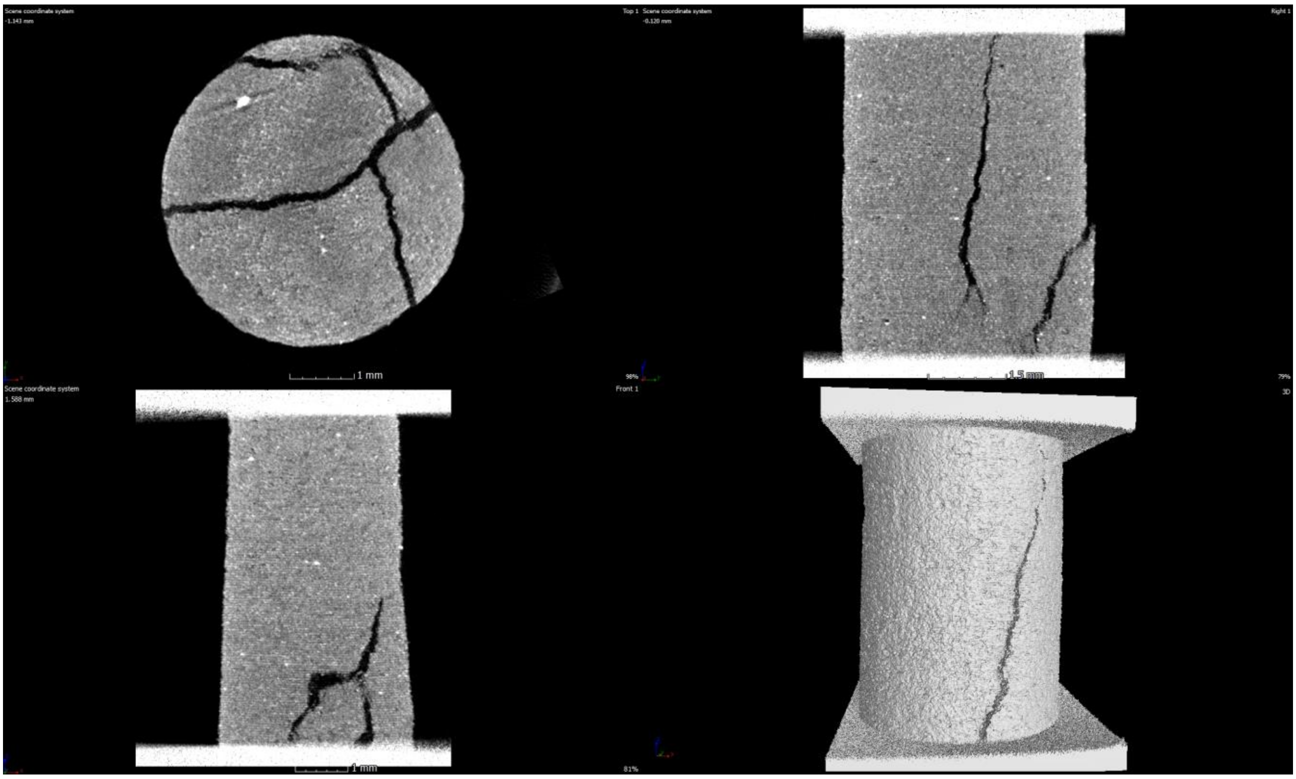

Figure 19: CT sections of sample #2. Sudden failure of the sample at 30N (1.7 MPa)

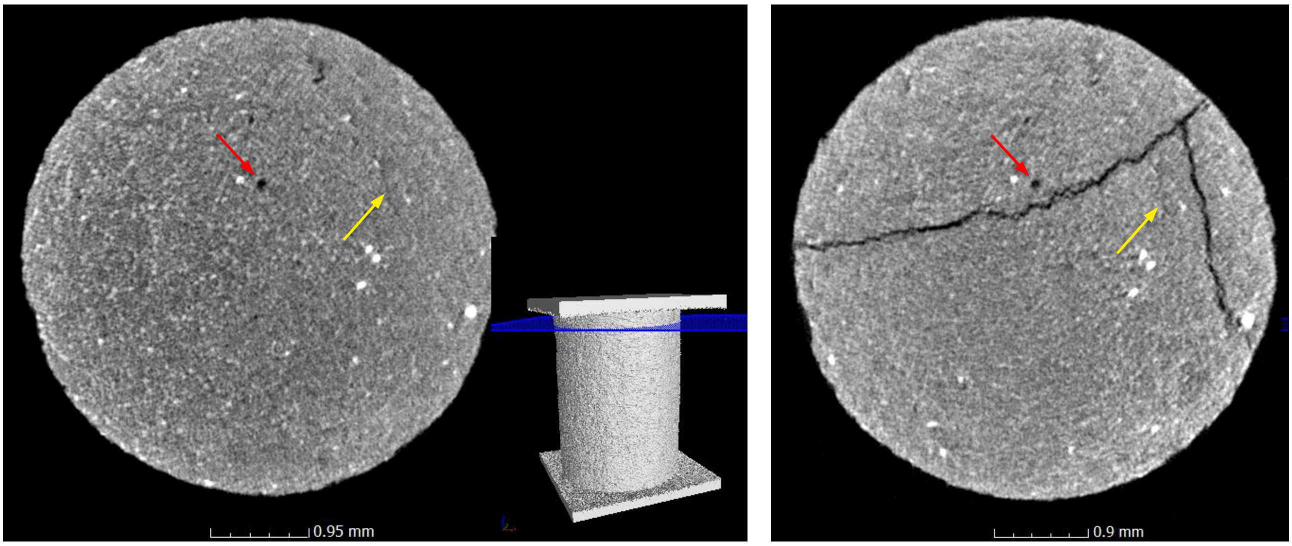

Figure 20: CT scans of sample #2, showing that initial defects (porosity - red arrow, small crack – yellow arrow) in the sample did not affect the crack initiation and propagation. Left: 20N load (1.2 MPa), Right: 30N load (1.7 MPa)

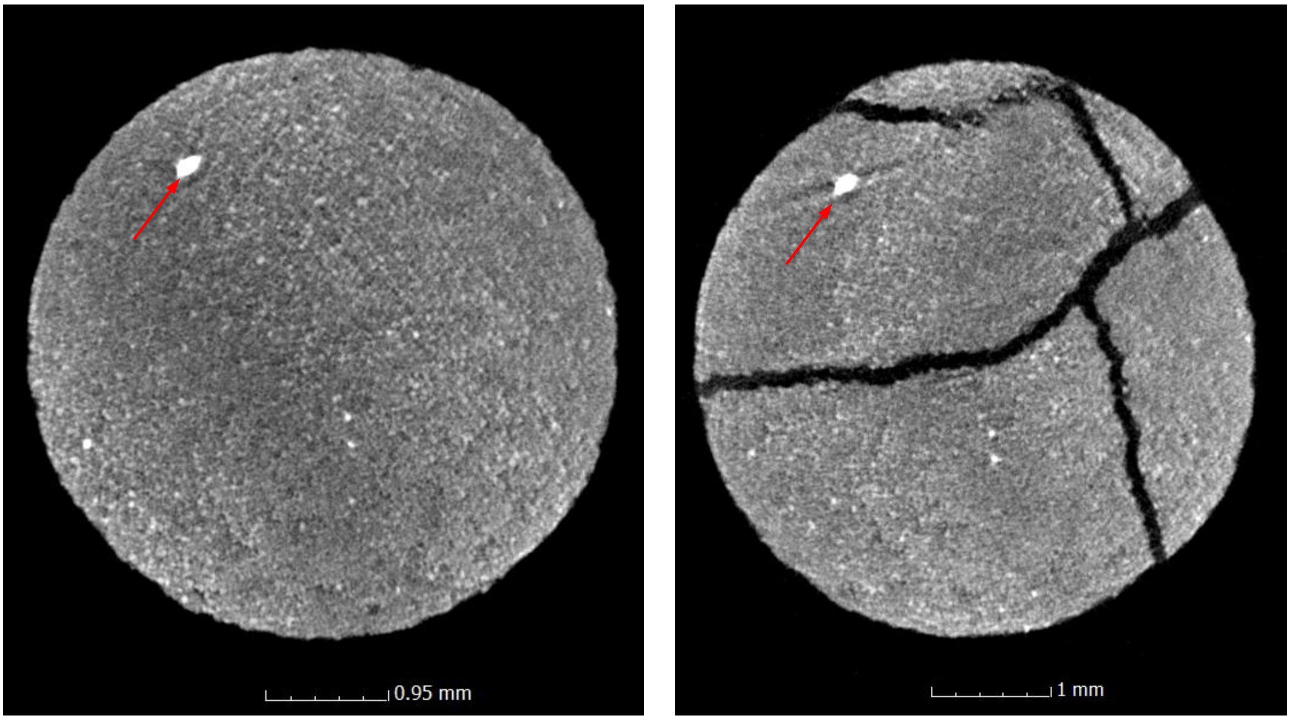

Figure 21: CT scans of sample #2, showing that the biggest observed inclusion (red arrow) did not affect the crack initiation and propagation. Left: 20N load (1.2 MPa), Right: 30N load (1.7 MPa)

This document may not be reproduced, except in full, without the written permission of the European Space Agency.
