# Supplementary material for: Iron can be microbially extracted from Lunar and Martian regolith simulants and 3D printed into tough structural materials
Source: PLoS One. 2021 Apr 28;16(4):e0249962. doi: 10.1371/journal.pone.0249962 (PMC8081250; doi:10.1371/journal.pone.0249962)
Supplement: S1 Data — (ZIP) [file pone.0249962.s001.zip › Data_updated/Strenght test/Report RS0004344_v4_Final.pdf]

estec

European Space Research
and Technology Centre
Keplerlaan 1
2201 AZ Noordwijk
The Netherlands
T +31 (0)71 565 6565
F +31 (0)71 565 6040
www.esa.int

DOCUMENT

TEST REPORT

RS0004344 - Compression of 3D Printed Regolith Iron-Enriched via Bacteria

RS0004344

Please complete the customer satisfaction survey at:
https://tecsurveys.esa.int/tecq

This document may not be reproduced, except in full, without the written permission of the European Space Agency.
Uncontrolled when printed.

**Prepared by**	**Maude Maréchal**
**Reference**	**ESA-TECMSP-TR-021221**
**Laboratory**	**ESA Materials and Electrical Components Laboratory**
**Area**	**TEC-MSP**
**Issue**	**1**
**Revision**	**1**
**Date of Issue**	**03/11/2020**
**Document Type**	**TR**

European Space Agency
Agence spatiale européenne

APPROVAL

RS0004344 - Compression of 3D Printed Regolith Iron-Enriched via Bacteria	
Issue 1	Revision 1
Author Maude Maréchal (TEC-MSP)	Date: 03-Nov-2020
**Reviewed by**	**Date**
Advenit Makaya(TEC-MSP)	03-Nov-2020 Advenit Makaya Digitally signed by Advenit Makaya Date: 2021.02.28 21:21:43 +01'00'
**Approved by**	**Date**
Thomas Rohr, Line Manager	03-Nov-2020

Please refer to document database for electronic approval evidence.

CHANGE LOG

Reason for change	Issue	Revision	Date
Initial Release	1	1	28/02/2021

Please refer to document database for electronic version history.

CHANGE RECORD

Issue 1	Revision 1		
**Reason for change**	**Date**	**Pages**	**Paragraph(s)**

DISTRIBUTION LIST

This document may not be reproduced, except in full, without the written permission of the European Space Agency.

Table of contents:

EXECUTIVE SUMMARY **4**
1 INTRODUCTION **5**
2 REFERENCE DOCUMENTS **5**
3 SAMPLE(S) **5**
3.1 Description / Basic Information 5
4 EXPERIMENTAL **6**
4.1 Test and Test Method 6
5 RESULTS **7**
5.1 CT 7
5.2 Compression testing 7
5.3 Compression-CT 8
6 ANALYSIS AND DISCUSSION **10**
7 CONCLUSIONS AND RECOMMENDATIONS **11**
8 REFERENCES **12**
9 APPENDICES **12**
9.1 Incoming Inspection 12
9.2 CT 15
9.3 Compression-CT 19

This document may not be reproduced, except in full, without the written permission of the European Space Agency.

EXECUTIVE SUMMARY

By using bacteria, lunar regolith simulant JSC-2A has been enriched in iron by the team at TU-Delft. This biologically processed powder was then extracted magnetically from the rest of the regolith batch, leading to an iron-enriched regolith simulant feedstock. This feedstock was used by Lithoz GmbH to produce cylindrical specimens via Lithography-based Ceramic Manufacturing (additive manufacturing). It was decided that ESA would perform compressive testing and Computer Tomography (CT) scanning, to support a future joint publication between TU-Delft, Lithoz and ESA. Two kinds of specimen were tested: specimens sintered at 1050ºC and specimens sintered at 1100ºC. CT scans were performed on 2 specimens (1 of each kind), and compression tests on 6 specimens (1 sintered at 1050ºC and 5 sintered at 1100ºC). In addition, some Compression-CT testing (i.e. compression testing during CT scanning) were performed on 1 specimen, sintered at 1100ºC.

The samples sintered at 1050ºC were observed to be extremely brittle. CT scans revealed a very low density, indicating a severe lack of sintering.

For samples sintered at 1100ºC, very large defects were observed, mainly in the centre of the samples. Those defects were either big porosity clusters, or interlaminar porosity. Those defects are believed to be resulting from an insufficient quantity powder feedstock, which prevented a fine milling. This led to coarser particles and agglomerates, which caused difficulties in spreading the suspension of regolith simulant powder and binder, during the additive manufacturing process.

The Ultimate Compressive Strength was measured to be 13.18 ± 3.49 [MPa][1].

This is significantly higher than results previously obtained on samples manufactured from the same JSC-2A regolith simulant, using the same additive manufacturing process, but without the bacterial pre-treatment. The measured Ultimate Compressive Strength was indeed measured to be 3.33 ± 0.39 [MPa][2] for untreated JSC-2A regolith simulant. By using an untreated EAC-1 regolith simulant, an Ultimate Compressive Strength of 5.41 ± 0.29 [MPa][3] was achieved.

Compression-CT testing revealed that the defects originally present in the sample had a notable influence on crack propagation. Both pores and higher-density inclusions were found on the path of the cracks.

---

[1] For the samples sintered at 1100ºC

[2] See RD03 - RS0004277 - Lithoz – Compression Testing of Lunar Regolith Simulant JSC-2A specimens

[3] See RD02 - Lithoz – Regolith Compression Testing – RS0003855

This document may not be reproduced, except in full, without the written permission of the European Space Agency.

1 INTRODUCTION

By using bacteria, lunar regolith simulant JSC-2A was enriched in iron by the team at TU-Delft. This biologically processed powder was then extracted magnetically from the rest of the regolith batch, leading to an iron-enriched regolith simulant feedstock. This powder feedstock was used by Lithoz GmbH to produce cylindrical specimens via Lithography-based Ceramic Manufacturing (additive manufacturing). ESA, Lithoz and TU Delft decided to prepare a joint publication on that study. To support the preparation of the publication, it was decided that ESA would perform the compressive testing following the DIN 51104 standard. Computerized Tomography (CT) was also used to check the porosity of the samples. On top of that, Compression-CT was used to observe the crack formation.

2 REFERENCE DOCUMENTS

No.	Report Version	Document title
RD01	01-2018-08-22	Feasibility Study – Report FS_20180605
RD02	17-01-2020	Lithoz – Regolith Compression Testing – RS0003855
RD03	07-04-2020	Lithoz – Compression Testing of Lunar Regolith Simulant JSC-2A specimens – RS0004277

3 SAMPLE(S)

3.1 Description / Basic Information

Eight cylindrical samples were received at ESTEC. The sample geometry was defined in accordance with the DIN 51104 standard. Two of the specimens where sintered at 1050ºC, and the six others were sintered at 1100ºC. Figures 2 (sintering at 1050ºC) and 3 (sintering at 1100ºC) show as received specimens, in top and side views. The two specimens sintered at 1050ºC were extremely brittle, and arrived already damaged. Any handling on those samples caused some material loss, in form of free powder, as shown in Figure 5.

The mean dimensions measured on the two types of samples are displayed in Table 1. Samples sintered at 1100ºC were within the standard's tolerance (Diameter of 4.5mm ± 0.1mm / Height of 8.0mm ± 0.2mm). The samples nevertheless presented important distortion, as highlighted in Figure 4.

On the other hand, samples sintered at 1050ºC were clearly out of the tolerance, partially because they were received already damaged.

This document may not be reproduced, except in full, without the written permission of the European Space Agency.

Table 1: Mean dimensions of the specimens. Considering the high friability of samples sintered at 1050ºC, their dimensions are only indicative, as it was impossible to measure them properly.

Type of sample	Height [mm]	Diameter [mm]
Sintered at 1050ºC	≅ 4.3	≅ 8.5
Sintered at 1100ºC	4.46 ± 0.02	7.98 ± 0.07

4 EXPERIMENTAL

4.1 Test and Test Method

In order to evaluate the quality of the parts, CT scans were performed on two different specimens (respectively #1 and #3). This was made with a Phoenix V|tome|x m 300 kV from General Electric. Compression testing during CT scanning (later referred to as Compression-CT) was performed on sample #3. This test was conducted in the Phoenix V|tome|x m 300 kV CT equipment, together with a Deben Microtest Module (CT5000 5kN in-situ tensile/compression stage). A scanning voltage of 80kV and a current of 70µA were used.
Compressive tests were performed with an Instron ElectroPuls E10000 Linear-Torsion, on sample #2 and on samples #4 to #8. Room conditions: 22°C, about 50%rel. humidity. DIN 51104 standard was followed, expect for two deviations:

1. No intermediate plate was used between the machine and the specimens. This was considered not needed, as the parts were known to be quite brittle and unlikely to damage the machine.
2. The samples were not compliant to the standard, as their end-surfaces were not parallel. This was considered to have a limited impact on the determination of the Ultimate Compressive Strength and the Elasticity Modulus (provided that the modulus calculation does not account for the initial part of the curve).

A constant deformation rate of 0.5mm/min was used. The Ultimate Compressive Strength was calculated by considering the maximum load the sample withstood and its initial diameter. The Elasticity Modulus was not calculated for those samples, due to the plateau observed in the middle of the elastic region.

For the Compression-CT, the microtest compression setup was put in the CT machine. The deformation rate was also set at 0.5mm/min. The moving compression plate was initially put in contact with the sample by applying a 3N preload (deformation rate of 0.5mm/min, 0.2MPa). The first desired load level was then applied and maintained, and a CT scan was run. Each scan was between 30 minutes and 1h long. The next load level was then applied, and a new scan run. This was continued until full failure of the sample. Due to the presence of the setup around the samples, the top and bottom parts of each sample were not possible to scan.

This document may not be reproduced, except in full, without the written permission of the European Space Agency.

5 RESULTS

5.1 CT

Typical sections of the CT scans are presented for each scanned specimen, in Figures 6 to 11 in the Appendices.
Figures 6 and 7 show the low density of sample #1, which was sintered at 1050ºC. In particular, on Figure 7, it is clear that the interconnection between adjacent regolith particles is very poor, as denoted by the dark channels surrounding individual particles.
Figures 8 and 9 show CT scan slices from sample #3 (sintered at 1100ºC) in top and side views respectively. They show different types of defects found in the sample. This includes round-shape pores of various sizes (e.g. see bottom left of Figure 8), but also very large pores and interlaminar porosity . Most of the defects were concentrated in the centre of the sample. Figure 10 shows more specifically the biggest interlaminar pore. Its dimensions from top-view were measured to be around 1.7mm in diameter, whereas its thickness was under 0.04mm. Figure 11 shows a defect cluster. Its biggest pore was measured to be around 0.8mm in diameter and 0.3mm in thickness.

5.2 Compression testing

The only sample sintered at 1050ºC that was tested had a very low Ultimate Compressive Strength of approximately 0.1 [MPa].
Table 2 displays the Ultimate Compressive Strength for each specimen sintered at 1100ºC that was tested. The average Ultimate Compressive Strength was found to be 13.18 ± 3.49 [MPa].
The stress-strain curves for compression testing of the samples globally presented similar features for all the samples, as shown in Figure 1.
Table 3 summarizes the compression results obtained for JSC-2A Lunar Regolith Simulant specimens (both untreated and iron-enriched), together with the results obtained with the same process on an EAC-1 Lunar Regolith Simulant.

Table 2: Ultimate Compressive Strength overview for iron-enriched JSC-2A specimens

Sample	Ultimate Compressive Strength [MPa]
4	18.32
5	10.74
6	14.70
7	9.46
8	12.68
**Mean**	**13.18**
**STD**	**3.49**

This document may not be reproduced, except in full, without the written permission of the European Space Agency.

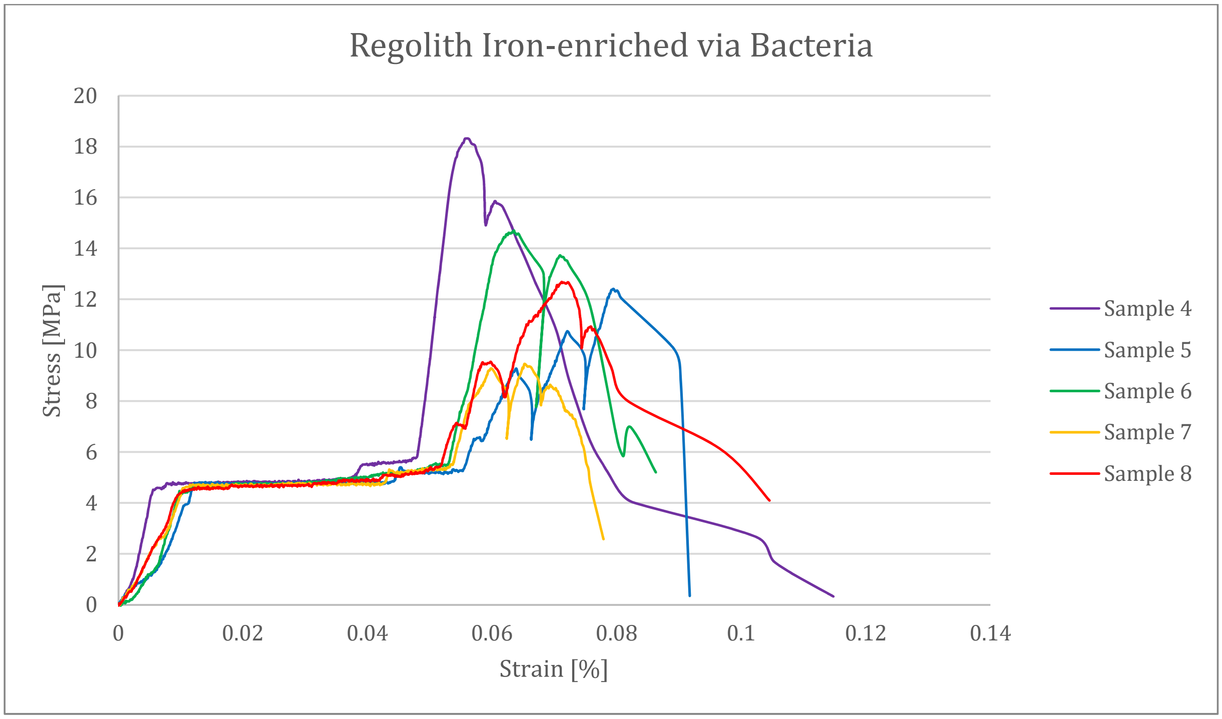

Figure 1: Stress-strain curves of compression tested specimen

Table 3: Overview of the compressive test results obtained for specimens manufactured from Lunar Regolith Simulants JSC-2A (untreated and iron-enriched) and EAC-1

Type of powder	Compressive Strength [MPa]	Elasticity Modulus [MPa]
Lunar Regolith JSC-2A untreated	3.33 ± 0.39	373.44 ± 77.60
Lunar Regolith JSC-2A bacterially iron-enriched and magnetically extracted	13.18 ± 3.49	Not calculated
Lunar Regolith EAC-1	5.41 ± 0.29	403.34 ± 45.10

5.3 Compression-CT

Considering the standard cmpression results and the results obtained for untreated powder, it was initially decided to test the samples by beginning at a load of 15N and with 15N increments until failure. This resulted in the following stresses: 1MPa, 1.9MPa, 2.9MPa, 3.9MPa, 4.8MPa, 5.8MPa, 6.8MPa, 7.7MPa, 8.7MPa and 9.7MPa.
The first crack appeared on one corner of the sample at a stress of 4.8 MPa, as shown in Figure 12. No particular defect was seen on its propagation path. The test continued, as the sample was still able to withstand the load. The second and third cracks appeared at 6.8MPa, as shown in Figures 13 and 14. The second crack was located close to a large porosity and

This document may not be reproduced, except in full, without the written permission of the European Space Agency.

could have initiated from this defect. The third crack shown in see Figure 14 was later identified as a continuation of the first crack reported in Figure 12. The cracks path passed through several defects, including higher density inclusions.
At 7.7MPa, the cracks propagated (see Figures 15 and 16), without causing an entire failure of the specimen. The second crack became the main crack, whereas 1st and 3rd cracks only affected a corner of the sample. As shown in Figure 17, the main crack separated the samples in two and was already longer than the half specimen.
At 8.7MPa, the cracks only grew a bit lengthwise, but mainly became wider, as shown in Figures 18 and 19. At 9.7MPa, the sample failed completely, as the crack propagated until its bottom, as shown in Figure 20. Some of the biggest porosity defects initially present in the specimen collapsed (see Figure 21), while sub-cracks appeared at various places (see Figures 21 and 22).

This document may not be reproduced, except in full, without the written permission of the European Space Agency.

6 ANALYSIS AND DISCUSSION

Based on the measured specimen dimensions, the observed friability and the low compressive strength of the samples sintered at 1050ºC, it is considered that sintering was only partial.

Concerning the samples sintered at 1100ºC, the size of the defects was larger than expected. No such porosities/delaminations were found in the previous samples produced by Lithoz[4,5]. The samples were also abnormally distorted. Those different defects most probably originated from the small quantity of powder feedstock available for the additive manufacturing process. This did not allow to perform an intensive milling, resulting in coarser particles and agglomerates being fed into the additive manufacturing process. As an attempt to mitigate this suboptimal powder feedstock, Lithoz decided to increase the sintering temperature from 1050ºC to 1100ºC. Nevertheless, this coarser powder resulted in a different rheological behaviour of the powder-binder suspension used during the additive manufacturing. This facilitated the trapping of air bubbles and caused a more difficult spreading of the suspension, leading to macroscopic pores.

Compression test results are globally consistent among the 5 tested specimens. They all present a plateau at around 5 [MPa], and multiple load peaks. Those features are difficult to explain, but could be linked to the important porosity found.
The standard deviation of the Ultimate Compressive Strength reaches 26.5%.
Bacterial iron enriching of the Lunar Regolith Simulant JSC-2A led to higher compressive properties of the additively manufactured specimens. Untreated JSC-2A Lunar Regolith Simulant specimens were indeed measured to have an Ultimate Compressive Strength of 3.33 ± 0.39 [MPa] (as compared with the iron-enriched value of 13.18 ± 3.49 [MPa]). Those results are also significantly higher than those reported for the Lunar Regolith Simulant EAC-1 specimens, which were found to have an Ultimate Compressive Strength of 5.41 ± 0.29 [MPa].
This indicates that optimizing the initial regolith raw material could potentially increase the quality of the final manufactured parts, leading to higher Ultimate Compressive Strength.
Compression strength measured on regolith simulant specimens processed by other techniques were reported to range from 2 to 20 [MPa].
The iron-enriched samples developed by TU-Delft and Lithoz had an Ultimate Compressive Strength slightly lower than other techniques involving binders (20.35 MPa [1], 14 to 19 MPa (for optimized parameters) [2]), but significantly higher than solar-sintered regolith (2.31 ± 0.30 MPa [3]).

Concerning the results of compression–CT testing, it should be kept in mind that the tests were done by applying a certain load level, and maintaining it for half an hour up to one hour, before going to the next load level. Therefore, the results are not directly comparable to compression results, as crack could propagate during the load plateau. One of the cracks

[4] See RD02 - Lithoz – Regolith Compression Testing – RS0003855
[5] See RD03 - RS0004277 - Lithoz – Compression Testing of Lunar Regolith Simulant JSC-2A specimens

This document may not be reproduced, except in full, without the written permission of the European Space Agency.

was observed at the vicinity of a large porosity defect, which could indicate the influence of such defects on the crack initiation. Defects were also observed to have an effect on the crack propagation, as several defects (both pores and higher density inclusions) were found on the path of the cracks. This is linked to the stress concentrations around the defects, which tends to help the crack initiation and propagation.

The top and bottom surfaces of the samples being non planar and non-parallel (see Figure 4), it most probably caused some stress concentrations, leading to crack initiation and ultimately, failure of the corner (seen in Figure 12). A corner failure results in a smaller surface available to withstand the load, leading to a higher stress. The observed corner crack happened at a stress of 4.8MPa, which corresponds exactly to the plateau observed in the Compression test results (see Figure 1). This indicates that the observed plateau was associated to the breakage of the specimen corners at a constant load. The sudden load increase after the plateau is considered to be caused by the plates coming into contact with the remaining un-broken part of the sample.

7 CONCLUSIONS AND RECOMMENDATIONS

Even if the measured Ultimate Compressive Strength values presented a relatively high standard deviation (26.5%), they were significantly higher than what was measured on untreated JSC-2A Lunar Regolith Simulant specimens (3.33 ± 0.39 [MPa]) and untreated EAC-1 Lunar Regolith Simulant specimens (5.41 ± 0.29 [MPa]). This indicates that iron enriching of Lunar Regolith simulant JSC-2A by bacterial treatment can lead to a strong increase in the Ultimate Compressive Strength of structural material manufactured from the simulant.

Very large defects were found in the sample, which affected the crack propagation and therefore the compressive test results. Those defects were most probably due to the small amount of available powder feedstock, which prevented a fine milling. This led to coarser particles and agglomerates being fed into the additive manufacturing process, which caused difficulties in spreading the powder-binder suspension during the process. Processing more material could lead to a finer powder, which could improve at the same time the geometry of the samples (no distortion causing stress-concentrations) and reduce the amount and size of the defects. These two effects could potentially increase the Ultimate Compressive Strength even further.

This document may not be reproduced, except in full, without the written permission of the European Space Agency.

8 REFERENCES

[1] G. Cesaretti, E. Dini, X. De Kestelier, V. Colla and L. Pambaguian, "Building components for an outpost on the Lunar soil by means of a novel 3D printing technology," *Acta Astronautica*, vol. 93, pp. 430-450, 2014.

[2] S. L. Taylor, A. E. Jakus, K. D. Koube, A. J. Ibeh, N. R. Geisendorfer, R. N. Shah and D. C. Dunand, "Sintering of micro-trusses created by extrusion-3D-printing of lunar regolith inks," *Acta Astronautica*, vol. 143, pp. 1-8, 2018.

[3] A. Meurisse, A. Makaya, C. Willsch and M. Sperl, "Solar 3D printing of lunar regolith," *Acta Astronautica*, vol. 152, pp. 800-10, 2018.

9 APPENDICES

9.1 Incoming Inspection

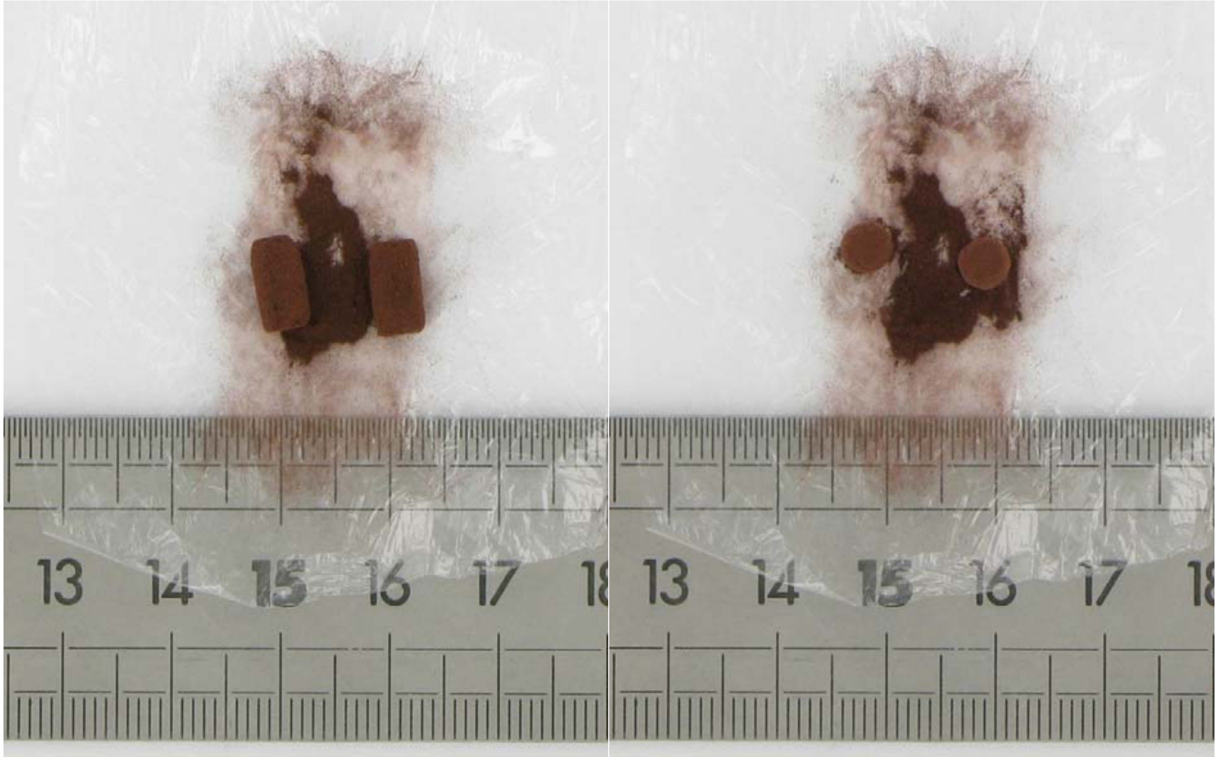

Figure 2: Side view (left) and top view (right) of as received specimens #1 and #2 (sintered at 1050ºC)

This document may not be reproduced, except in full, without the written permission of the European Space Agency.

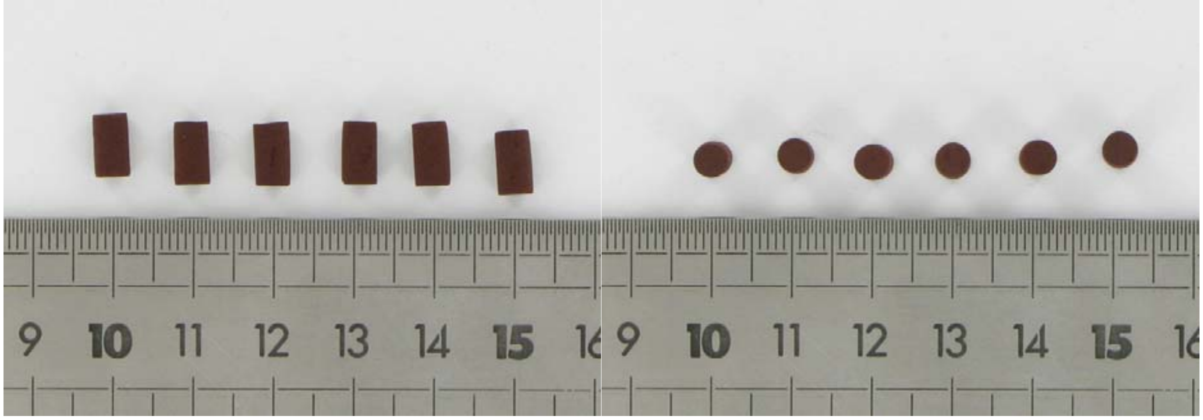

Figure 3: Side view (left) and top view (right) of as-received specimens 3 to 8 (sintered at 1100ºC)

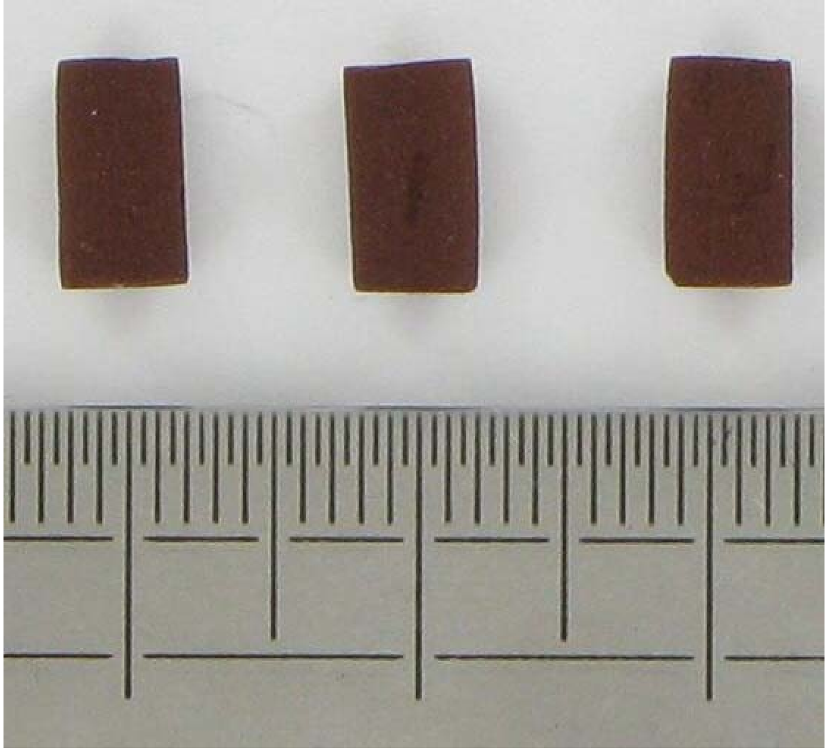

Figure 4: Zoom of Figure 3, showing the distortion of the samples

This document may not be reproduced, except in full, without the written permission of the European Space Agency.

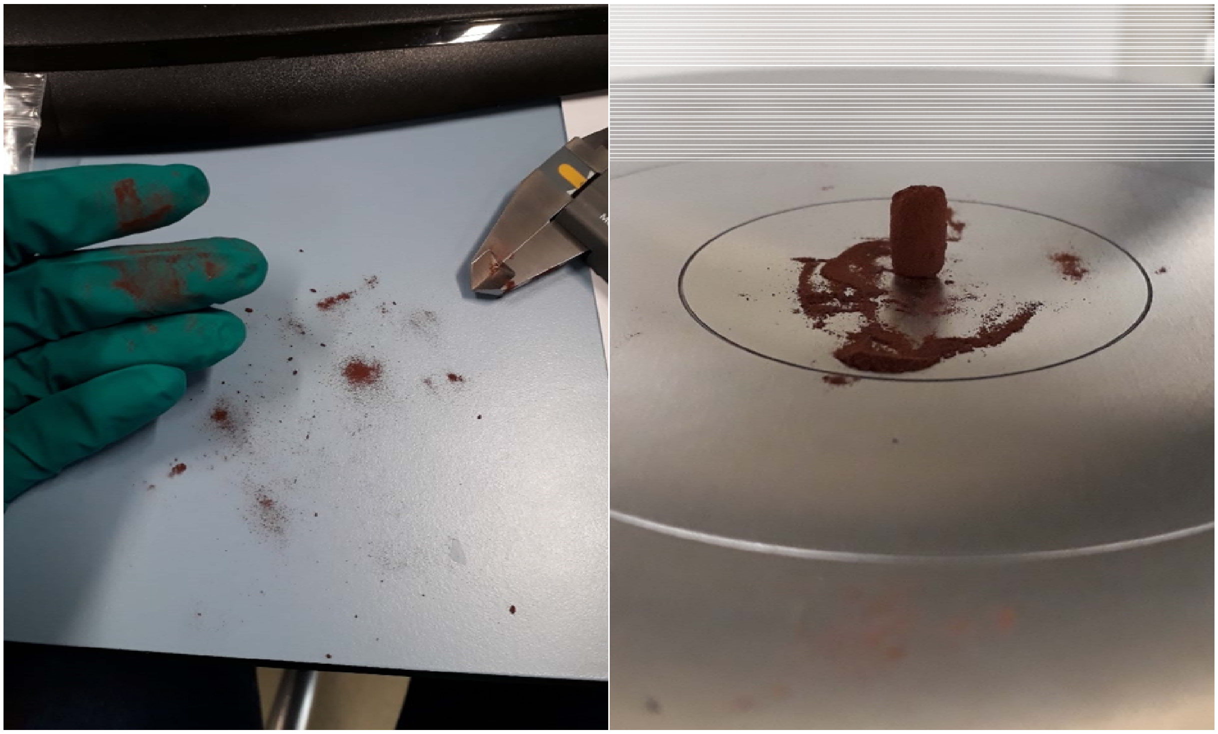

Figure 5: Deteriorations of sample #2 when carefully measuring its dimensions (left) and when placing between the compression plates (right)

This document may not be reproduced, except in full, without the written permission of the European Space Agency.

9.2 CT

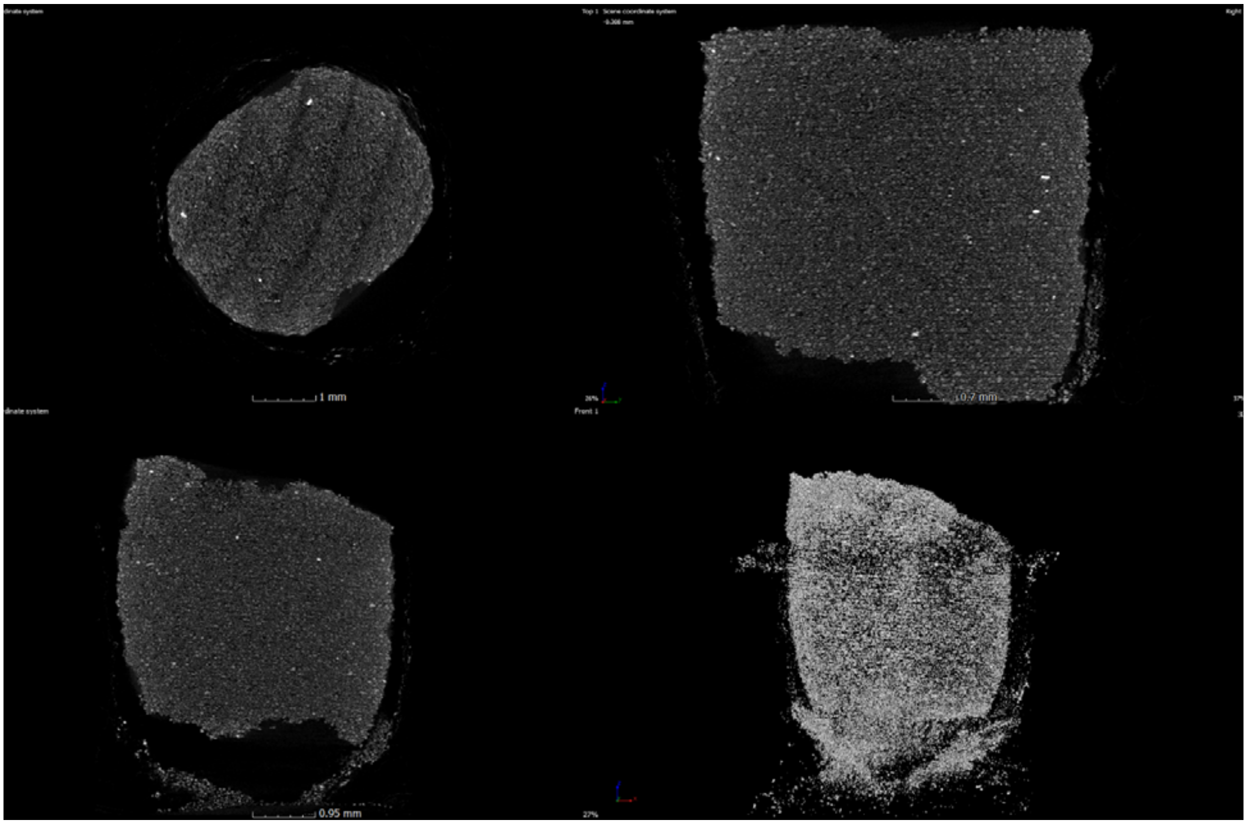

Figure 6: CT sections, overview of a part of sample #1

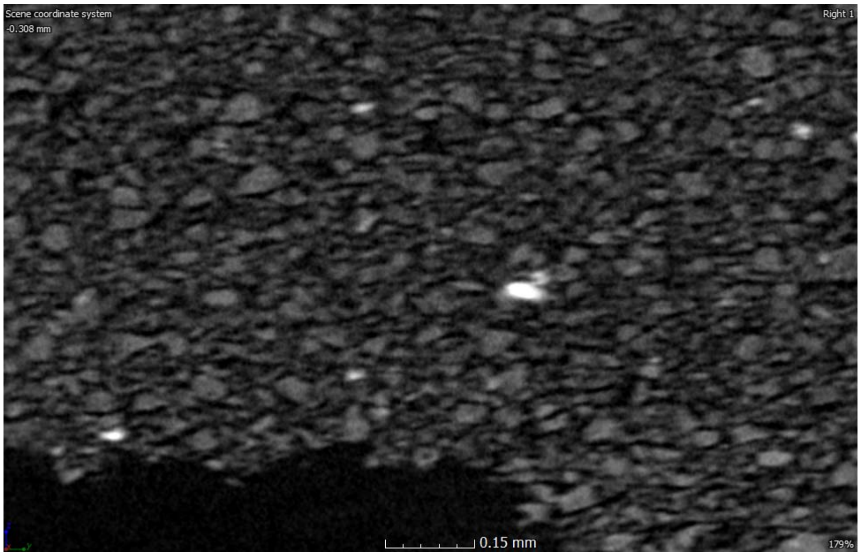

Figure 7: CT section, detailed view of sample #1

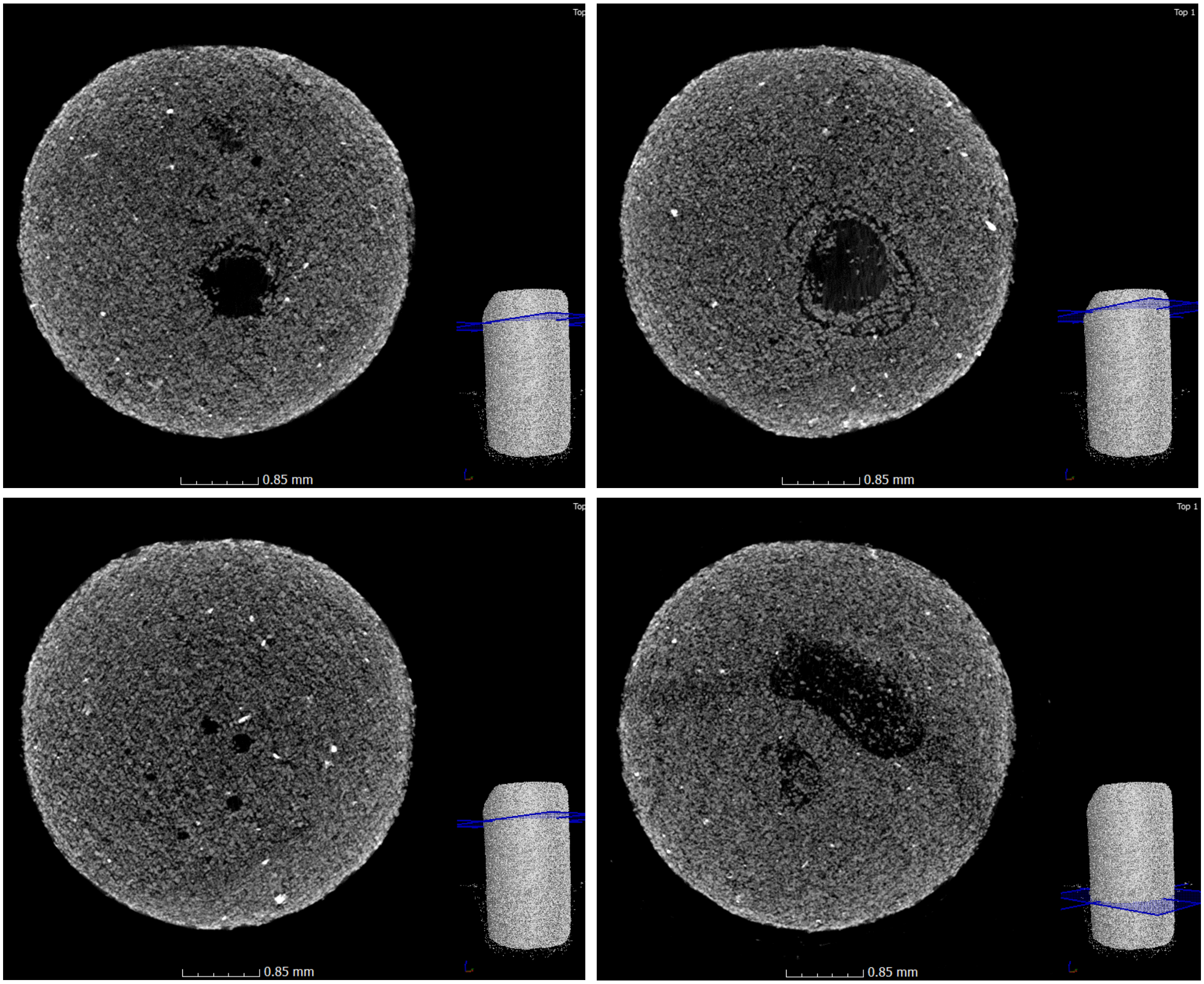

Figure 8: CT sections, top view of sample #3, in four different planes

This document may not be reproduced, except in full, without the written permission of the European Space Agency.

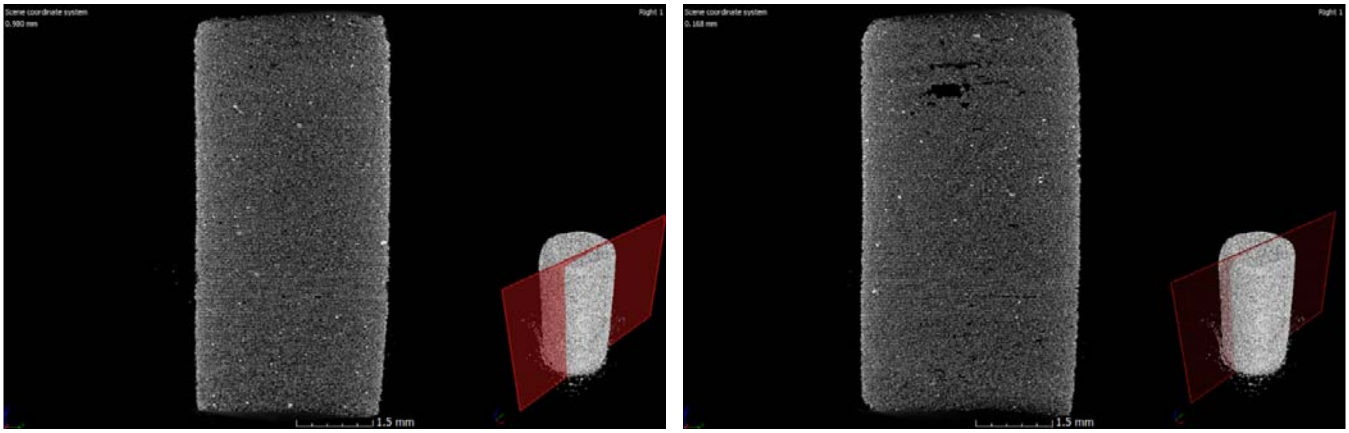

Figure 9: CT sections, side view of sample #3, in two different planes: peripheral plane (left) and central plane (right)

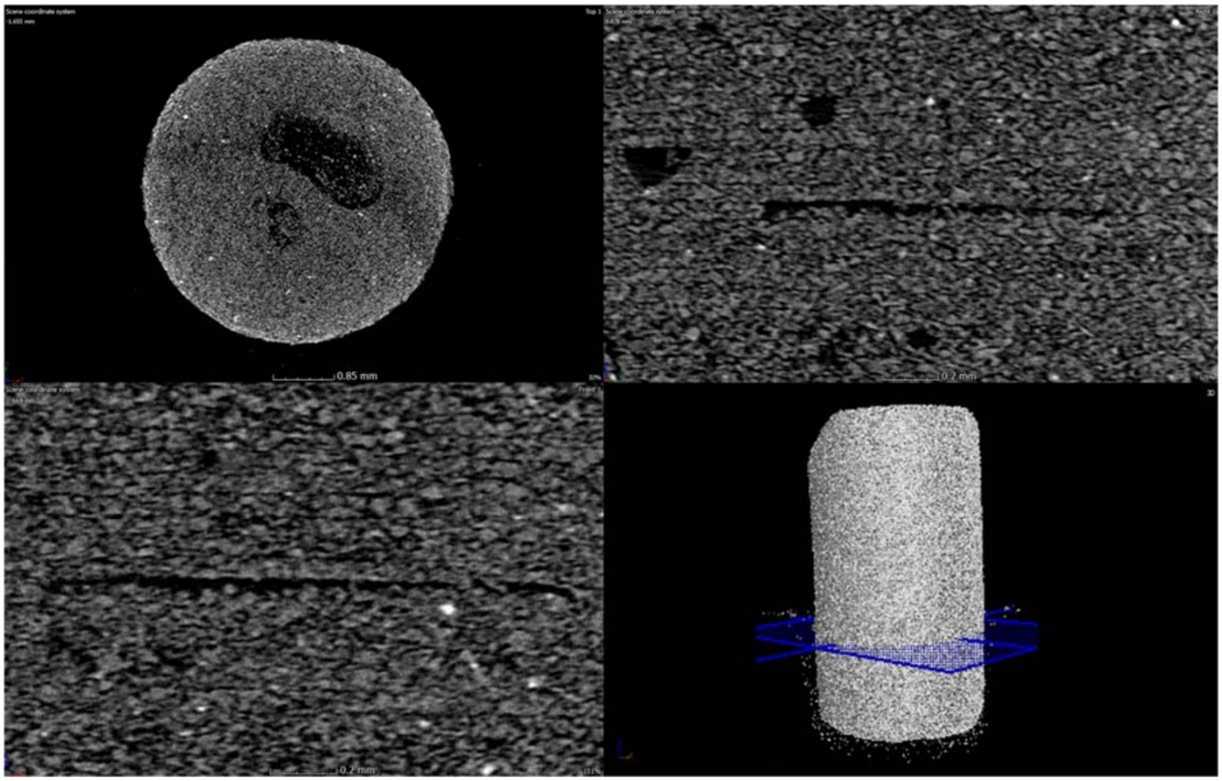

Figure 10: CT sections of sample 3, showing a delamination

This document may not be reproduced, except in full, without the written permission of the European Space Agency.

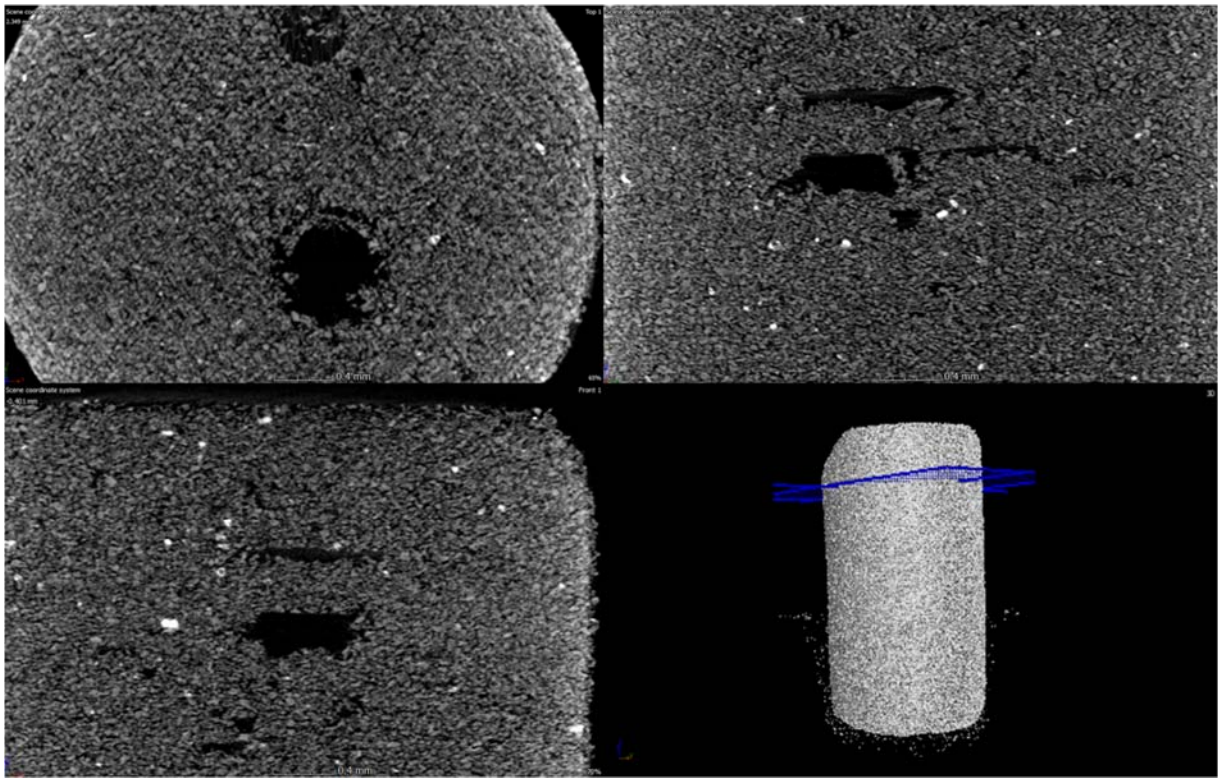

Figure 11: CT sections of sample #3 from top, right and front view, showing a defect cluster

This document may not be reproduced, except in full, without the written permission of the European Space Agency.

9.3 Compression-CT

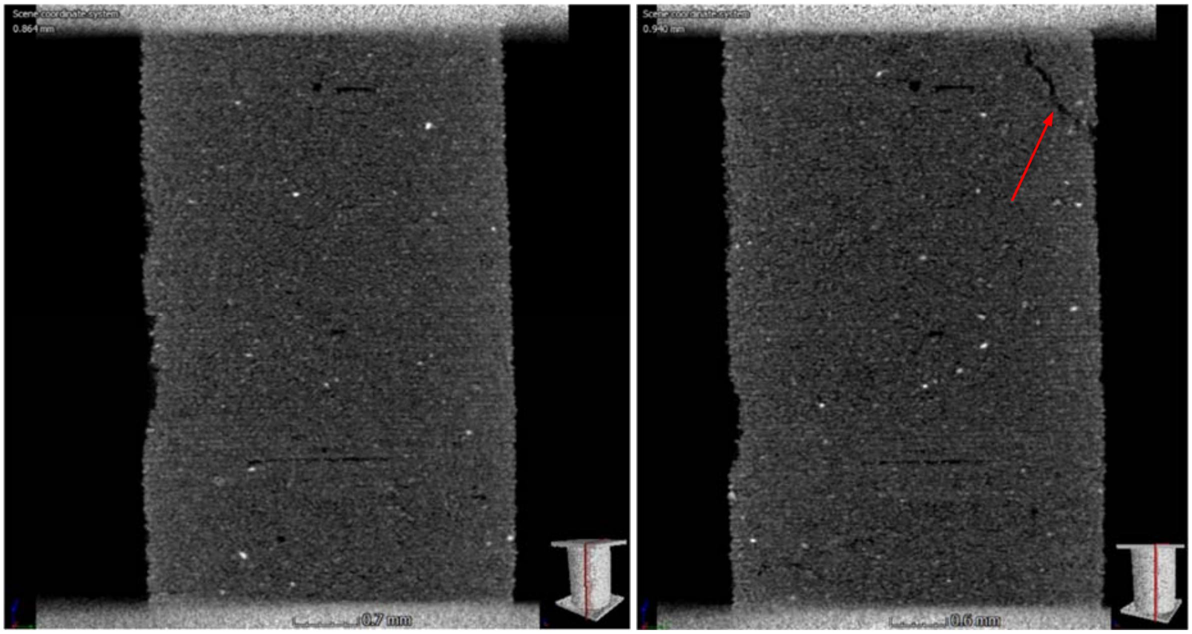

Figure 12: CT scans of sample #3, initial crack formation (red arrow) between the 60N load (3.9MPa, left) and the 75N load (4.8MPa, right)

This document may not be reproduced, except in full, without the written permission of the European Space Agency.

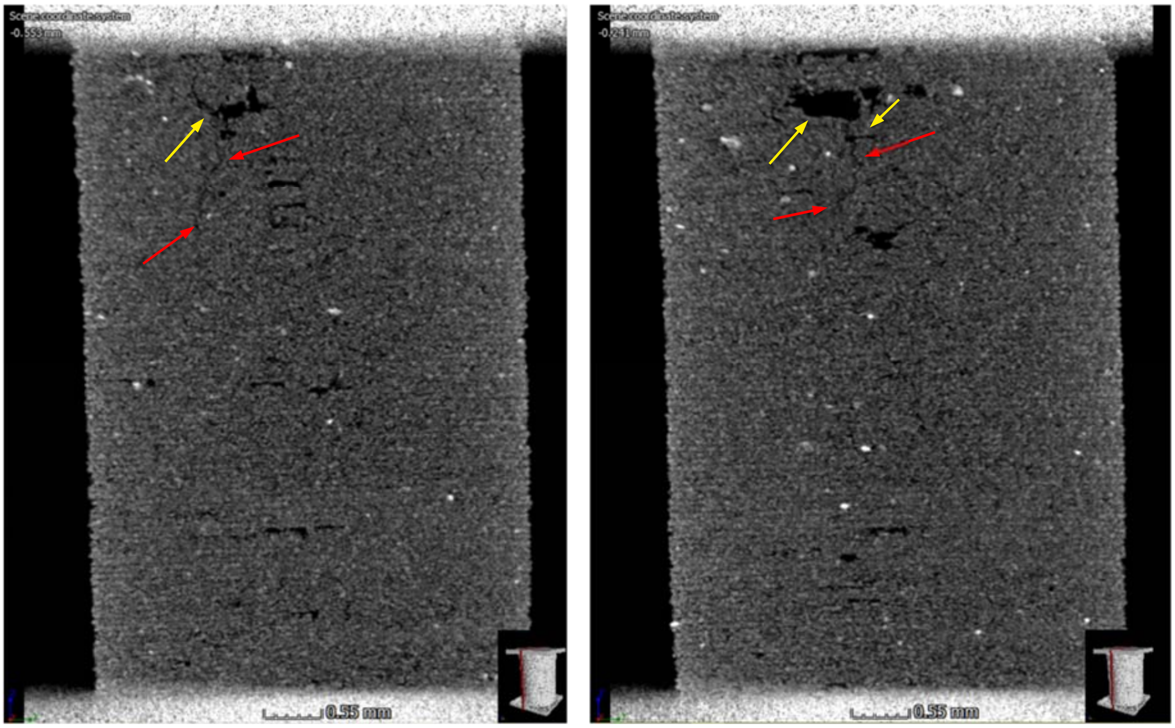

Figure 13: CT scans of sample #3 (side view), 2nd crack formation (red arrows) at the 105N load (6.8MPa), passing through initial defects (yellow arrows)

This document may not be reproduced, except in full, without the written permission of the European Space Agency.

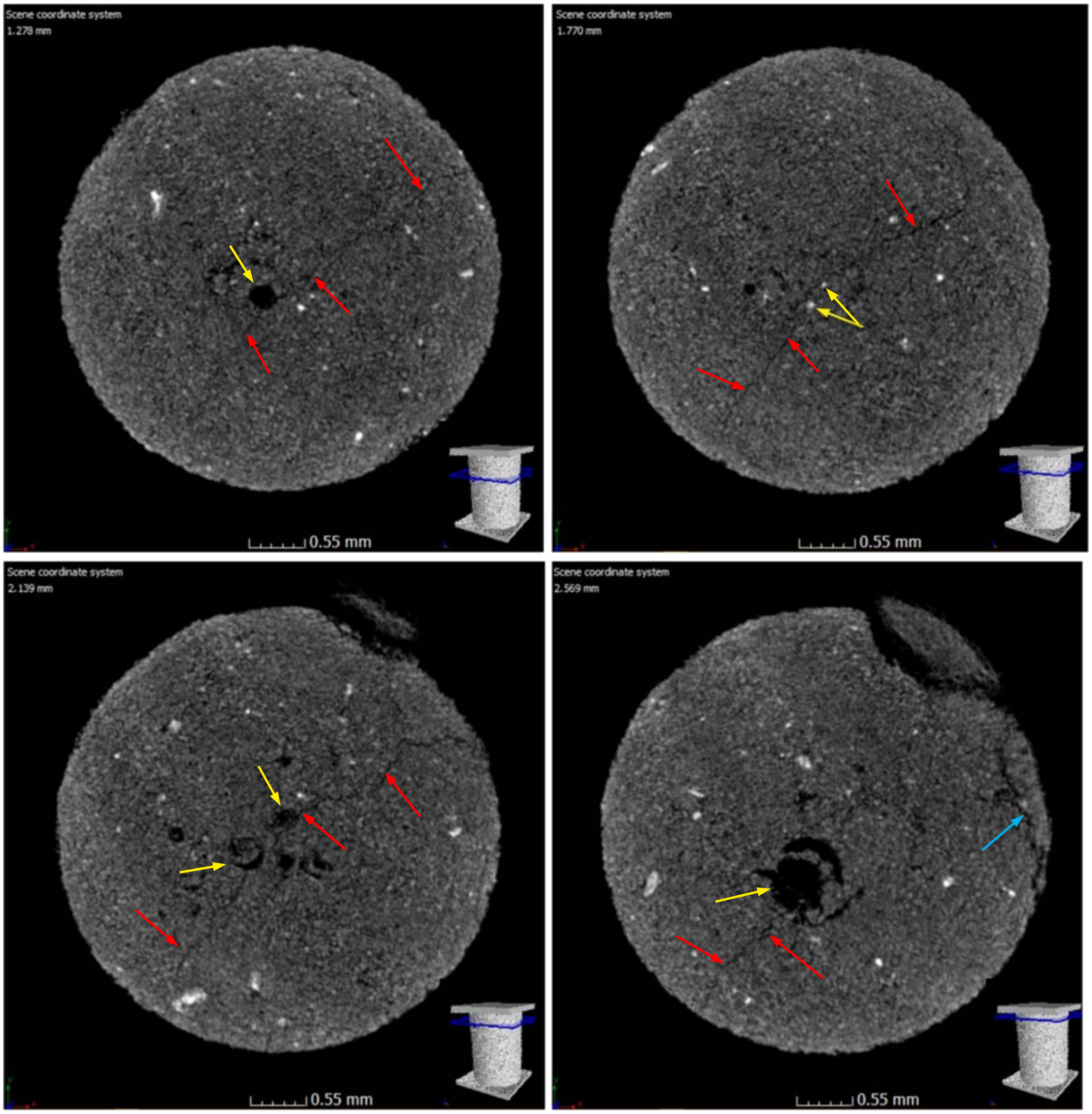

Figure 14: CT scans (top view) of different planes of sample #3, 2nd crack formation (red arrows) at the 105N load (6.8MPa), passing through initial porosity defects/inclusions (yellow arrows). The first crack (see Figure 12) lead to the formation of a third crack, indicated by the blue arrow

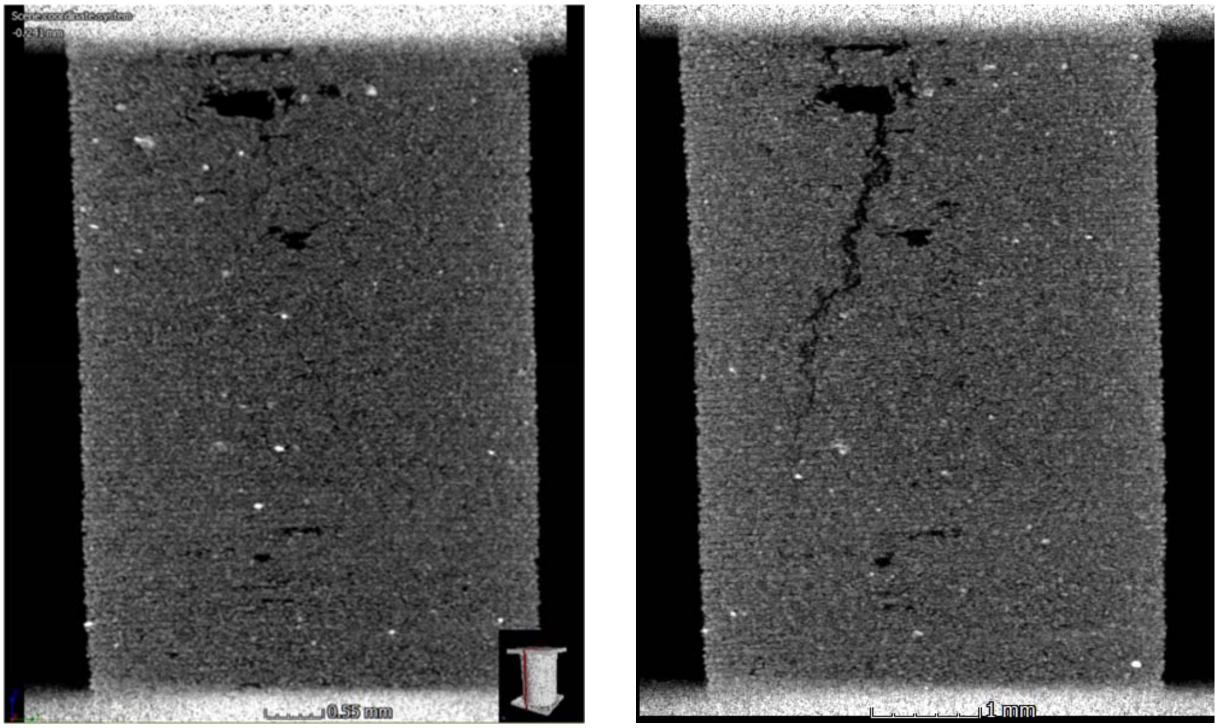

Figure 15: CT scans of sample #3 (side view), evolution of the main crack propagation between: left, the 105N load (6.8MPa) and right, the 120N load (7.7MPa)

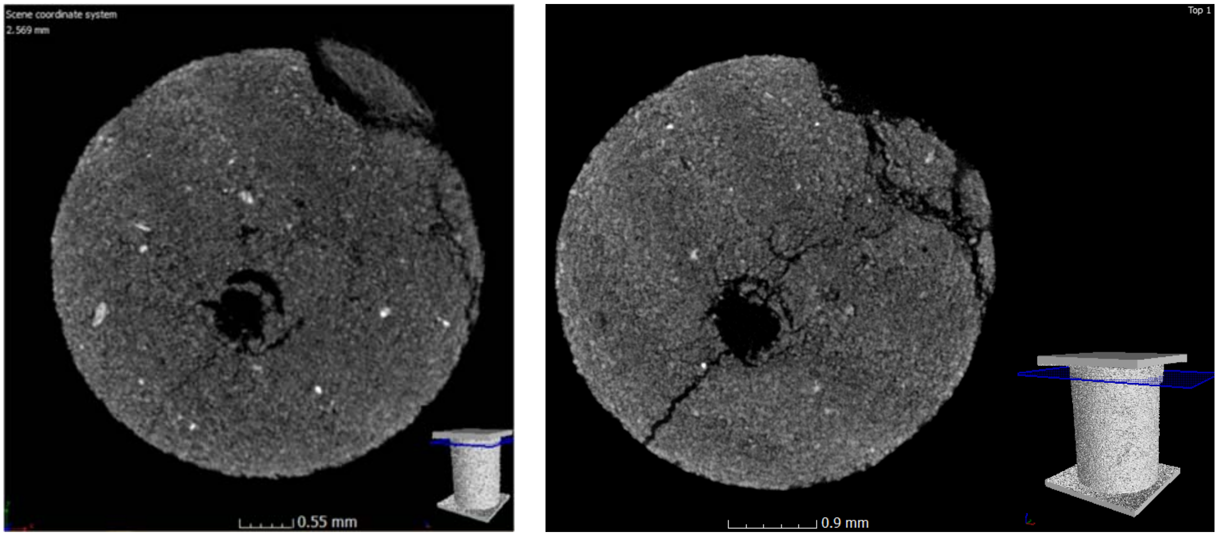

Figure 16: CT scans of sample #3 (top view), evolution of the propagation of the 3 different cracks between: left, the 105N load (6.8MPa) and right, the 120N load (7.7MPa)

This document may not be reproduced, except in full, without the written permission of the European Space Agency.

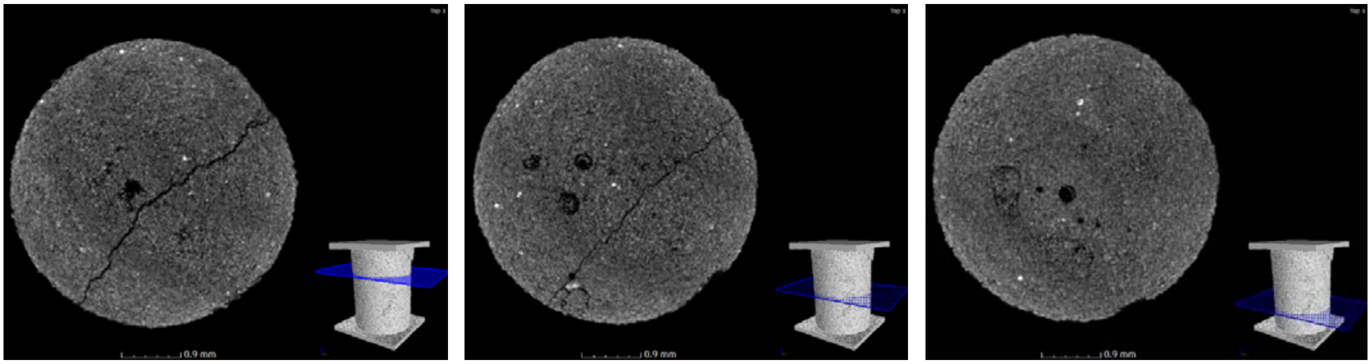

Figure 17: CT scans of sample #3 (top view), main crack in different sections, 120N load (7.7MPa). The crack did not reach the bottom of the specimen

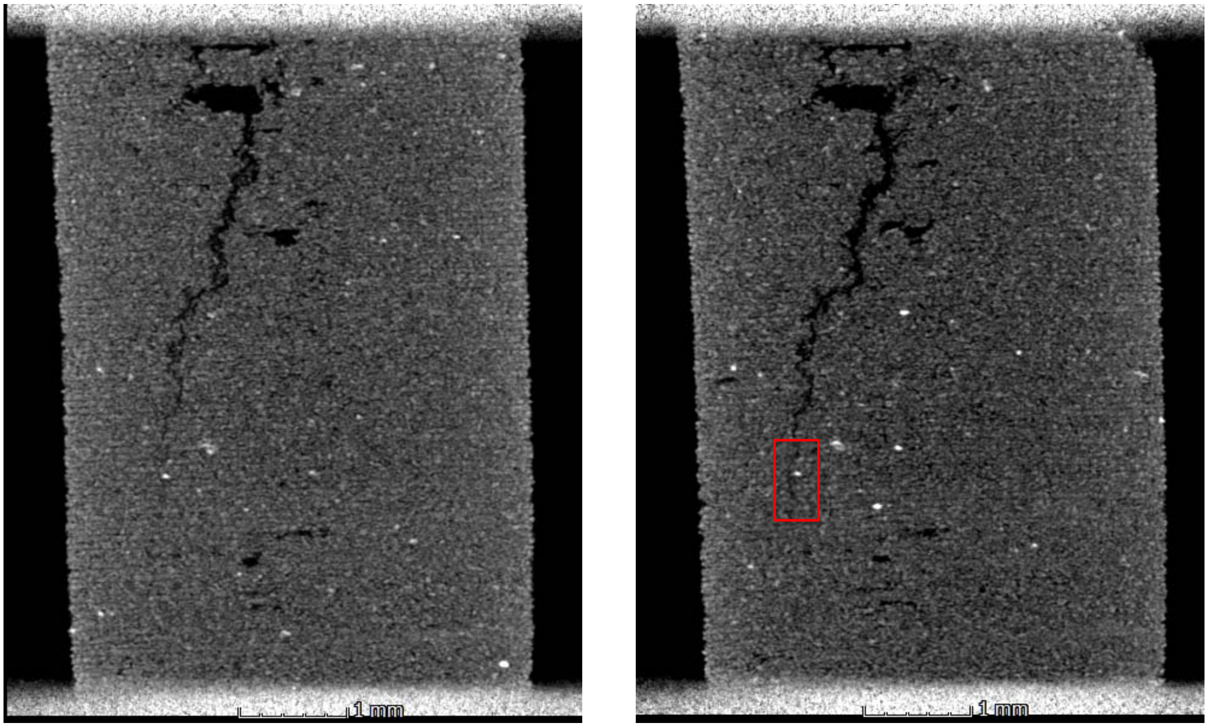

Figure 18: CT scans of sample #3 (side view), evolution of the main crack propagation between: left, the 120N load (7.7MPa) and right, the 135N load (8.7MPa). The crack grew wider and slightly longer (highlighted with the red box)

This document may not be reproduced, except in full, without the written permission of the European Space Agency.

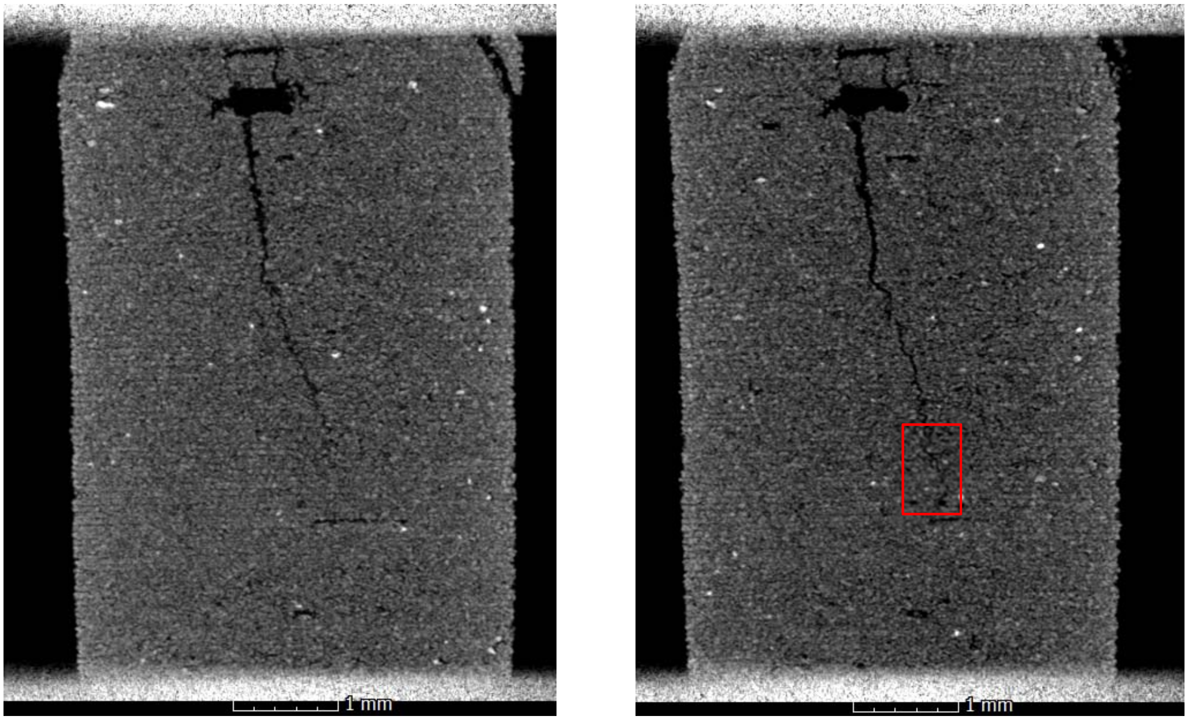

Figure 19: CT scans of sample #3 (front view), evolution of the main crack propagation between: left, the 120N load (7.7MPa) and right, the 135N load (8.7MPa). The crack grew wider and slightly longer (highlighted with the red box)

This document may not be reproduced, except in full, without the written permission of the European Space Agency.

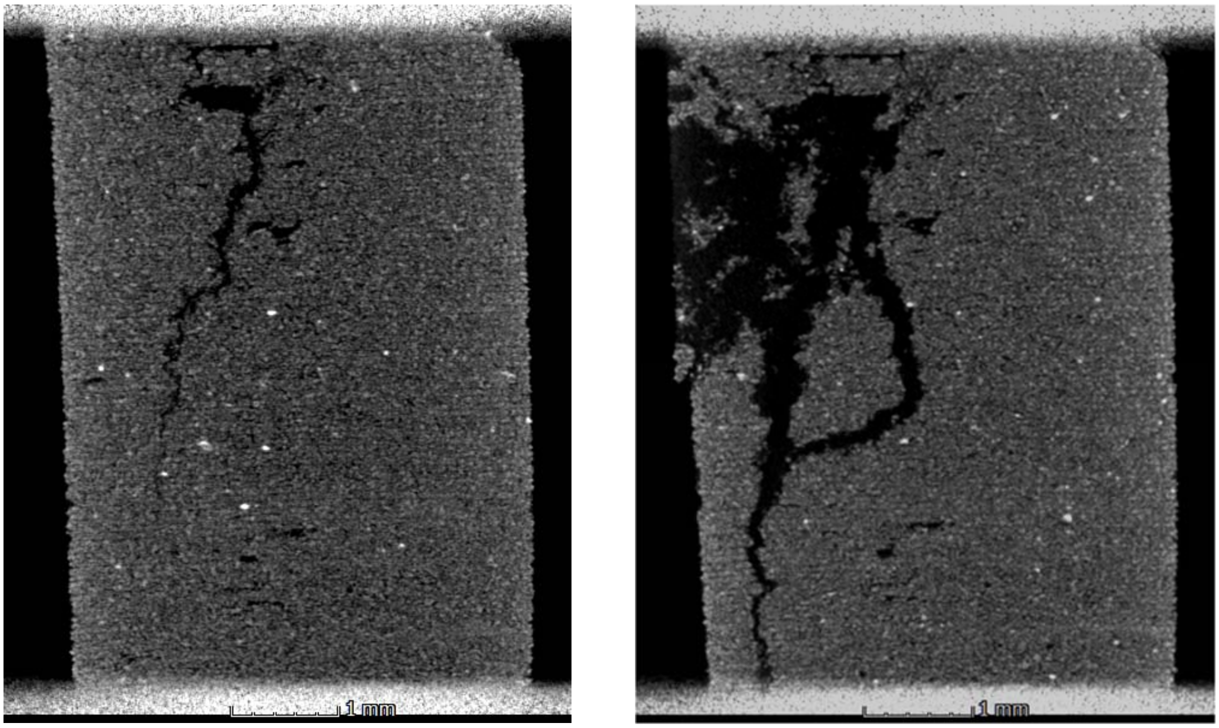

Figure 20: CT scans of sample #3 (side view), evolution of the main crack propagation between: left, the 135N load (8.7MPa) and right, the 150N load (9.7MPa). The crack grew until the bottom of the specimen, resulting in its failure

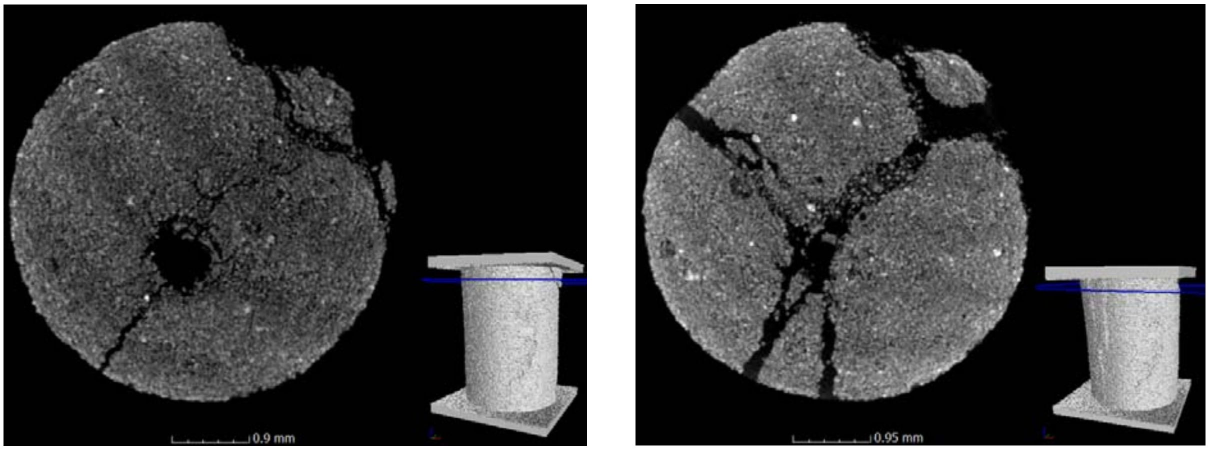

Figure 21: CT scans of sample #3 (top view), evolution of the main crack propagation between: left, the 135N load (8.7MPa) and right, the 150N load (9.7MPa). The initial defects of the sample collapsed and some sub-cracks appeared

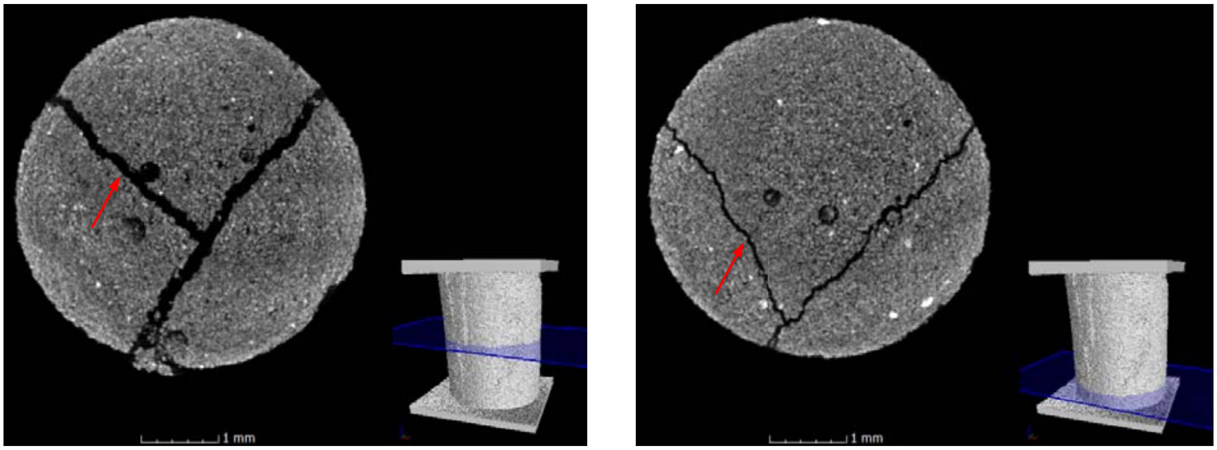

Figure 22: CT scans of sample #3 (top view); some sub-cracks (indicated by red arrows) appeared at failure (9.7MPa)

This document may not be reproduced, except in full, without the written permission of the European Space Agency.
