# Supplementary material for: Iron can be microbially extracted from Lunar and Martian regolith simulants and 3D printed into tough structural materials
Source: PLoS One. 2021 Apr 28;16(4):e0249962. doi: 10.1371/journal.pone.0249962 (PMC8081250; doi:10.1371/journal.pone.0249962)
Supplement: Attachment — Submitted filename: PLOS-Peer-Review-Report-Dec2020.docx [file pone.0249962.s002.docx]

I enjoyed reading this report and found can the potential this work has to contribute to ISRU. From the perspective as a reviewer the report found that Shewanella has potential as a practical tool for biomining and extraction of useful materials, in this case iron from Martian regolith, and even improves the quality of materials for use. This is particularly useful given that bacteria are less cumbersome to bring to Mars than heavy equipment and the work here provides a proof of concept level of work contributing to the preliminary foundation that this technology can be applied to space exploration. I thought overall the authors did well at summarizing and setting up the topic in abstract and introduction providing adequate context for why the work is significant, though there are some specific points here that may need clarification or correction (see below) overall the work is well set up in this aspect.

Some of the major areas for improvement generally fall under the following: 1. Organization, clarification and justification in the methods/results, 2. Clarification and correction of a some important points discussed in the introduction, results and discussion sections, and 3. Important limitations that may need to be additionally discussed. More details of this are provided below; I will try to be specific about where such instances occurred, however it would be helpful in the future to include line numbers/page numbers to assist in the process.

1. Organization, clarification, and justification in the methods/results

- Organization of the methods is a little hard to follow mostly because some of the details of the methods are found in (and in some cases repeated in) the results. For example, in the Results Section under the heading “Toxicity of Regolith Simulants…” the methods for establishing toxicity are repeated with details that would be more appropriate for the methods section. This also occurs in the “Magnetic extraction and Fe(II) content” section, the “iron and silicon concentration…”. Sometimes the information is simply repeated, but in some instances the information is more detailed in the results and lacks in the methods. I also had trouble locating information on the Preparation of aerobic samples in the methods section, though there were some details in the results. There is a detailed section on the preparation of anaerobic samples, but no equivalent for aerobic samples which would be necessary to reproduce the results. The details for the methods are there for the most part, but they need to be reorganized to be focused on within the methods section. It may be appropriate to discuss or justify specific aspects within in the results or discussion sections, but the actual procedural details would be easier for the reader to find and follow if organized together in the methods section.
- I did not fully understand why lunar simulants were being used to test materials on Mars. Technically each of these simulants are produced from terrestrial basalts, so they all originate on Earth. The only information that justified the use of lunar simulants in a Mars application was that they were chemically similar, but again if that is the case then technically any similar basaltic material could be applied. Was the there a need because additional Martian simulants were not available, was there a desire to also test potential for building structures on the Moon? Was there another reason (other than being chemically similar) that lunar simulants were used as apposed to using multiple mars simulants or just basaltic material in general. What made the lunar simulants more applicable to Mars than a general basaltic material might, especially as focus of the study was Mars? This just needs to be briefly justified.
- Throughout the paper you refer to a total iron concentration, but it is not clear where this iron is coming from. Is Schewanella literally making more atoms of iron (not likely, but the way it is worded know lends to being interpreted that way)? Or is this iron specifically Fe(II), or total aqueous iron, or is this total iron concentration specifically in the metallically extracted material. Just need to clarify what type and how this increase in iron concentration is measured, especially the first few times mentioned. Some specific examples of where this occurred are in the introduction (near the end shortly before the results and discussion) and then multiple instances in the results section. To help clarify this it may be useful to include the metabolic pathway for Schewanella (a simplified one would be adequate) to show the iron transformations taking place.

2. Clarification and correction of some important points discussed in the introduction, results and discussion sections. There are few instances of overstating or misstating a few important points important for establishing context.

- In the results and discussion section under the heading of “composition and limitations of regolith simulants”. The following statements may need clarification or correction
  - “The composition on Martian soil varies between different locations…”
    - Actually, from what we have measured, modern Mars soil has a consistent composition at most locations. There are a few areas where locally the composition is different because of hydrothermal alteration or a different parent material, but the vast majority of Mars modern soil is very similar on a global scale. The bedrock and other surface materials differ quite a bit, but the modern soil does not. But that being said, it is still useful to rest different scenarios as surface materials other than soil could be useful to explore.
  - “JSC-Mars1…is very young compared to the Lunar simulants…few to no environmental alterations are expected. Notably actual Martian regolith contains SO3…”
    - What do you specially mean by environmental alteration, so you mean anthropogenic influences or that the material has not been changed much from the original parent material. The former would require a source to confirm, the latter is not accurate. As you state previously this simulant is composed of palagonitized tephra ash. Plagaotinization is an alteration process. It is basically altered basaltic glass, altered from weathering. Although some of the parent minerals are still present, much of the JSC-Mars-1 simulant is composed of secondary materials (many of the x-ray amorphous materials) produced by weathering. In this case, age alone, but also composition, are the necessary indicator for how altered the sample is. In fact, the majority of the Fe(III) in the simulant are likely sourced from weathered material in the simulant, i.e. it has an abundance Fe(III) likely because it has had greater degree of weathering/alteration. (The same is true for Fe(III) on Mars so again this does not invalidate anything, just an important piece of context that should be addressed)
    - Mars regolith actually contains SO4 (sulfate), but when in-situ samples are processed in the rover instruments it is volatilized to SO3, so that is what the instruments report it as. Similar to how other oxides are reported. But Mars soil contains sulfate (SO4)

3. Important limitations that may need to be additionally discussed

- The JSC-Mars-1 is a spectral simulant specifically, and so has spectral characteristics important comparable to Mars. At a proof of concept level it is an appropriate simulant for the purposes you intend and does well at supporting the claims. As the work progressed beyond a proof of concept, there are some important limitations of this simulant, including in the magnetic properties, that may be important to mention here. The Allen et al. 1998 reference does include some of the information, Cannon et al. 2019 (Mars global simulant MGS-1) also may have additional helpful information. The simulant has a large amount of x-ray amorphous phases that contribute to the magnetic properties and to mineralogical and chemical differences from actual Martian soil and regolith and these limitations are important to consider as this and similar work develop. Mentioning the limitations may help bring strength to your own work and provide important information for those seeking to duplicate or continue development of biomining on Mars. Again, for the proof of concept level you are demonstrating it is very useful and provides important insights, but somewhere in the discussion important to consider limitations specifically related to magnetism and whether the magnetic properties of the simulants reflects that of Mars soil.

The remaining comments for revision focus on smaller scale items rather than items that affect multiple sections, so I will simply go through these in order as they occur in the paper and try to indicate specifically the location and the issue that needs to be addressed.

- As a reader, I was a little confused at first to which simulants exactly are being used. This is not specified until further into the paper. It may be useful to indicate this information earlier in the introduction and provide the references for which details on the development or obtaining of the simulants can be found. I would also suggest an additional reference the JSC-Mars simulant may have (Allen et al. 1998 MARTIAN REGOLITH SIMULANT JSC MARS-1.). This is the conference abstract that describe the simulants development and origins.

The abstract

- “Application of self-reproducing bacteria…is a promising approach to avoid the pitfalls”
  - The word avoid may be a slight overstatement. Bacteria would certainly require less cumbersome equipment, but they do still require equipment to maintain and house them and apply them (for example the 3D printers you mention). It would not eliminate the use of heavy or cumbersome equipment but would certainly help to lessen it.
- “resulting in a 396 +- 115% improvement…”
  - That seems like a very large error range. If this is not a typo, the source of that error range and what that might mean about the results would be important to mention in the discussion

The Introduction

- The paragraph that starts “Traditionally, research in the sector”.
  - I am not sure the purpose this paragraph serves in the overall discussion. As a reader it seemed random and unnecessary. I recommend either deleting or providing transition that helps it fit into the context.
- “These organisms can be produced in large quantities, only requiring water…growth medium.”
  - They also require the equipment needed to house them in (glassware or whatnot), much smaller than the factories and machinery previously mentioned, but still an important piece needed.
- “Microbial production…do not require…high energy investment or toxic chemical”
  - Some may require toxic chemicals, and some actually produce them. That depends on the microorganism being used. They do decrease the energy and the toxic and have huge potential as you state, but the way the current sentence is phrased may be a bit of an overstatement.
- “Together, our findings provide the first…”
  - This is an important point, but the sentence is rather long and complex so the point becomes a little muddled. Perhaps break it into multiple sentences

Results and Discussion

“Composition and limitations of regolith simulants”

- “whereas EAC-1 mined from a deposit…EAC-1 shows alterations…decided to use JSC-2A…in our experiments
  - This paragraph of discussion was a little confusing. It tends to go back and forth between the two simulants quite a bit and it is hard to follow as a reader the logic used in choosing among the simulants and why EAC-1 was used if it had alteration while at the same time JSC-2A was used to avoid alteration in JSC-1A. Perhaps a little reorganization and clarification of this discussion.
- Table 2: There are a few minerals indicated as only present in the magnetically extracted material. Were these minerals after exposure to Schewnella so only present because of treatment, or are these minerals present in the original samples. The tables purpose seems to be to present the original composition so if these minerals are a result of treatment, would they be appropriate to indicate here?

“Toxicity of Regolith simulants in aerobic and anaerobic conditions”

- “This result may reflect the difficulty in obtaining reliable absorbance measurements in the presence of high iron concentrations due to optical properties of iron”
  - Is this difficult specific to iron (II) or iron (III). Also what would allow you to differentiate that this is the cause as apposed to another reason? Stating this will help to support the interpretation of the results.

“Iron and Silicon concentration in the magnetically extracted materials”

- Important to clarify which part of the sample you are referring to here. It is unclear if the whole sample, just the extracted portion, or the unextracted portion. As you discuss these results, clarify more clearly which portion a particular result refers to. For example the XRF was it performed on the entire sample or on just the extracted portion or the untreated sample? It can become confusing exactly where extra iron is coming from and silica is disappearing to, but being more specific could help to clarify.

Conclusion

- The conclusions overall go back and forth a lot, particularly noticed in the second paragraph of the section “Bacterial reduction of regolith simulants”. As a reader the order (or lack of) made the topic of discussion is hard to follow, perhaps some reorganization of the information would help.

Materials and Methods

- Toxicity of regolith simulants
  - “previously sterilized regolith samples were sterilized again via autoclaving and diluted to the desired concentration…”
    - What was the “previous sterilization” process that was used. Also what temperature of autoclaving. If heated high enough this can affect the iron oxides in the simulant, for example goethite converts to hematite. They are still a source of Fe(III) and Shewnella is very adaptive and can use a variety of different Fe(III) sources, but the kinetics does differ among so can have important affect on the results. Be more specific about how they were sterilized, especially temperature or exposure to oxidizing materials like hydrogen peroxide if used.
- Magnetic Extraction apparatus
  - Had a hard time picturing what this looks like. May be useful to include a simple schematic or image of the device. Not essential, but if there is ability to include a figure may be useful.
- Lithography-baed ceramic printing
  - “dried and sintered for 2 hours at 1050C and 1100C for the untreated and treated samples, respectively”
    - Why different temperatures, and would this affect the strength of the material? Important to clarify.

Other comments

- Some additional references that could be useful that are not cited here include Cockell 2010 and 2011 about Geobiology in space. The author presents some interesting discussion of biomining and bio-extraction in ISRU applications of space exploration. Considering the topic, are important references to consider.
- Overall a very interesting study with potential to contribute very significant work to ISRU applications. There are some major revision needed before ready for publication, but can tell it will be a significant contribution to the literature.
