# Supplementary material for: Iron can be microbially extracted from Lunar and Martian regolith simulants and 3D printed into tough structural materials
Source: PLoS One. 2021 Apr 28;16(4):e0249962. doi: 10.1371/journal.pone.0249962 (PMC8081250; doi:10.1371/journal.pone.0249962)
Supplement: Attachment — Submitted filename: Response to reviewers.docx [file pone.0249962.s003.docx]

*PLOS ONE*

Dear Editorial office,

Thank you for sending us the referee comments for our revised manuscript, “Iron can be microbially extracted from Lunar and Martian regolith simulants and 3D printed into tough structural materials” submitted by Sofie M. Castelein, Tom F. Aarts, Juergen Schleppi, Ruud Hendrikx, Amarante J. Böttger, Dominik Benz, Maude Marechal, Advenit Makaya, Stan J. Brouns, Martin Schwentenwein, Anne S. Meyer, and Benjamin A.E. Lehner.

In response to the comments of the reviewers, we have improved our manuscript. All changes are indicated in blue in the marked-up version of the manuscript. They are also described in a point-by-point fashion on the subsequent pages. We are confident that we have addressed the final referee’s concerns appropriately, and we are hopeful that you will consider our manuscript suitable for publication in *PLOS Online*.

The company Lithoz provided support in the form of salaries for M. Schwentenwein and F. Ertl as well as access to their 3D printing facility, but did not have any additional role in the study design, data analysis, decision to publish, or preparation of the manuscript. The specific roles of these authors are articulated in the ‘author contributions’ section. All authors contributed to the writing and preparation of the manuscript. This does not alter our adherence to PLOS ONE policies on sharing data and materials. Please do not hesitate to contact us with any further questions. We look forward to hearing from you soon.

Best Regards,

Dr. Benjamin A.E. Lehner	Dr. Anne S. Meyer

**Reviewer 1:**

*“I enjoyed reading this report and found can the potential this work has to contribute to ISRU. From the perspective as a reviewer the report found that Shewanella has potential as a practical tool for biomining and extraction of useful materials, in this case iron from Martian regolith, and even improves the quality of materials for use. This is particularly useful given that bacteria are less cumbersome to bring to Mars than heavy equipment and the work here provides a proof of concept level of work contributing to the preliminary foundation that this technology can be applied to space exploration. I thought overall the authors did well at summarizing and setting up the topic in abstract and introduction providing adequate context for why the work is significant, though there are some specific points here that may need clarification or correction (see below) overall the work is well set up in this aspect.*

*Some of the major areas for improvement generally fall under the following: 1. Organization, clarification and justification in the methods/results, 2. Clarification and correction of a some important points discussed in the introduction, results and discussion sections, and 3. Important limitations that may need to be additionally discussed. More details of this are provided below; I will try to be specific about where such instances occurred, however it would be helpful in the future to include line numbers/page numbers to assist in the process.”*

1. *Organization, clarification, and justification in the methods/results*

- *Organization of the methods is a little hard to follow mostly because some of the details of the methods are found in (and in some cases repeated in) the results. For example, in the Results Section under the heading “Toxicity of Regolith Simulants…” the methods for establishing toxicity are repeated with details that would be more appropriate for the methods section. This also occurs in the “Magnetic extraction and Fe(II) content” section, the “iron and silicon concentration…”. Sometimes the information is simply repeated, but in some instances the information is more detailed in the results and lacks in the methods. I also had trouble locating information on the Preparation of aerobic samples in the methods section, though there were some details in the results. There is a detailed section on the preparation of anaerobic samples, but no equivalent for aerobic samples which would be necessary to reproduce the results. The details for the methods are there for the most part, but they need to be reorganized to be focused on within the methods section. It may be appropriate to discuss or justify specific aspects within in the results or discussion sections, but the actual procedural details would be easier for the reader to find and follow if organized together in the methods section.*

1a: We would like to thank reviewer 1 for the kind words about our manuscript. Methodological details, such as incubation times, regolith concentrations, observed timepoints, and methodology steps were removed from the “Results & Discussion” section and added to the “Materials & Methods” section when no justification of the methodological details was required (see marked changes in the manuscript). In the subsection “Magnetic extraction and Fe(II) content upon aerobic bacterial treatment”, the incubation time and regolith concentration are still indicated, since we believe it is relevant here to justify that an increased incubation time and relatively high regolith concentration is required to increase the quality and quantity of the extracted material.

1b: We have changed the subsection “Preparation of anaerobic samples” into a general subsection named “Sample preparation”. Here, it is described how the aerobic and anaerobic samples are prepared, as well as the additional steps required for the preparation of anaerobic samples. The preparation of defined minimal medium, which was initially described in the “Preparation of anaerobic samples” subsection, is now described in a separate subsection.

1c: An additional change was made in the “Materials & Methods” section to rename the “Magnetic extraction apparatus” subsection to “Small-scale magnetic extraction” (required for determining the amount of extracted material), to avoid confusion with the subsection “Large-scale magnetic extraction” (which were required for XRF and XRD experiments).

- *I did not fully understand why lunar simulants were being used to test materials on Mars. Technically each of these simulants are produced from terrestrial basalts, so they all originate on Earth. The only information that justified the use of lunar simulants in a Mars application was that they were chemically similar, but again if that is the case then technically any similar basaltic material could be applied. Was the there a need because additional Martian simulants were not available, was there a desire to also test potential for building structures on the Moon? Was there another reason (other than being chemically similar) that lunar simulants were used as apposed to using multiple mars simulants or just basaltic material in general. What made the lunar simulants more applicable to Mars than a general basaltic material might, especially as focus of the study was Mars? This just needs to be briefly justified.*

1d: Basaltic material differs strongly between different samples and suppliers. The biggest advantage of the lunar regolith simulant (especially JSC-2A) is the amount of research and standardization performed on it. This great benefit allows for increased replicability. The following sentence was added in the introduction: “In addition to a Martian regolith simulant, two Lunar regolith simulants were used (Table 1). These two Lunar regolith simulants are chemically similar to the Martian regolith simulant, and have the advantage that they have been, substantially studied and standardized compared to other basaltic materials [23].”

- *Throughout the paper you refer to a total iron concentration, but it is not clear where this iron is coming from. Is Schewanella literally making more atoms of iron (not likely, but the way it is worded know lends to being interpreted that way)? Or is this iron specifically Fe(II), or total aqueous iron, or is this total iron concentration specifically in the metallically extracted material. Just need to clarify what type and how this increase in iron concentration is measured, especially the first few times mentioned. Some specific examples of where this occurred are in the introduction (near the end shortly before the results and discussion) and then multiple instances in the results section. To help clarify this it may be useful to include the metabolic pathway for Schewanella (a simplified one would be adequate) to show the iron transformations taking place.*

1e. Shewanella does not produce iron atoms but increases both Fe(II) and aqueous iron concentration by using Fe(III) as a terminal electron acceptor in its metabolic pathway. The total increased iron concentration in the final sample is due to the magnetic extraction step. To improve clarity the following changes were made:

1) Abstract: “The quantity of bacterially extracted material was up to 5.8 times higher and the total iron concentration was up to 43.6% higher in comparison to untreated material” was changed to “The combination of bacterial treatment and magnetic extraction resulted in a 5.8-times higher quantity of iron and 43.6% higher iron concentration compared to solely magnetic extraction.”.
2) Introduction: To clarify the metabolic pathway, “Martian regolith contains a high concentration of iron in the form of Fe(III) [21] that can be reduced to magnetite by *S. oneidensis* [22], which can subsequently be extracted magnetically” was changed to “Martian regolith contains a high concentration of iron in the form of Fe(III) [21] that can be reduced to Fe(II) by *S. oneidensis*, promoting the precipitation of magnetite which can subsequently be extracted magnetically [22]”.
3) Introduction: “Anaerobic iron extraction of JSC-Mars1 regolith simulant with minimal growth medium resulted in a 5.8-fold increase in the quantity of magnetically extractable iron and a total iron concentration that was 13.6% higher than in the starting material” was changed to “The combination of bacterial treatment and magnetic extraction of JSC-Mars1 regolith simulant resulted in a 5.8-fold increase in the quantity of extracted material compared to material magnetically extracted from untreated samples. Furthermore, the total iron concentration of bacterially treated and magnetically extracted JSC-Mars1 was 18.2% higher than in the starting material.”
4) Results & Discussion, ”Magnetic extraction and Fe(II) content upon aerobic bacterial treatment”: In the original sentence “In order to produce more magnetic minerals within regolith and therefore concentrate the iron content,” “to” was replaced with “therefore” to make it clear that more magnetic minerals will lead to more iron content.
5) The word aqueous was added where applicable throughout the manuscript.
2. *Clarification and correction of some important points discussed in the introduction, results and discussion sections. There are few instances of overstating or misstating a few important points important for establishing context.*

- *In the results and discussion section under the heading of “composition and limitations of regolith simulants”. The following statements may need clarification or correction*
  - *“The composition on Martian soil varies between different locations…”*
    - *Actually, from what we have measured, modern Mars soil has a consistent composition at most locations. There are a few areas where locally the composition is different because of hydrothermal alteration or a different parent material, but the vast majority of Mars modern soil is very similar on a global scale. The bedrock and other surface materials differ quite a bit, but the modern soil does not. But that being said, it is still useful to rest different scenarios as surface materials other than soil could be useful to explore.*

2a. To accommodate the suggested change, we adapted the sentence from: “However, since the actual composition on Martian soil varies between different locations on the surfaces of the celestial objects, it is helpful to use a number of different regolith simulants with varying compositions to test for multiple different scenarios.” to the following: “However, the variation in Martian regolith composition due to hydrothermal activity and the differences in composition between Martian bedrock and other surface materials necessitates the use of several types of regolith with varying compositions to test for multiple different scenarios.”

- *“JSC-Mars1…is very young compared to the Lunar simulants…few to no environmental alterations are expected. Notably actual Martian regolith contains SO3…”*
  - *What do you specially mean by environmental alteration, so you mean anthropogenic influences or that the material has not been changed much from the original parent material. The former would require a source to confirm, the latter is not accurate. As you state previously this simulant is composed of palagonitized tephra ash. Plagaotinization is an alteration process. It is basically altered basaltic glass, altered from weathering. Although some of the parent minerals are still present, much of the JSC-Mars-1 simulant is composed of secondary materials (many of the x-ray amorphous materials) produced by weathering. In this case, age alone, but also composition, are the necessary indicator for how altered the sample is. In fact, the majority of the Fe(III) in the simulant are likely sourced from weathered material in the simulant, i.e. it has an abundance Fe(III) likely because it has had greater degree of weathering/alteration. (The same is true for Fe(III) on Mars so again this does not invalidate anything, just an important piece of context that should be addressed)*
  - *Mars regolith actually contains SO4 (sulfate), but when in-situ samples are processed in the rover instruments it is volatilized to SO3, so that is what the instruments report it as. Similar to how other oxides are reported. But Mars soil contains sulfate (SO4)*

2c. The statement about environmental influence has been removed. We also improved clarity by adding the following statement: “Martian regolith contains sulfate (avg. 6.16%, detected as sulfur trioxide) and chlorine (avg. 0.68%), neither of which have been detected in our regolith samples by the suppliers or for in the work presented here.”

3. *Important limitations that may need to be additionally discussed*

- *The JSC-Mars-1 is a spectral simulant specifically, and so has spectral characteristics important comparable to Mars. At a proof of concept level it is an appropriate simulant for the purposes you intend and does well at supporting the claims. As the work progressed beyond a proof of concept, there are some important limitations of this simulant, including in the magnetic properties, that may be important to mention here. The Allen et al. 1998 reference does include some of the information, Cannon et al. 2019 (Mars global simulant MGS-1) also may have additional helpful information. The simulant has a large amount of x-ray amorphous phases that contribute to the magnetic properties and to mineralogical and chemical differences from actual Martian soil and regolith and these limitations are important to consider as this and similar work develop. Mentioning the limitations may help bring strength to your own work and provide important information for those seeking to duplicate or continue development of biomining on Mars. Again, for the proof of concept level you are demonstrating it is very useful and provides important insights, but somewhere in the discussion important to consider limitations specifically related to magnetism and whether the magnetic properties of the simulants reflects that of Mars soil.*

3a. We agree with reviewer 1 that it is important to clarify these differences between the regolith simulants used and the actual Martian soil, and we are thankful for the provided literature. The high amount of amorphous states of the simulants are indeed likely to influence their magnetic properties and hence the amount of magnetically extracted material. Other simulants, like the MGS-1 simulant described by Cannon et al.[1], might be less magnetic, but are likely to become more magnetic after bacterial treatment, hence increasing the amount of magnetically extractable material. Future studies with MGS-1 are required to confirm this statement and to find to what extent the amount of magnetically extracted material after bacterial treatment increases for this simulant. A full paragraph based on the reviewers input was added in “Composition and limitations of regolith simulants” to describe the limitations of the regolith simulants and the suggestion for the future use of MGS-1 better:

“It should be taken into account that the regolith simulants used in this study are rich in amorphous phases, while actual Martian soil consists of an approximately equal distribution of amorphous and crystalline phases [25, 33]. The higher amount of amorphous phases influences the magnetic properties of the regolith simulants used. For example, it has been reported that JSC-Mars1 is more magnetic than the actual Martian soil [26], which could affect the amount of magnetic material extracted in our approach. Our method, however, is aimed at quantifying the increase in the amount of magnetically extractable material upon bacterial treatment, rather than determining the exact amount of material we can extract. To study whether regolith simulants containing more crystalline phases are also compatible with our approach, and to potentially better determine the amount of material that can be extracted in future biomining approaches, it would be worthwhile to perform follow-up studies using the regolith simulant MGS-1 [33].”

*The remaining comments for revision focus on smaller scale items rather than items that affect multiple sections, so I will simply go through these in order as they occur in the paper and try to indicate specifically the location and the issue that needs to be addressed.*

- *As a reader, I was a little confused at first to which simulants exactly are being used. This is not specified until further into the paper. It may be useful to indicate this information earlier in the introduction and provide the references for which details on the development or obtaining of the simulants can be found. I would also suggest an additional reference the JSC-Mars simulant may have (Allen et al. 1998 MARTIAN REGOLITH SIMULANT JSC MARS-1.). This is the conference abstract that describe the simulants development and origins.*

4.We agree, therefore we added this table in the introduction to give an improved overview and added the reference for Allen et al.[2]

| ***Simulant*** | ***Commissioned by*** | ***Simulant properties*** |
| --- | --- | --- |
| EAC-1 | European Space Agency | Manick et al. (2018) [3], Schleppi et al. (2019) [4] |
| JSC-2A | NASA Johnson Space Center | Meurisse et al. (2018) [5], Schleppi et al. (2019) [4] |
| JSC-Mars1 | NASA Johnson Space Center | Allen et al. (1998) [2] |

*The abstract*

- *“Application of self-reproducing bacteria…is a promising approach to avoid the pitfalls”*
  - *The word avoid may be a slight overstatement. Bacteria would certainly require less cumbersome equipment, but they do still require equipment to maintain and house them and apply them (for example the 3D printers you mention). It would not eliminate the use of heavy or cumbersome equipment but would certainly help to lessen it.*

5. We agree and therefore changed the word “avoid” into “reduce”.

- *“resulting in a 396 +- 115% improvement…”*
  - *That seems like a very large error range. If this is not a typo, the source of that error range and what that might mean about the results would be important to mention in the discussion*

6. The large error range is due to physical distortions that were observed in the 3D prints sintered from bacterially treated and magnetically extracted material. These distortions caused variations in the structural composition of the 3D prints and hence their ultimate compressive strength. In the new version of our paper, we state a 400% improvement of ultimate compressive strength in the abstract. In the “Ultimate compressive strength of 3D prints” subsection in the Results & Discussion section, where we have the space to explain this large error range, we state the exact increase in ultimate compressive strength with the associated standard deviation. We explain the origin of the large error range here as follows: “The large standard deviation of the increase in ultimate compressive strength was likely due to physical distortions observed in the treated samples, causing a variation in their structural composition. The treated material required a slightly higher temperature to complete sintering (1100°C) than the untreated material (1050°C), such that distortions including porosity clusters and delamination were formed in the 3D prints of the treated material. However, since the difference in sintering temperature was relatively small, we expect that it had little effect on the increase in compressive strength in the treated material.” The difference in sintering temperature is also discussed in this added paragraph, and is discussed later in this response letter under the point “Lithography-based ceramic printing” in the Materials and Methods.

*The Introduction*

- *The paragraph that starts “Traditionally, research in the sector”.*
  - *I am not sure the purpose this paragraph serves in the overall discussion. As a reader it seemed random and unnecessary. I recommend either deleting or providing transition that helps it fit into the context.*

7. We followed the reviewers’ recommendation and deleted this paragraph.

- *“These organisms can be produced in large quantities, only requiring water…growth medium.”*
  - *They also require the equipment needed to house them in (glassware or whatnot), much smaller than the factories and machinery previously mentioned, but still an important piece needed.*

8. We have added new text stating that the method also requires a bioreactor: “These organisms can be produced in large quantities, only requiring water, a bioreactor, and an easily transportable growth medium [5].”

- “*Microbial production…do not require…high energy investment or toxic chemical”*
  - *Some may require toxic chemicals, and some actually produce them. That depends on the microorganism being used. They do decrease the energy and the toxic and have huge potential as you state, but the way the current sentence is phrased may be a bit of an overstatement.*

9. We have changed “Microbial production processes do not require …” into “Microbial production processes do not **necessarily** require …”.

- *“Together, our findings provide the first…”*
  - *This is an important point, but the sentence is rather long and complex so the point becomes a little muddled. Perhaps break it into multiple sentences*

10. We thank reviewer 1 for pointing this out, and we have broken down the sentence into the following: “Together, our findings provide the first proof-of-principle of bio-mediated *in-situ* iron extraction from Martian regolith simulant. Our approach is of sufficient quality to enable on-demand production of infrastructure materials as well as replacement parts for a future space colony.”

*Results and Discussion*

*“Composition and limitations of regolith simulants”*

- *“whereas EAC-1 mined from a deposit…EAC-1 shows alterations…decided to use JSC-2A…in our experiments*
  - *This paragraph of discussion was a little confusing. It tends to go back and forth between the two simulants quite a bit and it is hard to follow as a reader the logic used in choosing among the simulants and why EAC-1 was used if it had alteration while at the same time JSC-2A was used to avoid alteration in JSC-1A. Perhaps a little reorganization and clarification of this discussion.*

11. To clarify our motivation to use EAC-1 and JSC-2A, we reorganized the sentence “JSC-2A in contrast is produced from synthetic minerals [25] as the successor of JSC-1A and shares, therefore,..” into “In contrast to EAC-1, JSC-2A, which is the successor of JSC-1A, is produced from synthetic minerals [25] and therefore shares..” and added the sentence “These differences in composition caused by varying length of exposure time to the elements are valuable for our research since compositional differences are also observed in the Martian bedrock. Furthermore, it can give insights in the most suitable regolith type for our method.”

- *Table 2: There are a few minerals indicated as only present in the magnetically extracted material. Were these minerals after exposure to Schewnella so only present because of treatment, or are these minerals present in the original samples. The tables purpose seems to be to present the original composition so if these minerals are a result of treatment, would they be appropriate to indicate here?*

12. It is difficult to differentiate whether the minerals are a direct effect of the treatment with bacteria or a result of the concentrating of magnetically active components. The bioleaching effect as well as reduction through *Shewanella* and subsequent precipitation can lead to the formation of new minerals (like magnetite) (Perez-Gonzalez et al, 2010, Magnetite biomineralization induced by Shewanella oneidensis)[6]. Magnetite was, however, also found in non-bacterially treated but magnetically extracted material. Based on that we cannot conclude additional information from the table other than that certain minerals become concentrated via magnetic extraction of a portion of the material. For clarification a subsequence was added: “* These elements were only detected in the magnetically extracted fraction of bacterially treated and bacterially untreated material.”

“Toxicity of Regolith simulants in aerobic and anaerobic conditions”

- “This result may reflect the difficulty in obtaining reliable absorbance measurements in the presence of high iron concentrations due to optical properties of iron”
  - Is this difficult specific to iron (II) or iron (III). Also what would allow you to differentiate that this is the cause as apposed to another reason? Stating this will help to support the interpretation of the results.

13. We would like to thank reviewer 1 for pointing this out. This is specific to iron(III), and we have added a reference to confirm this statement (Poulin et al, 2014, Effects of iron on optical properties of dissolved organic matter)[7]. In addition to the fact that it is known that iron(III) can interfere with optical measurements, the statements is further substantiated by the fact that the decreasing trend in growth was not observed for the corresponding CFUs. To clarify this, we changed the cited sentence into “This result may reflect the difficulty in obtaining reliable absorbance measurements in the presence of high iron concentrations due to the optical properties of Fe(III) [34], as this trend was not observed in the corresponding CFU measurements.”

*“Iron and Silicon concentration in the magnetically extracted materials”*

- *Important to clarify which part of the sample you are referring to here. It is unclear if the whole sample, just the extracted portion, or the unextracted portion. As you discuss these results, clarify more clearly which portion a particular result refers to. For example the XRF was it performed on the entire sample or on just the extracted portion or the untreated sample? It can become confusing exactly where extra iron is coming from and silica is disappearing to, but being more specific could help to clarify.*

14. We agree with the reviewer and changed the title from “*Iron and Silicon concentration in the magnetically extracted materials*” into “*Iron and silicon concentration in the treated and untreated regolith simulants”.* Also “bacterially treated” was removed from the sentence “A higher iron oxide and lower silicon oxide concentration are critical to improve the electric as well as mechanical properties of bacterially treated regolith.”, since this is also true for untreated regolith.

To clarify what we mean by “treatment,” the sentence “We postulate that the magnetic treatment will concentrate iron and therefore…” was expanded into “We postulate that the magnetic and bacterial treatment will concentrate iron and therefore...” For the same reason, the sentence “To analyze the weight percent of iron oxides (Fig. 5A) and silicon oxides (Fig. 5B) in different regolith simulant samples, X-ray fluorescence measurements (XRF) were performed to evaluate the compositional changes of this treatment” was changed into “To analyze the weight percent of iron oxides (Fig. 5A) and silicon oxides (Fig. 5B) in treated regolith samples as well as untreated regolith samples, to which no magnetic or bacterial methodology were applied, X-ray fluorescence measurements (XRF) were performed.”
The sentence “XRF measurements were then performed to determine the iron and silicon content of both the untreated regolith samples, to which no magnetic or bacterial methodology were applied, and the bacterially treated samples” was removed because of repetitiveness.

The final sentence “This alteration of the composition upon treatment indicates that not only the quantity but also the quality of extracted material for construction applications were improved through the application of *S. oneidensis*.” was slightly altered into “This alteration of the composition upon treatment indicates that not only the quantity but also the quality of extracted material for construction applications was improved through the application of *S. oneidensis* and magnetic extraction.”

*Conclusion*

- *The conclusions overall go back and forth a lot, particularly noticed in the second paragraph of the section “Bacterial reduction of regolith simulants”. As a reader the order (or lack of) made the topic of discussion is hard to follow, perhaps some reorganization of the information would help.*

15. We reorganized a large part of the conclusion and tried to improve the writing for understandability to walk the reader through the meaning of the results one by one.

*Materials and Methods*

- *Toxicity of regolith simulants*
  - *“previously sterilized regolith samples were sterilized again via autoclaving and diluted to the desired concentration…”*
    - *What was the “previous sterilization” process that was used. Also what temperature of autoclaving. If heated high enough this can affect the iron oxides in the simulant, for example goethite converts to hematite. They are still a source of Fe(III) and Shewnella is very adaptive and can use a variety of different Fe(III) sources, but the kinetics does differ among so can have important affect on the results. Be more specific about how they were sterilized, especially temperature or exposure to oxidizing materials like hydrogen peroxide if used.*

16. The previous sterilization process was also an autoclaving step. To avoid confusion, we have changed the cited section into “Regolith simulant samples (EAC-1, JSC-2A, JSC-Mars1) were sterilized via autoclaving at 121°C …”. Since the sterilization of regolith simulants was relevant for all experiments, we moved this text from the subsection “Toxicity of regolith simulants” to the subsection “Sample preparation”.

# The conversion of goethite to hemathite occurs at ~250°C (Ulrich et al, 2019, The relative distribution of critical (Sc, REE) and transition metals (Ni, Co, Cr, Mn, V) in some Ni-laterite deposits of New Caledonia

)[8], so it is not likely that this occurred during our sterilization procedure. No oxidizing materials were used for autoclaving.

- *Magnetic Extraction apparatus*
  - *Had a hard time picturing what this looks like. May be useful to include a simple schematic or image of the device. Not essential, but if there is ability to include a figure may be useful.*

17. The schematic of this method is provided in figure 3A. We included a reference to this schematic in the methods section.

- *Lithography-baed ceramic printing*
  - *“dried and sintered for 2 hours at 1050C and 1100C for the untreated and treated samples, respectively”*
    - *Why different temperatures, and would this affect the strength of the material? Important to clarify.*

18. As was already discussed under point number 6, a difference in sintering temperature was required to complete full sintering for the treated material. This slight difference in sintering temperature may have contributed to the increase in ultimate compressive strength to some extent. However, since the difference in sintering temperature is relatively small (and the increase in ultimate compressive strength very large), we expect that the increase in ultimate compressive strength is mainly due to bacterial treatment and the therefore increased iron content in the magnetically extracted sample.

*Other comments*

- *Some additional references that could be useful that are not cited here include Cockell 2010 and 2011 about Geobiology in space. The author presents some interesting discussion of biomining and bio-extraction in ISRU applications of space exploration. Considering the topic, are important references to consider.*

19. We added Cockell et al. (2010)[9] as a reference in our introduction.

- *Overall a very interesting study with potential to contribute very significant work to ISRU applications. There are some major revision needed before ready for publication, but can tell it will be a significant contribution to the literature.*

20. We are grateful for all the valuable additions made by the reviewer and hope that the revisions are in agreement with the reviewer.

**Reviewer 2**

*The manuscript reports on a novel approach to extract Iron from Lunar and Martian simulant material using Shewanella oneidensis bacteriun. An additional bonus is included by demonstrating the increased material strength properties of subsequently 3d printed test articles. As a demonstration of biology driven ISRU process, this work is highly novel and the results would be of great interest to those working on novel ISRU approaches.*

21. We thank the reviewer for the kind evaluation and underlining the importance of this work.

**References**

[1]	K. M. Cannon, D. T. Britt, T. M. Smith, R. F. Fritsche, and D. Batcheldor, “Mars global simulant MGS-1: A Rocknest-based open standard for basaltic martian regolith simulants,” *Icarus*, vol. 317, pp. 470–478, Jan. 2019.

[2]	Carlton C. Allen *et al.*, “Martian Regolith Simulant JSC Mars-1,” *Lunar Planet. Sci. XXIX*, no. Table 2, pp. 4–5, 2004.

[3]	K. Manick, S.-J. Gill, J. Najorka, C. L. Smith, and L. Duvet, “FUNDAMENTAL PROPERTIES CHARACTERISATION OF LUNAR REGOLITH SIMULANTS AT THE EUROPEAN SPACE AGENCY (ESA) SAMPLE ANALOGUE CURATION FACILITY.,” vol. 2018, no. 2083, pp. 2–3, 2018.

[4]	J. Schleppi, J. Gibbons, A. Groetsch, J. Buckman, A. Cowley, and N. Bennett, “Manufacture of glass and mirrors from lunar regolith simulant,” *J. Mater. Sci.*, vol. 54, no. 5, pp. 3726–3747, 2019.

[5]	A. Meurisse, A. Makaya, C. Willsch, and M. Sperl, “Solar 3D printing of lunar regolith,” *Acta Astronaut.*, 2018.

[6]	T. Perez-Gonzalez *et al.*, “Magnetite biomineralization induced by Shewanella oneidensis,” *Geochim. Cosmochim. Acta*, vol. 74, pp. 967–979, 2010.

[7]	B. A. Poulin, J. N. Ryan, and G. R. Aiken, “Effects of iron on optical properties of dissolved organic matter,” *Environ. Sci. Technol.*, vol. 48, no. 17, pp. 10098–10106, 2014.

[8]	M. Ulrich, M. Cathelineau, M. Muñoz, M. C. Boiron, Y. Teitler, and A. M. Karpoff, “The relative distribution of critical (Sc, REE) and transition metals (Ni, Co, Cr, Mn, V) in some Ni-laterite deposits of New Caledonia,” *J. Geochemical Explor.*, vol. 197, no. April 2018, pp. 93–113, 2019.

[9]	C. S. Cockell, “Geomicrobiology beyond Earth: Microbe-mineral interactions in space exploration and settlement,” *Trends in Microbiology*, vol. 18, no. 7. Elsevier Current Trends, pp. 308–314, 01-Jul-2010.
